# Supplementary material for: Non-clinical Safety and Efficacy of an AAV2/8 Vector Administered Intravenously for Treatment of Mucopolysaccharidosis Type VI
Source: Mol Ther Methods Clin Dev. 2017 Jul 24;6:143–58. doi: 10.1016/j.omtm.2017.07.004 (PMC5552066; doi:10.1016/j.omtm.2017.07.004)
Supplement: Document S1. Figure S1 and Tables S1–S11 [file mmc1.pdf]

OMTM, Volume *6*

## Supplemental Information

# Non-clinical Safety and Efficacy of an AAV2/8 Vector Administered Intravenously for Treatment of Mucopolysaccharidosis Type VI

**Rita Ferla, Marialuisa Alliegro, Jean-Brice Marteau, Margherita Dell'Anno, Edoardo Nusco, Severine Pouillot, Stefania Galimberti, Maria Grazia Valsecchi, Vincent Zuliani, and Alberto Auricchio**

**Figure S1. Body weight changes of C57/BL6-Tg*ARSBC91S* mice treated with AAV2/8.TBG.*hARSB*.**

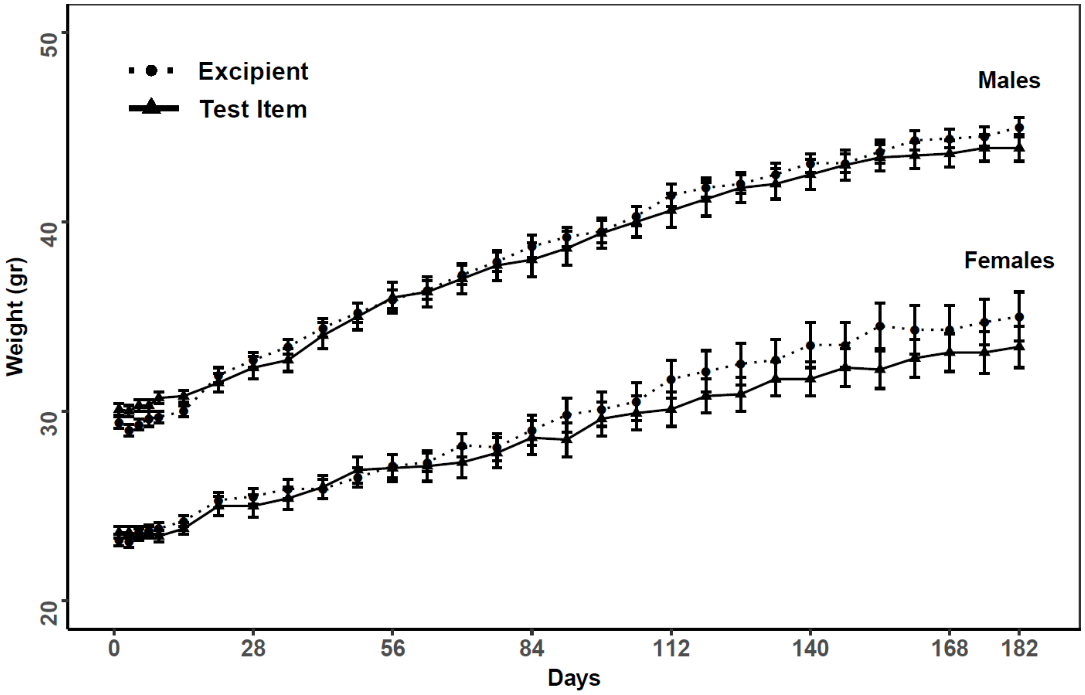


Body weight was recorded on D1 (pre-dose), D3, D5, D7, D9, D14 and then weekly both in male and female mice. Results are reported as mean ± SEM. The comparison of the treatment specific growth curves in males and females was performed by means of an ANOVA for repeated measurements. The interaction time*treatment was not found statistically significant ($p$-value>0.05). The number of animals is: *Males*, n=46 from D1 to D14, n=26 from D21 to D182 in both the excipient and the test item groups; *Females*: n=46 from D1 to D14, n=26 from D21 to D182 in both the excipient group; n=45 from D1 to D14 (except D3, n=44), n=25 from D21 to D182 in the test item group. *Abbreviations:* Excipient, formulation buffer diluted 1:1.5 in NaCl 0.9% saline solution; Test Item, AAV2/8.TBG.*hARSB* diluted 1:1.5 in NaCl 0.9% saline solution (dose administered $2x10^{13}$ GC/kg).

**Table S1. Hematology profile in mice following systemic administration of either AAV2/8.TBG.*hARSB* or the excipient.**

| | **D15** | | **D180** | |
|---|---|---|---|---|
| | Excipient | Test item | Excipient | Test item |
| **Males** | n=9 | n=9 | n=10 | n=9 |
| WBC | 6.60 (5.90-7.70) | 7.00 (6.70-7.40) | 8.25 (7.30-9.40) | 8.30 (7.50-9.40) |
| NEUT | 0.87 ± 0.17 | 0.70 ± 0.09 | 0.90 ± 0.19 | 0.84 ± 0.19 |
| EOSI | 0.11 ± 0.04 | 0.14 ± 0.07 | 0.14 ± 0.08 | 0.10 ± 0.03 |
| BASO | 0.02 ± 0.01 | 0.02 ± 0.01 | 0.03 ± 0.01 | 0.04 ± 0.01 |
| LYMP | 5.60 (4.90-6.80) | 5.80 (5.80-6.30) | 7.10 (6.10-7.70) | 7.10 (6.60-8.10) |
| MONO | 0.06 ± 0.03 | 0.08 ± 0.03 | 0.15 ± 0.05 | 0.10 ± 0.05 |
| LUC | 0.06 ± 0.02 | 0.06 ± 0.02 | 0.10 ± 0.04 | 0.11 ± 0.05 |
| RBC | 10.78 ± 0.74 | 11.33 ± 0.54 | 10.53 ± 0.25 | 10.73 ± 0.25 |
| HGB | 15.41 ± 0.68 | 15.89 ± 0.90 | 15.07 ± 0.52 | 15.16 ± 0.52 |
| HCT | 48.67 ± 2.87 | 51.22 ± 1.72 * | 47.10 ± 1.73 | 48.67 ± 1.58 |
| MCV | 45.22 ± 0.97 | 45.22 ± 0.97 | 45.00 ± 1.15 | 45.33 ± 1.00 |
| MCH | 14.36 ± 0.89 | 14.09 ± 1.20 | 14.34 ± 0.3 | 14.10 ± 0.23 |
| MCHC | 31.67 ± 1.73 | 31.00 ± 2.50 | 32.00 ± 0.47 | 31.22 ± 0.44 ** |
| RET | 298.22 ± 34.75 | 287.22 ± 23.83 | 292.80 ± 37.07 | 307.33 ± 21.97 |
| RDW | 12.61 ± 0.73 | 12.11 ± 0.28 | 13.32 ± 0.58 | 13.41 ± 0.32 |
| THR | 1165 (1109-1292) | 1368 (1094-1433) | 1211 (1093-1249) | 1440 (1373-1543) ** |
| MPV | 5.48 ± 0.67 | 5.57 ± 0.57 | 5.51 ± 0.59 | 7.37 ± 0.26 ** |
| **Females** | n=7 | n=8 | n=10 | n=9 |
| WBC | 5.80 (3.80-6.10) | 5.30 (4.60-6.85) | 3.30 (2.50-3.80) | 3.30 (2.50-3.40) |
| NEUT | 0.84 ± 0.23 | 0.82 ± 0.24 | 0.44 ± 0.14 | 0.42 ± 0.11 |
| EOSI | 0.09 ± 0.08 | 0.07 ± 0.04 | 0.04 ± 0.02 | 0.06 ± 0.04 |
| BASO | 0.02 ± 0.01 | 0.03 ± 0.01 | 0.02 ± 0.01 | 0.01 ± 0.01 |
| LYMP | 4.70 (3.30-4.90) | 4.35 (3.55-5.65) | 2.70 (2.20-3.30) | 2.5 0(2.00-2.90) |
| MONO | 0.04 ± 0.02 | 0.05 ± 0.02 | 0.03 ± 0.02 | 0.06 ± 0.04 |
| LUC | 0.03 ± 0.01 | 0.03 ± 0.02 | 0.02 ± 0.01 | 0.03 ± 0.03 |
| RBC | 10.84 ± 0.39 | 10.83 ± 0.20 | 10.33 ± 0.27 | 10.23 ± 0.34 |
| HGB | 16.36 ± 0.50 | 16.15 ± 0.39 | 14.99 ± 0.53 | 14.98 ± 0.68 |
| HCT | 51.29 ± 2.14 | 50.88 ± 1.46 | 46.2 ± 1.55 | 46.44 ± 1.94 |
| MCV | 47.43 ± 0.79 | 47.00 ± 0.76 | 44.70 ± 1.06 | 45.44 ± 0.73 |
| MCH | 15.10 ± 0.28 | 14.93 ± 0.17 | 14.50 ± 0.29 | 14.64 ± 0.31 |
| MCHC | 32.29 ± 0.49 | 31.75 ± 0.46 | 32.50 ± 0.71 | 32.22 ± 0.67 |
| RET | 280.14 ± 37.75 | 294.75 ± 28.01 | 308.80 ± 54.07 | 352.56 ± 55.21 |
| RDW | 12.37 ± 0.41 | 12.18 ± 0.47 | 12.85 ± 0.57 | 12.88 ± 0.37 |
| THR | 1008 (938-1168) | 1083.5 (999-1162) | 1213 (1101-1462) | 1133 (1122-1344) |
| MPV | 6.06 ± 0.47 | 5.74 ± 0.65 | 6.03 ± 0.65 | 7.23 ± 0.36 ** |

Hematology was assessed in male and female mice on D15 and D180 by treatment group. Data are reported as mean ± SD and the comparison between treatment groups (at different time points in males and females) were performed by means of the t-test or reported as median (I-III quartile) and compared by means of the Wilcoxon-Mann-Whitney test (for WBC, LYMP and THR). The *p*-value: * ≤ 0.05, ** ≤ 0.01. *Abbreviations and units:* WBC (Giga/L) = white blood cell count; NEUT (Giga/L) = neutrophils; EOSI (Giga/L) = eosinophils; BASO (Giga/L) = basophils; LYMP (Giga/L) = lymphocytes; MONO (Giga/L) = monocytes; LUC (Giga/L) = large unstained cells; RBC (Tera/L) = red blood cell count; HGB (g/100 mL) = haemoglobin; HCT (%) = haematocrit; MCV (fl) = Mean corpuscular volume; MCH (pg) = mean corpuscular haemoglobin; MCHC (g/100 mL) = mean corpuscular haemoglobin concentration; RET (Giga/L) = reticulocytes; RDW (%) = red cell (erythrocyte) volume; THR (Giga/L) = thrombocytes; MPV (%) = mean platelet thrombocyte volume. Excipient, formulation buffer diluted 1:1.5 in NaCl 0.9% saline solution; Test Item, AAV2/8.TBG.*hARSB* diluted 1:1.5 in NaCl 0.9% saline solution (dose administered $2x10^{13}$GC/kg).

**Table S2. Blood chemistry profile in mice following systemic administration of either AAV2/8.TBG.*hARSB* or the excipient.**

| | n | D15 Excipient | n | Test item | n | D180 Excipient | n | Test item |
|---|---|---|---|---|---|---|---|---|
| ***Males*** | | | | | | | | |
| ALT | 15 | 51 (39-72) | 15 | 41 (36-61) | 16 | 120 (73-172) | 16 | 155.5 (89-195.50) |
| AST | 15 | 85 (68-107) | 14 | 79.5 (64-108) | 16 | 105 (89.50-156.50) | 16 | 130 (91.50-155.5) |
| ALP | 15 | 82 (73-91) | 15 | 89 (81-94) | 16 | 83.50 (69.50-105) | 16 | 98 (86.50-123) |
| CK | 15 | 384 (261-640) | 13 | 360 (215-519) | 16 | 196 (114-411.50) | 16 | 191 (125.50-301) |
| ALB | 15 | 28.53 ± 1.46 | 15 | 29.8 ± 1.42 | 16 | 32.25 ± 1.61 | 16 | 32.31 ± 1.08 |
| TBIL | 12 | 1.71 ± 0.27 | 12 | 1.86 ± 0.42 | 15 | 1.60 ± 0.22 | 15 | 1.46 ± 0.20 |
| CHOL | 13 | 2.61 ± 0.27 | 13 | 2.45 ± 0.13 | 16 | 3.72 ± 0.66 | 16 | 3.84 ± 0.85 |
| CREA | 13 | 20 (13-22) | 13 | 17 (15-21) | 16 | 18.5 (15.5-21.5) | 16 | 18.5 (16-22) |
| GLU | 15 | 11.88 ± 2.12 | 15 | 13.44 ± 2.16 | 16 | 13.74 ± 2.7 | 16 | 13.40 ± 1.87 |
| PROT | 15 | 53.47 ± 3.36 | 13 | 58.54 ± 3.57 ** | 16 | 58.38 ± 2.03 | 16 | 59.38 ± 2.22 |
| TRIG | 13 | 0.92 ± 0.19 | 13 | 1.03 ± 0.23 | 16 | 0.86 ± 0.12 | 16 | 0.91 ± 0.15 |
| UREA | 13 | 7.54 ± 1.31 | 13 | 7.76 ± 1.16 | 16 | 6.47 ± 1.12 | 16 | 6.64 ± 1.55 |
| P | 13 | 2.09 ± 0.36 | 13 | 2.11 ± 0.44 | 16 | 1.86 ± 0.31 | 16 | 1.92 ± 0.26 |
| Cl | 13 | 111.46 ± 3.60 | 13 | 111.38 ± 4.75 | 16 | 112.69 ± 1.66 | 16 | 112.25 ± 4.16 |
| K | 13 | 4.65 ± 0.58 | 13 | 4.96 ± 0.71 | 16 | 3.86 ± 0.34 | 16 | 4.24 ± 0.46 |
| Na | 13 | 150.62 ± 4.31 | 13 | 149.62 ± 3.28 | 16 | 147 ± 2.90 | 16 | 147.75 ± 1.81 |
| GLOB | 15 | 24.93 ± 3.20 | 13 | 28.62 ± 3.12 ** | 16 | 26.13 ± 1.36 | 16 | 27.06 ± 2.14 |
| A/G | 15 | 1.17 ± 0.20 | 13 | 1.05 ± 0.10 | 16 | 1.24 ± 0.09 | 16 | 1.20 ± 0.11 |
| **Females** | | | | | | | | |
| ALT | 20 | 49 (42.50-67) | 21 | 65 (54-94) * | 16 | 52.50 (43.50-70) | 16 | 48.50 (37-72.50) |
| AST | 18 | 112.50 (76-167) | 21 | 131 (102-166) | 16 | 104.50 (86-116) | 16 | 81.5 (74-107) |
| ALP | 20 | 131.50 (106-141) | 21 | 122 (115-134) | 16 | 90.5 (79.50-105) | 16 | 93 (78-110.50) |
| CK | 19 | 819 (350-1713) | 18 | 778.50 (490-1247) | 16 | 262.50 (159-361.50) | 16 | 288.50 (183-317) |
| ALB | 20 | 30.40 ± 1.70 | 21 | 29.76 ± 20 | 16 | 30.44 ± 1.26 | 16 | 30.94 ± 1.39 |
| TBIL | 11 | 1.66 ± 0.36 | 10 | 1.50 ± 0.26 | 8 | 1.58 ± 0.69 | 10 | 1.74 ± 0.48 |
| CHOL | 20 | 2.29 ± 0.24 | 21 | 2.10 ± 0.20 ** | 16 | 2.12 ± 0.37 | 16 | 2.18 ± 0.27 |
| CREA | 12 | 18.5 (16.50-20.50) | 13 | 15 (15-20) | 16 | 19 (16.50-20.50) | 16 | 19 (16.50-20) |
| GLU | 13 | 9.40 ± 2.05 | 13 | 9.22 ± 2.46 | 16 | 9.76 ± 1.51 | 16 | 10.63 ± 1.93 |
| PROT | 18 | 55.28 ± 2.44 | 19 | 56.21 ± 4.32 | 16 | 55.94 ± 2.32 | 16 | 56.25 ± 2.79 |
| TRIG | 13 | 0.77 ± 0.22 | 13 | 0.82 ± 0.22 | 16 | 0.85 ± 0.28 | 16 | 0.89 ± 0.37 |
| UREA | 13 | 7.06 ± 1.19 | 13 | 7.37 ± 0.76 | 16 | 5.73 ± 1.28 | 16 | 5.96 ± 1.52 |
| P | 13 | 2.07 ± 0.35 | 13 | 2.21 ± 0.31 | 16 | 1.94 ± 0.32 | 16 | 1.94 ± 0.29 |
| Cl | 13 | 105.85 ± 5.29 | 13 | 107.38 ± 5.27 | 16 | 109.31 ± 3.18 | 16 | 108.25 ± 4.93 |
| K | 13 | 4.77 ± 0.60 | 13 | 5.30 ± 0.97 | 16 | 3.80 ± 0.31 | 16 | 3.98 ± 0.40 |
| Na | 13 | 153.38 ± 5.01 | 13 | 147.46 ± 4.61 ** | 16 | 146.63 ± 1.82 | 16 | 145.38 ± 2.66 |
| GLOB | 18 | 24.78 ± 2.44 | 19 | 26.47 ± 3.17 | 16 | 25.50 ± 1.79 | 16 | 25.31 ± 2.21 |
| A/G | 18 | 1.25 ± 0.16 | 19 | 1.13 ± 0.13 | 16 | 1.20 ± 0.09 | 16 | 1.23 ± 0.11 |

Blood chemistry was assessed in male and female mice on D15 and D180 by treatment group. Data are reported as mean ± SD and the comparison between treatment groups (at different time points in males and females) were performed by means of the t-test or reported as median (I-III quartile) and compared by means of the Wilcoxon-Mann-Whitney test (for ALT, AST, ALP, CK and CREA) . The *p*-value: $^{*} \leq 0.05$, $^{**} \leq 0.01$. *Abbreviations and units*: ALT (IU/L) = alanine aminotransferase; AST (IU/L) = aspartate aminotransferase; ALP (IU/L) = alkaline phosphatase. CK (IU/L) = creatine kinase; Metabolites and proteins: ALB (g/L) = albumin; TBIL (μmol/L) = total bilirubin; CHOL (mmol/L) = total cholesterol; CREA (μmol/L) = creatinine; GLU (mmol/L) = glucose; PROT (g/L) = total proteins; TRIG (mmol/L) = triglycerides; UREA (mmol/L) = urea; GLOB (g/L) = globulina; A/G = ration albumin/globulina; Electrolytes: P (mmol/L) = phosphorus; Cl (mmol/L) = chloride; K (mmol/L) = potassium; Na (mmol/L) = sodium; Limit of quantification of TBIL: 1.13 μmol/L; Limit of quantification of CREA: 8.7 μmol/L. Excipient, formulation buffer diluted 1:1.5 in saline solution NaCl 0.9%; Test Item, AAV2/8.TBG.*hARSB* diluted 1:1.5 in saline solution NaCl 0.9% (dose administered $2x10^{13}$ GC/kg).

**Table S3. Macroscopic findings observed at necropsy**

| | Organ | Observation | Excipient | Test Item | Notes from Histopathology report |
|---|---|---|---|---|---|
| **Males D15** | *Spleen* | Black area (2 mm diameter) | 1/5 | 1/5 | Excipient group: no observable microscopic abnormalities.<br>Test item group: mild spleen congestion. |
| **Males D180** | *Spleen* | Black area (2 mm diameter) | 1/5 | 0/5 | Mild spleen congestion |
| | *Liver* | Pale | 2/5 | 3/5 | Excipient group: moderate (n=1) or marked (n=1) periacinar hepatocytic lipidosis.<br>Test item group: moderate (n=2) or marked (n=1) periacinar hepatocytic lipidosis (n=1). |
| | *Sub-maxillary lymph nodes* | Red Dark | 1/5 | 0/5 | No observable microscopic abnormalities |
| **Females D15** | *Spleen* | Black area (2 mm diameter) | 1/5 | 1/5 | Excipient group: moderate spleen congestion.<br>Test item group: no observable microscopic abnormalities. |
| | *Gall bladder* | Description not available | 0/5 | 1/5 | Not available because of an error during harvesting |
| **Females D180** | *Spleen* | Three black spots | 0/5 | 1/5 | Mild spleen congestion |
| | | Black area (2-3 mm diameter) | 0/5 | 2/5 | No observable microscopic abnormalities. |
| | | Black area (half surface of the organ) | 0/5 | 1/5 | No observable microscopic abnormalities. |
| | *Uterus with uterine cervix* | Red Mass (left, 3 mm diameter) | 0/5 | 1/5 | Mass diagnosis was adenoma (Benign neoplasm). |
| | | Decreased | 1/5 | 0/5 | No observable microscopic abnormalities. |
| | | Enlarged with abnormal content | 0/5 | 1/5 | Dilatation |
| | *Vagina* | Enlarged with abnormal content | 0/5 | 1/5 | Dilatation |
| | *Ovaries* | One enlarged | 1/5 | 0/5 | No observable microscopic abnormalities. |

*Abbreviations:* Excipient, formulation buffer diluted 1:1.5 in NaCl 0.9% saline solution; Test Item, AAV2/8.TBG.*hARSB* diluted 1:1.5 in NaCl 0.9% saline solution (dose administered $2x10^{13}$ GC/kg). The comparison of macroscopic finding incidence in the two treatment groups at each time point in males and females was performed using the Fisher test ($p$-values >0.05).

**Table S4. Incidences and severity of microscopic findings in thyroid of treated mice on D15 and D180**

| Observation | Sex | Mean severity scores (incidence) | | | |
|---|---|---|---|---|---|
| | | D15 | | D180 | |
| | | **Excipient** | **Test item** | **Excipient** | **Test item** |
| Pallid follicular epithelium hypertrophy | M | *(0/5)* | 1.4 *(4/5)** | 0.8 *(3/5)* | 1.4 *(4/5)* |
| | F | *(0/5)* | 0.4 *(3/5)* | 0.4 *(1/5)* | 2.468 *(4/5)* |
| Multifocal follicular epithelial hyperplasia | M | *(0/5)* | *(0/5)* | 0.6 *(1/5)* | 0.8 *(1/5)* |
| | F | *(0/5)* | *(0/5)* | 0.268 *(1/5)* | 2.2 *(3/5)* |
| Lipid replacement od follicular epithelium | M | *(0/5)* | *(0/5)* | *(0/5)* | 0.8 *(3/5)* |
| | F | *(0/5)* | *(0/5)* | *(0/5)* | *(0/5)* |

The mean severity score is calculated as the mean of severity scores of each animal showing the reported observation per each group of treatment. The comparison of microscopic findings incidence in thyroids of treatment groups at each time points in males and females was performed using the Fisher test. The *p*-value vs excipient group: * $\leq$ 0.05. *Abbreviations:* Excipient, formulation buffer diluted 1:1.5 in NaCl 0.9% saline solution; Test Item, AAV2/8.TBG.*hARSB* diluted 1:1.5 in NaCl 0.9% saline solution (dose administered $2x10^{13}$ GC/kg); M, males; F, females.

**Table S5. Vector genome copies in liver, thyroid/parathyroid and pituitary gland from mice of the toxicity study.**

| Organ | D15 | | D180 | |
|---|---|---|---|---|
| | Males | Females | Males | Females |
| **Liver** | $1.9x10^7 \pm 4.9x10^6$ | $9.3x10^6 \pm 3.3x10^6$ | $1.1\ x10^7 \pm 3.6x10^6$ | $5.6x10^6 \pm 1.5x10^6$ |
| **Thyroid/parathyroid** | $1.7x10^4 \pm 8.2x10^3$ | $1.1x10^4 \pm 3.6\ x10^3$ | $3.9x10^3 \pm 1.8x10^3$ | $7.0x10^3 \pm 6.1x10^3$ |
| **Pituitary Gland** | $6.4x10^3 \pm 1.8x10^3$ | $1.4x10^4 \pm 1.3x10^4$ | $1.2x10^3 \pm 5.4x10^2$ | $4.6x10^3 \pm 2.2x10^3$ |

AAV2/8.TBG.*hARSB* (AAV) DNA is expressed as genome copies (GC) per μg of total DNA (GC/μg of total DNA). Results are reported as mean ± SD. *Abbreviations*: D, study day.

**Table S6. AAV2/8.TBG.*hARSB* biodistribution in male mice.**

| Organ | D15 AAV DNA | >LOQ (n) | <LOQ and >LOD (n) | <LOD (n) | D180 AAV DNA | > LOQ (n) | <LOQ and >LOD (n) | <LOD (n) |
|---|---|---|---|---|---|---|---|---|
| **LI** | $2.22\times10^{6}\pm6.46\times10^{5}$ | 4 | 0 | 0 | $1.02\times10^{6}\pm3.04\times10^{5*}$ | 5 | 0 | 0 |
| **GBL** | $9.79\times10^{4}\pm6.42\times10^{4}$ | 4 | 0 | 0 | $6.15\times10^{4}\pm7.29\times10^{4}$ | 5 | 0 | 0 |
| **AD** | $1.91\times10^{4}\pm7.50\times10^{3}$ | 4 | 0 | 0 | $4.73\times10^{3}\pm4.91\times10^{3*}$ | 5 | 0 | 0 |
| **SP** | $7.79\times10^{3}\pm1.14\times10^{4}$ | 4 | 0 | 0 | $8.49\times10^{1}\pm2.86\times10^{1**}$ | 2 | 3 | 0 |
| **AO** | $4.42\times10^{3}\pm1.23\times10^{3}$ | 4 | 0 | 0 | $6.47\times10^{2}\pm2.93\times10^{2**}$ | 5 | 0 | 0 |
| **BL** | $4.01\times10^{3}\pm1.66\times10^{3}$ | 4 | 0 | 0 | <LOD** | 0 | 0 | 5 |
| **KI** | $3.00\times10^{3}\pm1.59\times10^{3}$ | 4 | 0 | 0 | $1.58\times10^{2}\pm2.66\times10^{1**}$ | 5 | 0 | 0 |
| **RE** | $2.26\times10^{3}\pm2.41\times10^{2}$ | 4 | 0 | 0 | $1.98\times10^{2}\pm1.16\times10^{2*}$ | 4 | 1 | 0 |
| **GTHY** | $1.95\times10^{3}\pm1.84\times10^{3}$ | 4 | 0 | 0 | $1.15\times10^{2}\pm4.99\times10^{1**}$ | 3 | 2 | 0 |
| **ILN** | $1.78\times10^{3}\pm5.76\times10^{2}$ | 4 | 0 | 0 | $6.26\times10^{2}\pm4.13\times10^{2**}$ | 5 | 0 | 0 |
| **OES** | $1.52\times10^{3}\pm7.73\times10^{2}$ | 4 | 0 | 0 | $3.03\times10^{2}\pm1.24\times10^{2**}$ | 5 | 0 | 0 |
| **SK** | $1.51\times10^{3}\pm9.13\times10^{2}$ | 4 | 0 | 0 | $8.47\times10^{1}\pm1.49\times10^{1**}$ | 3 | 1 | 1 |
| **GLA** | $1.48\times10^{3}\pm1.03\times10^{3}$ | 4 | 0 | 0 | $2.34\times10^{2}\pm1.38\times10^{1**}$ | 3 | 2 | 0 |
| **HE** | $1.48\times10^{3}\pm5.58\times10^{2}$ | 4 | 0 | 0 | $2.18\times10^{2}\pm9.69\times10^{1**}$ | 5 | 0 | 0 |
| **TRA** | $1.19\times10^{3}\pm7.04\times10^{2}$ | 4 | 0 | 0 | $9.82\times10^{1}\pm3.74\times10^{1**}$ | 5 | 0 | 0 |
| **FOR** | $9.37\times10^{2}\pm7.67\times10^{2}$ | 4 | 0 | 0 | $9.12\times10^{2}\pm1.11\times10^{3}$ | 4 | 1 | 0 |
| **DU** | $8.77\times10^{2}\pm2.97\times10^{2}$ | 4 | 0 | 0 | $1.11\times10^{2}\pm6.61\times10^{1**}$ | 4 | 1 | 0 |
| **SM** | $7.26\times10^{2}\pm3.55\times10^{2}$ | 4 | 0 | 0 | $6.23\times10^{1}\pm2.16\times10^{0**}$ | 3 | 2 | 0 |
| **JE** | $6.69\times10^{2}\pm3.01\times10^{2}$ | 4 | 0 | 0 | $7.93\times10^{1}\pm1.76\times10^{1**}$ | 4 | 1 | 0 |
| **TH** | $6.30\times10^{2}\pm7.25\times10^{2}$ | 4 | 0 | 0 | $4.66\times10^{2}$ | 1 | 3 | 1 |
| **LTEP** | $6.03\times10^{2}\pm2.71\times10^{2}$ | 4 | 0 | 0 | $6.48\times10^{1**}$ | 1 | 4 | 0 |
| **LTE** | $5.90\times10^{2}\pm2.44\times10^{2}$ | 4 | 0 | 0 | <LOQ** | 0 | 5 | 0 |
| **LHEP** | $5.76\times10^{2}\pm1.78\times10^{2}$ | 4 | 0 | 0 | $6.87\times10^{1**}$ | 1 | 2 | 2 |
| **PIT** | $5.53\times10^{2}\pm1.97\times10^{2}$ | 4 | 0 | 0 | $5.90\times10^{1}\pm2.87\times10^{0**}$ | 2 | 3 | 0 |
| **URB** | $5.02\times10^{2}\pm4.17\times10^{2}$ | 4 | 0 | 0 | $5.42\times10^{1**}$ | 1 | 0 | 4 |
| **LU** | $4.90\times10^{2}\pm1.08\times10^{2}$ | 4 | 0 | 0 | <LOQ** | 0 | 4 | 1 |
| **IL** | $4.75\times10^{2}\pm2.03\times10^{2}$ | 4 | 0 | 0 | $5.67\times10^{1}$ | 1 | 4 | 0 |
| **STR** | $4.26\times10^{2}\pm2.35\times10^{2}$ | 4 | 0 | 0 | $3.89\times10^{2}\pm5.96\times10^{2}$ | 5 | 0 | 0 |
| **MLN** | $3.32\times10^{2}\pm2.84\times10^{2}$ | 4 | 0 | 0 | $1.54\times10^{2}\pm1.30\times10^{2*}$ | 2 | 3 | 0 |
| **PRO** | $3.13\times10^{2}\pm1.31\times10^{2}$ | 4 | 0 | 0 | $9.25\times10^{1**}$ | 1 | 3 | 1 |
| **PAN** | $2.85\times10^{2}\pm1.21\times10^{2}$ | 4 | 0 | 0 | <LOQ** | 0 | 4 | 1 |
| **SA** | $2.75\times10^{2}\pm1.51\times10^{2}$ | 4 | 0 | 0 | <LOQ** | 0 | 2 | 3 |
| **CC** | $2.68\times10^{2}\pm7.51\times10^{1}$ | 4 | 0 | 0 | $6.43\times10^{1**}$ | 1 | 4 | 0 |
| **CO** | $2.44\times10^{2}\pm8.12\times10^{1}$ | 4 | 0 | 0 | <LOQ** | 0 | 3 | 2 |
| **PNE** | $2.20\times10^{2}$ | *1* | *3* | 0 | $5.74\times10^{2}$ | 1 | 0 | 4 |
| **STE** | $2.08\times10^{2}\pm7.79\times10^{1}$ | 4 | 0 | 0 | <LOQ** | 0 | 1 | 4 |
| **ENC** | $2.07\times10^{2}\pm3.66\times10^{1}$ | 4 | 0 | 0 | <LOD** | 0 | 0 | 5 |
| **BM** | $1.48\times10^{2}\pm2.34\times10^{1}$ | 3 | 1 | 0 | <LOD | 0 | 0 | 5 |
| **CSC** | $1.35\times10^{2}\pm1.34\times10^{1}$ | 3 | 1 | 0 | <LOD** | 0 | 0 | 5 |
| **LSC** | $1.22\times10^{2}\pm4.28\times10^{1}$ | 4 | 0 | 0 | <LOD** | 0 | 0 | 5 |
| **LAC** | $1.05\times10^{2}\pm2.26\times10^{1}$ | 4 | 0 | 0 | <LOQ** | 0 | 1 | 4 |
| **SV** | $7.69\times10^{1}\pm2.02\times10^{1}$ | 4 | 0 | 0 | $3.63\times10^{2}$ | 1 | 0 | 4 |
| **EY** | $6.32\times10^{1}\pm9.51\times10^{0}$ | 3 | 1 | 0 | <LOQ* | 0 | 2 | 3 |
| **CER** | <LOQ | 0 | 4 | 0 | <LOD** | 0 | 0 | 5 |

AAV2/8.TBG.*hARSB* (AAV) DNA is expressed as genome copies (GC) per μg of total DNA (GC/μg of total DNA). Results are reported as mean ± SD of > LOQ values and in a decreasing order on D15. The number (n) of animals analyzed per each time point is 5. One mice sacrificed on D15 was excluded from statistical analysis because data deviate importantly from other animals likely because test item administration was not optimal. The comparison between AAV DNA on D15 vs D180 was performed using the one sided Wilcoxon-Mann-Whitney test, assuming LOQ and LOD values for analysis. The *p*-value vs. D15 is: * <0.05 and ** < 0.01. Samples below the limit of quantification (LOQ) of the assay are reported as <LOQ. The LOQ is 50 GC/μg of DNA except for: *PNE* (LOQ is 167 in 2 out 5 and 249 GC/μg of total DNA in 1 out 5 animals on D15; *CSL* (LOQ is 100 GC/μg of total DNA in 1 out 5 animals on D15). Samples below the limit of detection (LOD) of the assay are reported as <LOQ. The LOD is 15 GC/μg of total DNA except for: *CSC* (LOD is 29.4 in 1 out 5. 49 in 1 out 5 and 30.6 in 1 out 5 animals; *PNE* (LOD is 15.4 in 1 out 5 animals). LOQ and LOD were recalculated based on the amount of DNA analyzed. i.e. when lower than 600 ng. *Abbreviations*: AAV, Adeno-associated viral vector; D, study day; LI, Liver; GBL, Gall bladder; SA, Salivary gland (sublingual); OES, Oesophagus; FOR, Forestomach; GLA,

Glandular stomach; DU, Duodenum; JE, Jejunum; IL, Ileum incl. Peyer's patches; CC, Caecum; CO, Colon; RE, Rectum; PAN, Pancreas; TH, Thymus; SP, Spleen; MLN, Mesenteric lymph node; ILN, Inguinal lymph node; KI, Kidney; URB, Urinary bladder; TE, Testes; HEP, Head of epididymis; TEP, Tail of epididymis; SV, Seminal vesicles (with coagulating gland); PRO, Prostate; GTHY, Thyroids with parathyroids; AD, Adrenal; PIT, Pituitary; HE, Heart; AO, Aorta; TRA, Trachea; LU, Lungs (incl. bronchi and bronchioles); ENC, brain (encephalus); CER, brain (cerebellum); STE, brain stem; CSC, cervical spinal cord; LSC, lumbar spinal cord; PNE, peripheral nerve (sciatic); EY, Eye; LAC, lachrymal gland (harderian gland); SM, skeletal muscle; STR, sternum; SK, skin (incl. subcutaneous tissue); BL, Blood; BM, Bone marrow (from femur).

**Table S7. AAV2/8.TBG.*hARSB* biodistribution in female mice.**

| Organ | D15<br>AAV DNA | >LOQ (n) | <LOQ and >LOD (n) | <LOD (n) | D180<br>AAV DNA | > LOQ (n) | <LOQ and >LOD (n) | <LOD (n) |
|---|---|---|---|---|---|---|---|---|
| **LI** | 7.77x10$^{5}$±2.06x10$^{5}$ | 5 | 0 | 0 | 2.89x10$^{5}$±9.07x10$^{4**}$ | 5 | 0 | 0 |
| **GBL** | 4.08x10$^{4}$±4.13x10$^{4}$ | 5 | 0 | 0 | 3.13x10$^{4}$±2.34x10$^{4}$ | 5 | 0 | 0 |
| **AD** | 2.92x10$^{4}$±1.00x10$^{4}$ | 5 | 0 | 0 | 4.59x10$^{2}$±2.64x10$^{2**}$ | 5 | 0 | 0 |
| **ILN** | 8.26x10$^{3}$±4.85x10$^{3}$ | 5 | 0 | 0 | 1.36x10$^{3}$±8.73x10$^{2**}$ | 5 | 0 | 0 |
| **AO** | 7.84x10$^{3}$±6.64x10$^{3}$ | 5 | 0 | 0 | 9.48x10$^{2}$±4.88x10$^{2**}$ | 5 | 0 | 0 |
| **BL** | 7.37x10$^{3}$±3.80x10$^{3}$ | 5 | 0 | 0 | <LOD$^{**}$ | 0 | 0 | 5 |
| **KI** | 3.92x10$^{3}$±2.42x10$^{3}$ | 5 | 0 | 0 | 2.10x10$^{2}$±8.62x10$^{1**}$ | 5 | 0 | 0 |
| **SP** | 3.48x10$^{3}$±3.61x10$^{3}$ | 5 | 0 | 0 | 5.12x10$^{1**}$ | 1 | 4 | 0 |
| **UTE** | 3.36x10$^{3}$±1.28x10$^{3}$ | 5 | 0 | 0 | 1.29x10$^{2}$±7.69x10$^{1**}$ | 3 | 2 | 0 |
| **RE** | 3.22x10$^{3}$±1.96x10$^{3}$ | 5 | 0 | 0 | 3.60x10$^{2}$±2.09x10$^{2**}$ | 5 | 0 | 0 |
| **HE** | 3.21x10$^{3}$±1.30x10$^{3}$ | 5 | 0 | 0 | 2.38x10$^{2}$±1.42x10$^{2**}$ | 5 | 0 | 0 |
| **VAG** | 3.12x10$^{3}$±1.64x10$^{3}$ | 5 | 0 | 0 | 2.49x10$^{2}$±1.32x10$^{2**}$ | 4 | 0 | 0 |
| **SM** | 2.97x10$^{3}$±1.79x10$^{3}$ | 5 | 0 | 0 | 1.14x10$^{2}$±5.98x10$^{1**}$ | 5 | 0 | 0 |
| **SK** | 2.87x10$^{3}$±1.56x10$^{3}$ | 5 | 0 | 0 | 1.26x10$^{2}$±5.29x10$^{1**}$ | 3 | 2 | 0 |
| **MAM** | 2.68x10$^{3}$±7.06x10$^{2}$ | 5 | 0 | 0 | 1.02x10$^{2}$±6.44x10$^{1**}$ | 3 | 2 | 0 |
| **LOV** | 2.11x10$^{3}$±8.73x10$^{2}$ | 5 | 0 | 0 | 1.80x10$^{2**}$ | 1 | 3 | 1 |
| **STR** | 2.11x10$^{3}$±2.68x10$^{3}$ | 5 | 0 | 0 | 3.87x10$^{2}$±4.33x10$^{2*}$ | 5 | 0 | 0 |
| **GLA** | 2.09x10$^{3}$±3.29x10$^{3}$ | 5 | 0 | 0 | 5.90x10$^{2}$±1.00x10$^{3*}$ | 5 | 0 | 0 |
| **FOR** | 1.61x10$^{3}$±6.93x10$^{2}$ | 5 | 0 | 0 | 2.34x10$^{2}$±1.86x10$^{2**}$ | 5 | 0 | 0 |
| **MLN** | 1.36x10$^{3}$±1.55x10$^{3}$ | 5 | 0 | 0 | 1.10x10$^{2}$±4.40x10$^{1**}$ | 5 | 0 | 0 |
| **GTHY** | 1.34x10$^{3}$±3.94x10$^{3}$ | 5 | 0 | 0 | 1.46x10$^{2}$±6.09x10$^{1**}$ | 4 | 1 | 0 |
| **TRA** | 1.32x10$^{3}$±4.18x10$^{2}$ | 5 | 0 | 0 | 2.49x10$^{2}$±8.87x10$^{1**}$ | 5 | 0 | 0 |
| **OES** | 1.29x10$^{3}$±2.49x10$^{2}$ | 5 | 0 | 0 | 2.78x10$^{2}$±1.26x10$^{2**}$ | 5 | 0 | 0 |
| **PIT** | 1.26x10$^{3}$±6.11x10$^{2}$ | 5 | 0 | 0 | 1.08x10$^{2}$±5.50x10$^{1**}$ | 4 | 1 | 0 |
| **DU** | 9.04x10$^{2}$±2.16x10$^{2}$ | 5 | 0 | 0 | 1.27x10$^{2}$±6.26x10$^{1**}$ | 4 | 1 | 0 |
| **LU** | 8.49x10$^{2}$±5.15x10$^{2}$ | 5 | 0 | 0 | <LOQ$^{**}$ | 0 | 5 | 0 |
| **JE** | 8.21x10$^{2}$±5.11x10$^{1}$ | 5 | 0 | 0 | 9.89x10$^{1}$±3.67x10$^{1**}$ | 5 | 0 | 0 |
| **IL** | 7.37x10$^{2}$±1.42x10$^{2}$ | 5 | 0 | 0 | 7.39x10$^{1}$±8.79x10$^{0**}$ | 3 | 2 | 0 |
| **PAN** | 7.11x10$^{2}$±3.09x10$^{2}$ | 5 | 0 | 0 | 6.70x10$^{1**}$ | 1 | 2 | 2 |
| **URB** | 5.31x10$^{2}$±9.86x10$^{1}$ | 5 | 0 | 0 | 6.71x10$^{3}$ | 1 | 4 | 0 |
| **PNE** | 4.89x10$^{2}$±1.34x10$^{2}$ | 5 | 0 | 0 | 6.96x10$^{1**}$ | 1 | 2 | 2 |
| **CC** | 4.57x10$^{2}$±9.80x10$^{1}$ | 5 | 0 | 0 | 6.36x10$^{1}$±4.96x10$^{0**}$ | 2 | 3 | 0 |
| **SA** | 3.59x10$^{2}$±3.61x10$^{1}$ | 5 | 0 | 0 | 8.94x10$^{1}$±4.12x10$^{1**}$ | 2 | 2 | 1 |
| **CO** | 3.56x10$^{2}$±6.95x10$^{1}$ | 5 | 0 | 0 | 7.73x10$^{1}$±1.36x10$^{1**}$ | 3 | 2 | 0 |
| **ENC** | 2.34x10$^{2}$±3.40x10$^{1}$ | 5 | 0 | 0 | <LOQ$^{**}$ | 0 | 2 | 3 |
| **LAC** | 2.17x10$^{2}$±7.50x10$^{1}$ | 5 | 0 | 0 | <LOQ$^{**}$ | 0 | 3 | 2 |
| **STE** | 2.15x10$^{2}$±9.07x10$^{1}$ | 5 | 0 | 0 | <LOQ$^{**}$ | 0 | 2 | 3 |
| **BM** | 1.30x10$^{2}$±4.25x10$^{1}$ | 5 | 0 | 0 | <LOQ$^{**}$ | 0 | 3 | 2 |
| **LSC** | 1.26x10$^{2}$±3.88x10$^{1}$ | 4 | 1 | 0 | <LOD$^{**}$ | 0 | 0 | 5 |
| **EY** | 1.20x10$^{2}$±4.56x10$^{1}$ | 5 | 0 | 0 | <LOD$^{**}$ | 0 | 0 | 5 |
| **CSC** | 1.15x10$^{2}$±2.66x10$^{1}$ | 5 | 0 | 0 | <LOQ$^{**}$ | 0 | 1 | 3 |
| **TH** | 1.01x10$^{2}$±3.45x10$^{1}$ | 3 | 2 | 0 | <LOQ$^{*}$ | 0 | 3 | 2 |
| **CER** | 6.33x10$^{1}$±4.26x10$^{0}$ | 2 | 3 | 0 | <LOD$^{**}$ | 0 | 0 | 5 |

AAV2/8.TBG.*hARSB* (AAV) DNA is expressed as genome copies (GC) per μg of total DNA (GC/μg of total DNA). Results are reported as mean ± SD of > LOQ values and in a decreasing order on D15. The number (n) of animals analyzed per each time point is 5, except for *CSC* and *VAG* in females on D180 (n=4). The comparison between AAV DNA on D15 vs D180 was performed using the one sided Wilcoxon-Mann-Whitney test, assuming LOQ and LOD values for analysis. The *p*-value vs. D15 is: * <0.05 and ** < 0.01. Samples below the limit of quantification (LOQ) of the assay are reported as <LOQ. The LOQ is 50 GC/μg of total DNA except for: *CSL* (LOQ is 100 GC/μg of total DNA in 1 out 5 animals on D15); *PNE* (LOQ is 53 GC/μg of total DNA in 1 out 5 animals on D15). Samples below the limit of detection (LOD) of the assay are reported as <LOD. The LOD is 15 GC/μg of total DNA except for: *CSC* (LOD is 48 GC/μg of total DNA in out of 5. 75 GC/μg of total DNA in 1 out 5 and 29 GC/μg of total DNA in 1 out 5 animals on D180); *PNE* (LOD is 21 GC/μg of total DNA in out of 5). LOQ and LOD were recalculated based on the amount of DNA analyzed. i.e. when lower than 600 ng. *Abbreviations*: AAV, Adeno-associated viral vector; D, study day; LI, Liver; GBL, Gall bladder; SA, Salivary gland (sublingual); OES, Oesophagus; FOR, Forestomach; GLA, Glandular stomach; DU, Duodenum; JE, Jejunum; IL, Ileum incl. Peyer's patches; CC, Caecum; CO, Colon; RE, Rectum; PAN, Pancreas; TH, Thymus; SP, Spleen; MLN, Mesenteric lymph node; ILN, Inguinal lymph node; KI, Kidney; URB, Urinary bladder; OV, Ovaries (with

Fallopian tubes); VAG, Vagina; UTE, Uterus (with uterine cervix); GTHY, Thyroids with parathyroids; AD, Adrenal; PIT, Pituitary; HE, Heart; AO, Aorta; TRA, Trachea; LU, Lungs (incl. bronchi and bronchioles); ENC, brain (encephalus); CER, brain (cerebellum); STE, brain stem; CSC, cervical spinal cord; LSC, lumbar spinal cord; PNE, peripheral nerve (sciatic); EY, Eye; LAC, lachrymal gland (harderian gland); SM, skeletal muscle; STR, sternum; SK, skin (incl. subcutaneous tissue); MAM, mammary gland; BL, Blood; BM, Bone marrow (from femur).

## Table S8. AAV2/8.TBG.*hARSB* shedding in mice

| | Males | | | | | Females | | | | |
|---|---|---|---|---|---|---|---|---|---|---|
| **Time points** | **n** | **AAV DNA** | **>LOQ (n)** | **<LOQ and >LOD (n)** | **<LOD (n)** | **n** | **AAV DNA** | **> LOQ (n)** | **<LOQ and >LOD (n)** | **<LOD (n)** |
| **STOOL** | | | | | | | | | | |
| **D2** | 11 | $1.25x10^{5}\pm1.43x10^{5}$ | 11 | 0 | 0 | 16 | $6.70x10^{4}\pm9.13x10^{4}$ | 16 | 0 | 0 |
| **D4** | 11 | $8.77x10^{3}\pm3.66x10^{3\,**}$ | 11 | 0 | 0 | 16 | $1.26x10^{4}\pm7.77x10^{3**}$ | 16 | 0 | 0 |
| **D11** | 11 | $6.10x10^{1}\pm5.66x10^{0\,**}$ | 2 | 3 | 6 | 16 | $7.90x10^{1**}$ | 1 | 1 | 14 |
| **D14** | 10 | $4.70x10^{2\,**}$ | 1 | 1 | 8 | 13 | <LOD$^{**}$ | 0 | 0 | 13 |
| **D22** | 7 | <LOD$^{**}$ | 0 | 0 | 7 | 11 | <LOD$^{**}$ | 0 | 0 | 11 |
| **URINE** | | | | | | | | | | |
| **D2** | 5 | $2.26x10^{2}\pm1.75x10^{1}$ | 2 | 1 | 2 | 6 | $3.70x10^{2}\pm1.25x10^{2}$ | 3 | 2 | 1 |
| **D4** | 6 | <LOD | 0 | 0 | 6 | 6 | < LOQ | 0 | 1 | 5 |
| **D11** | 6 | <LOD | 0 | 0 | 6 | 6 | $2.90x10^{2}$ | 1 | 0 | 5 |
| **D14** | 6 | <LOD | 0 | 0 | 6 | 6 | <LOD | 0 | 0 | 6 |
| **D22** | 4 | <LOD | 0 | 0 | 4 | 4 | <LOQ | 0 | 1 | 3 |
| **D37** | - | - | - | - | - | 2 | <LOQ | 0 | 1 | 1 |
| **D60** | - | - | - | - | - | 1 | <LOD | 0 | 0 | 1 |
| **PLASMA** | | | | | | | | | | |
| **D2** | 7 | $1.28x10^{7}\pm1.22x10^{6}$ | 7 | 0 | 0 | 8 | $1.90x10^{7}\pm5.14x10^{6}$ | 8 | 0 | 0 |
| **D9** | 8 | $1.02x10^{3}\pm6.38x10^{2**}$ | 7 | 1 | 0 | 8 | $8.47x10^{2}\pm7.39x10^{2**}$ | 3 | 5 | 0 |
| **D15** | 4 | $3.38x10^{2}\pm2.06x10^{2**}$ | 4 | 0 | 0 | 5 | $4.05x10^{2}\pm1.81x10^{2**}$ | 4 | 1 | 0 |
| **D23** | 6 | <LOQ$^{**}$ | 0 | 5 | 1 | 6 | <LOQ$^{**}$ | 0 | 6 | 0 |
| **D37** | 6 | <LOQ | 0 | 1 | 5 | 6 | <LOD$^{**}$ | 0 | 0 | 6 |
| **D60** | 1 | <LOD | 0 | 0 | 1 | - | - | - | - | - |

AAV2/8.TBG.*hARSB* (AAV) DNA is expressed as genome copies per µg of total DNA (GC/µg of total DNA) for stool and GC per 100 µl of fluid (GC/100 µl) for urine and plasma. Results are reported as mean ± SD of > LOQ values. The limit of quantification (LOQ) and detection (LOD) of the assay are 50 GC/µg of total DNA and 15 GC/µg of total DNA for stool and 30 GC/well and 9 GC/well for urine and plasma. LOQ and LOD were recalculated based on the amount of DNA or the volume of sample analyzed. The comparison of AAV-DNA concentrations over time was performed, in males and females, by means of an ANOVA for repeated measurements. As the time effect was found statistically significant (p-value<0.01), the post-hoc paired comparison vs D2 were done with the Dunnett test. The *p*-value is: * <0.05 and ** < 0.01.*Abbreviations*: AAV, Adeno-associated viral vector; D, study day; - , not available.

**Table S9. AAV2/8.TBG.*hARSB* expression in male mice**

| Organ | D15 AAV RNA | >LOQ (n) | <LOQ and >LOD (n) | <LOD (n) | D180 AAV RNA | > LOQ (n) | <LOQ and >LOD (n) | <LOD (n) |
|---|---|---|---|---|---|---|---|---|
| **LI** | 2.05x10$^{7}$±2.42x10$^{6}$ | 5 | 0 | 0 | 5.64x10$^{6}$±2.14 x10$^{6**}$ | 5 | 0 | 0 |
| **GBL** | 8.76x10$^{6}$±3.73x10$^{6}$ | 5 | 0 | 0 | 2.53x10$^{6}$±9.56 x10$^{5**}$ | 5 | 0 | 0 |
| **CSC** | 4.27x10$^{5}$±6.47x10$^{5}$ | 3 | 0 | 2 | <LOD | 0 | 0 | 3 |
| **GLA** | 9.94x10$^{4}$±1.85x10$^{5}$ | 4 | 1 | 0 | 2.95x10$^{3}$ | 1 | 2 | 2 |
| **AD** | 5.50x10$^{4}$±5.73x10$^{4}$ | 2 | 0 | 3 | 6.72x10$^{4}$±3.79x10$^{4}$ | 5 | 0 | 0 |
| **TRA** | 2.07x10$^{4}$±2.82x10$^{4}$ | 3 | 2 | 0 | 3.21x10$^{3}$±3.26 x10$^{3}$ | 2 | 1 | 2 |
| **PNE** | 1.47x10$^{4}$ | 1 | 0 | 4 | <LOD | 0 | 0 | 5 |
| **EY** | 1.12x10$^{4}$ | 1 | 0 | 4 | <LOQ | 0 | 1 | 2 |
| **DU** | 1.06x10$^{4}$±1.16x10$^{4}$ | 2 | 0 | 3 | 1.15x10$^{3}$ | 1 | 1 | 3 |
| **FOR** | 9.54x10$^{3}$±9.78x10$^{3}$ | 5 | 0 | 0 | <LOQ$^{*}$ | 0 | 3 | 2 |
| **STE** | 9.34x10$^{3}$±5.98x10$^{2}$ | 2 | 1 | 2 | <LOD | 0 | 0 | 3 |
| **TH** | 7.77x10$^{3}$±6.87x10$^{3}$ | 2 | 2 | 1 | 5.17x10$^{3}$±5.80 x10$^{3}$ | 4 | 0 | 1 |
| **ENC** | 6.57x10$^{3}$±2.04x10$^{3}$ | 2 | 0 | 3 | <LOQ | 0 | 1 | 2 |
| **ILN** | 3.86x10$^{3}$±1.59x10$^{3}$ | 4 | 0 | 1 | 7.83x10$^{3}$±7.34 x10$^{3}$ | 3 | 2 | 0 |
| **SP** | 3.36x10$^{3}$ | 1 | 2 | 2 | <LOQ | 0 | 1 | 4 |
| **HE** | 2.52x10$^{3}$±3.12x10$^{1}$ | 2 | 0 | 3 | <LOQ | 0 | 1 | 2 |
| **PIT** | 2.06x10$^{3}$ | 1 | 0 | 4 | <LOQ | 0 | 1 | 4 |
| **CC** | 1.09x10$^{3}$ | 1 | 0 | 4 | <LOD | 0 | 0 | 3 |
| **STR** | 9.02x10$^{2}$±2.65x10$^{2}$ | 2 | 2 | 1 | 1.07x10$^{3}$ | 1 | 2 | 2 |
| **URB** | 8.90x10$^{2}$ | 1 | 1 | 3 | <LOD | 0 | 0 | 3 |
| **OES** | 8.06x10$^{2}$ | 1 | 1 | 3 | 1.00x10$^{4}$ | 1 | 2 | 2 |
| **LU** | 7.37x10$^{2}$ | 1 | 1 | 3 | 1.04x10$^{3}$ | 1 | 1 | 3 |
| **AO** | <LOQ | 0 | 4 | 1 | 1.36x10$^{3}$ | 1 | 4 | 0 |
| **PAN** | <LOQ | 0 | 3 | 0 | - | - | - | - |
| **CO** | <LOQ | 0 | 2 | 1 | - | - | - | - |
| **MLN** | <LOQ | 0 | 2 | 1 | - | - | - | - |
| **KI** | <LOQ | 0 | 1 | 2 | - | - | - | - |
| **LHEP** | <LOQ | 0 | 1 | 2 | - | - | - | - |
| **LTEP** | <LOQ | 0 | 1 | 2 | - | - | - | - |
| **GTHY** | <LOQ | 0 | 1 | 4 | - | - | - | - |
| **SM** | <LOQ | 0 | 1 | 2 | - | - | - | - |
| **LAC** | <LOD | 0 | 0 | 5 | <LOQ | 0 | 1 | 2 |
| **RE** | <LOD | 0 | 0 | 3 | - | - | - | - |
| **SA** | <LOD | 0 | 0 | 3 | - | - | - | - |
| **JE** | <LOD | 0 | 0 | 5 | <LOD | 0 | 0 | 3 |
| **IL** | <LOD | 0 | 0 | 3 | - | - | - | - |
| **LTE** | <LOD | 0 | 0 | 3 | - | - | - | - |
| **RTE** | <LOD | 0 | 0 | 3 | - | - | - | - |
| **PRO** | <LOD | 0 | 0 | 3 | - | - | - | - |
| **SV** | <LOD | 0 | 0 | 3 | - | - | - | - |
| **RHEP** | <LOD | 0 | 0 | 3 | - | - | - | - |
| **RTEP** | <LOD | 0 | 0 | 3 | - | - | - | - |
| **CER** | <LOD | 0 | 0 | 3 | - | - | - | - |
| **LCS** | <LOD | 0 | 0 | 5 | - | - | - | - |
| **SK** | <LOD | 0 | 0 | 5 | - | - | - | - |
| **BL** | <LOD | 0 | 0 | 5 | - | - | - | - |
| **BM** | <LOD | 0 | 0 | 5 | - | - | - | - |

Expression of *hARSB* from AAV2/8.TBG.*hARSB* (AAV RNA) is reported as RNA copy number per µg of total RNA (RNA copy number/µg of total RNA). Results are reported as mean ± SD of > LOQ values and in a decreasing order based on results obtained on D15. The number (n) of animals analyzed per each time point was defined according to the scheme reported in the Material and Methods section: n= 5 for LI, GBL, OES, FOR, GLA, DU, JE, CC, TH, SP, ILN, URB, GTHY, AD, PIT, HE, AO, TRA, LU, ENC, STE, PNE, LAC, CSC, LSC, EY, SK, STR, BL, BM and n=3 in remaining organs on D15; n= 5 in LI, GBL, OES, FOR, GLA, DU, TH, SP, ILN, AO, TRA, LU, PNE and n=3 in JE, CC, URB, HE, ENC, STE, LSC, CSC, EYE, while the remaining organs were not analyzed on D180; The comparison between AAV RNA on D15 vs D180 was performed using the one-sided Wilcoxon-Mann-Whitney test, assuming LOQ and LOD values for analysis. The *p*-value vs. D15 is: ** < 0.01. Samples below the limit of quantification (LOQ) of the assay are reported as <LOQ. The LOQ is 625 RNA copy number /µg of total RNA. Samples below the limit of detection (LOD) of the assay are reported as <LOD. The LOD is 187.5 RNA copy number/µg of

total RNA. LOQ and LOD were recalculated based on the amount of RNA analyzed. i.e. when lower than 600 ng. *Abbreviations*: AAV, Adeno-associated viral vector; D, study day; LI, Liver; GBL, Gall bladder; SA, Salivary gland (sublingual); OES, Oesophagus; FOR, Forestomach; GLA, Glandular stomach; DU, Duodenum; JE, Jejunum; IL, Ileum incl. Peyer's patches; CC, Caecum; CO, Colon; RE, Rectum;, PAN, Pancreas; TH, Thymus; SP, Spleen; MLN, Mesenteric lymph node; ILN, Inguinal lymph node; KI, Kidney; URB, Urinary bladder;; TE, Testes; HEP, Head of epididymis; TEP, Tail of epididymis; SV, Seminal vesicles (with coagulating gland); PRO, Prostate; GTHY, Thyroids with parathyroids; AD, Adrenal; PIT, Pituitary; HE, Heart; AO, Aorta; TRA, Trachea; LU, Lungs (incl. bronchi and bronchioles); ENC, brain (encephalus); CER, brain (cerebellum); STE, brain stem; CSC, cervical spinal cord; LSC, lumbar spinal cord; PNE, peripheral nerve (sciatic); EY, Eye; LAC, lachrymal gland (harderian gland); SM, skeletal muscle; STR, sternum; SK, skin (incl. subcutaneous tissue); BL, Blood; BM, Bone marrow (from femur); - not analyzed on D180.

**Table S10. AAV2/8.TBG.*hARSB* expression in female mice**

| Organ | D15 | | | | D180 | | | |
|---|---|---|---|---|---|---|---|---|
| | AAV RNA | >LOQ (n) | <LOQ and >LOD (n) | <LOD (n) | AAV RNA | >LOQ (n) | <LOQ and >LOD (n) | <LOD |
| LI | $7.73x10^6±1.81x10^6$ | 5 | 0 | 0 | $3.15x10^6±2.18x10^{6**}$ | 5 | 0 | 0 |
| GBL | $2.68x10^6±1.59x10^6$ | 5 | 0 | 0 | $1.07x10^6±8.92x10^5$ | 5 | 0 | 0 |
| CSC | $2.78x10^4±2.87x10^4$ | 3 | 0 | 2 | <LOD | 0 | 0 | 3 |
| GLA | $2.39x10^4±4.29x10^4$ | 5 | 0 | 0 | $2.75x10^4±3.35x10^4$ | 3 | 0 | 2 |
| FOR | $6.63x10^3±5.91x10^3$ | 4 | 1 | 0 | $9.84x10^2±3.83x10^2$ | 3 | 0 | 2 |
| LU | $2.78x10^3±9.97x10^2$ | 3 | 1 | 1 | <LOQ | 0 | 2 | 3 |
| SP | $2.10x10^3±3.44x10^2$ | 3 | 2 | 0 | $7.36x10^2$ | 1 | 0 | 4 |
| TRA | $2.02x10^3±1.10x10^3$ | 3 | 0 | 2 | $8.30x10^{2*}$ | 1 | 4 | 0 |
| STE | $1.61x10^3$ | 1 | 1 | 3 | <LOD | 0 | 0 | 3 |
| ILN | $1.51x10^3$ | 1 | 1 | 3 | $3.57x10^3±1.80x10^2$ | 5 | 0 | 0 |
| AO | $1.13x10^3±4.93x10^2$ | 3 | 1 | 1 | $1.41x10^3±4.78x10^2$ | 3 | 1 | 1 |
| STR | $1.09x10^3±3.47x10^2$ | 2 | 2 | 1 | $1.31x10^3±4.72x10^2$ | 2 | 0 | 3 |
| LAC | $1.06x10^3$ | 1 | 1 | 3 | <LOD | 0 | 0 | 3 |
| JE | $1.02x10^3$ | 1 | 1 | 3 | <LOQ | 0 | 1 | 2 |
| ENC | $9.99x10^2$ | 1 | 1 | 3 | <LOD | 0 | 0 | 3 |
| DU | <LOQ | 0 | 2 | 3 | <LOD | 0 | 0 | 5 |
| CO | <LOQ | 0 | 2 | 1 | - | - | - | - |
| MLN | <LOQ | 0 | 2 | 1 | - | - | - | - |
| HE | <LOQ | 0 | 2 | 3 | <LOQ | 0 | 1 | 2 |
| SA | <LOQ | 0 | 1 | 2 | - | - | - | - |
| EY | <LOQ | 0 | 1 | 4 | <LOD | 0 | 0 | 3 |
| MAM | <LOQ | 0 | 1 | 4 | - | - | - | - |
| TH | <LOD | 0 | 0 | 5 | $1.55x10^3±4.10x10^2$ | 2 | 1 | 2 |
| OES | <LOD | 0 | 0 | 5 | <LOQ | 0 | 2 | 3 |
| PIT | <LOD | 0 | 0 | 5 | <LOQ | 0 | 1 | 2 |
| RE | <LOD | 0 | 0 | 3 | - | - | - | - |
| IL | <LOD | 0 | 0 | 3 | - | - | - | - |
| CC | <LOD | 0 | 0 | 5 | <LOD | 0 | 0 | 3 |
| PAN | <LOD | 0 | 0 | 3 | - | - | - | - |
| URB | <LOD | 0 | 0 | 5 | <LOD | 0 | 0 | 3 |
| KI | <LOD | 0 | 0 | 3 | - | - | - | - |
| LOV | <LOD | 0 | 0 | 3 | - | - | - | - |
| ROV | <LOD | 0 | 0 | 3 | - | - | - | - |
| VAG | <LOD | 0 | 0 | 3 | - | - | - | - |
| UTE | <LOD | 0 | 0 | 3 | - | - | - | - |
| GTHY | <LOD | 0 | 0 | 4 | - | - | - | - |
| AD | <LOD | 0 | 0 | 3 | - | - | - | - |
| CER | <LOD | 0 | 0 | 3 | - | - | - | - |
| LCS | <LOD | 0 | 0 | 5 | <LOD | 0 | 0 | 3 |
| PNE | <LOD | 0 | 0 | 3 | - | - | - | - |
| SK | <LOD | 0 | 0 | 3 | - | - | - | - |
| SM | <LOD | 0 | 0 | 3 | - | - | - | - |
| BL | <LOD | 0 | 0 | 5 | - | - | - | - |
| BM | <LOD | 0 | 0 | 5 | - | - | - | - |

Expression of *hARSB* from AAV2/8.TBG.*hARSB* (AAV RNA) is reported as RNA copy number per µg of total RNA (RNA copy number/µg of total RNA). Results are reported as mean ± SD of > LOQ values and in a decreasing order based on results obtained on D15. The number (n) of animals analyzed per each time point was defined according to the scheme reported in the Material and Methods section: n=5 for LI, GBL, OES, FOR, GLA, DU, JE, CC, TH, SP, ILN, URB, AD, PIT, HE, AO, TRA, LU, ENC, STE, PNE, LAC, CSC, EY, MAM, STR, BL, BM, n=4 for GTHY (one animal is missing because sample was not collected) and n=3 in the remaining organs on D15; n=5 for LI, GBL, OES, FOR, GLA, DU, TH, SP, ILN, AO, TRA, LU and n=3 in JE, CC, URB, PIT, HE, ENC, STE, PNE, LAC, CSC, EYE, while the remaining organs were not analyzed_on D180. The comparison between AAV RNA on D15 vs D180 was performed using the one-sided Wilcoxon-Mann-Whitney test, assuming LOQ and LOD values for analysis. The *p*-value vs. D15 is: ** < 0.01. Samples below the limit of quantification (LOQ) of the assay are reported as <LOQ. The LOQ is 625 RNA copy number /µg of total RNA. Samples below the limit of detection (LOD) of the assay are reported as <LOD. The LOD is 187.5 RNA copy number/µg of total RNA. LOQ and LOD were recalculated based on the amount of RNA analyzed. i.e. when lower than 600 ng. *Abbreviations*: AAV, Adeno-associated viral vector; D, study day; LI, Liver; GBL, Gall bladder; SA, Salivary gland (sublingual); OES, Oesophagus; FOR, Forestomach; GLA, Glandular stomach; DU, Duodenum; JE, Jejunum; IL, Ileum

incl. Peyer's patches; CC, Caecum; CO, Colon; RE, Rectum;, PAN, Pancreas; TH, Thymus; SP, Spleen; MLN, Mesenteric lymph node; ILN, Inguinal lymph node; KI, Kidney; URB, Urinary bladder; OV, Ovaries (with Fallopian tubes); VAG, Vagina; UTE, Uterus (with uterine cervix); GTHY, Thyroids with parathyroids; AD, Adrenal; PIT, Pituitary; HE, Heart; AO, Aorta; TRA, Trachea; LU, Lungs (incl. bronchi and bronchioles); ENC, brain (encephalus); CER, brain (cerebellum); STE, brain stem; CSC, cervical spinal cord; LSC, lumbar spinal cord; PNE, peripheral nerve (sciatic); EY, Eye; LAC, lachrymal gland (harderian gland); SM, skeletal muscle; STR, sternum; SK, skin (incl. subcutaneous tissue); BL, Blood; BM, Bone marrow (from femur); - not analyzed on D180.

**Table S11. Phenotype improvement and liver genome copies in transgenic MPS VI mice treated with AAV2/8.TBG.*hARSB***

| Groups | Serum ARSB | Liver AAV GC | ARSB | GAGs | Kidney ARSB | GAGs | Spleen ARSB | GAGs |
|---|---|---|---|---|---|---|---|---|
| **NR** | 13335±1216$^{**}$ | - | 106.6±15.6 | 4.2±0.4$^{**}$ | 110.7±18.8$^{**}$ | 8.3±1.0$^{**}$ | 74.3±5.7$^{**}$ | 3.1±0.5$^{**}$ |
| **AF** | 0±0 | - | 0.0±0.0 | 46.4±5.8 | 0.0±0.0 | 55.3±7.2 | 0.0±0.0 | 35.2±2.1 |
| **AAV 2x10$^{11}$** | 119±93 | 0.018±0.004 | 8.2±2.6 | 2.9±1.0$^{**}$ | 1.9±0.4 | 27.4±5.2$^{**}$ | 6.7±0.1 | 5.6±1.7$^{**}$ |
| **AAV 6x10$^{11}$** | 1082±388 | 0.231±0.111 | 39.4±20.5 | 3.7±1.5$^{**}$ | 1.2±0.1 | 14.6±4.5$^{**}$ | 6.1±0.5 | 2.3±0.5$^{**}$ |
| **AAV 2x10$^{12}$** | 8714±2745$^{*}$ | 2.509±1.182 | 62.9±12.2 | 2.8±1.0$^{**}$ | 1.5±0.1 | 8.5±1.7$^{**}$ | 7.4±0.8 | 2.5±0.5$^{**}$ |

Serum ARSB is expressed as pg/ml. The average of the mean serum ARSB measured over time in each animal is reported per each group. The number (*n*) of animals is: NR, n=15; AF, n=4; AAV 2x10$^{11}$, n=4; AAV 6x10$^{11}$, n= 5; AAV 2x10$^{12}$, n= 5. AAV GC are expressed as genome copies/molecules of diploid genome. ARSB activity in tissues is expressed as nmol/mg protein/hour. Tissue GAG levels are expressed as μg/mg of protein. The number (*n*) of animals is: NR, n=14; AF, n=8; AAV 2x10$^{11}$, n=4; AAV 6x10$^{11}$, n= 4; AAV 2x10$^{12}$, n=5. All results are represented as mean ± SEM. The comparison between the 5 groups was performed using a one-way ANOVA. In case of a significant global ANOVA test, Tukey-*post hoc* test was used for paired comparisons. The *p*-value vs AF is: *<0.05 and ** ≤0.01. *Abbreviations:* AAV: Adeno-associated viral vector; GC: genome copies; NR: normal mice; AF: MPS VI affected mice; AAV 2x10$^{11}$: MPS VI affected mice which received 2x10$^{11}$ GC/kg AAV2/8.TBG.*hARSB*; AAV 6x10$^{11}$: MPS VI affected mice which received 6x10$^{11}$ GC/kg AAV2/8.TBG.*hARSB*; AAV 2x10$^{12}$: MPS VI affected mice which received 2x10$^{12}$ GC/kg AAV2/8.TBG.*hARSB*; -: below limit of quantification.
