# Supplementary material for: Non-clinical Safety and Efficacy of an AAV2/8 Vector Administered Intravenously for Treatment of Mucopolysaccharidosis Type VI
Source: Mol Ther Methods Clin Dev. 2017 Jul 24;6:143–58. doi: 10.1016/j.omtm.2017.07.004 (PMC5552066; doi:10.1016/j.omtm.2017.07.004)
Supplement: Document S2. Article plus Supplemental Information [file mmc2.pdf]

Molecular Therapy
Methods & Clinical Development
Original Article

Non-clinical Safety and Efficacy of an AAV2/8 Vector Administered Intravenously for Treatment of Mucopolysaccharidosis Type VI

Rita Ferla,[1,2] Marialuisa Alliegro,[1,2] Jean-Brice Marteau,[3] Margherita Dell'Anno,[1,2] Edoardo Nusco,[1] Severine Pouillot,[3] Stefania Galimberti,[4] Maria Grazia Valsecchi,[4] Vincent Zuliani,[3] and Alberto Auricchio[1,5]

[1]Telethon Institute of Genetics and Medicine (TIGEM), Pozzuoli (Naples) 80078, Italy; [2]Medical Genetics, Department of Translational Medicine, Federico II University, Naples 80131, Italy; [3]Genosafe, Evry 91000, France; [4]Center of Biostatistics for Clinical Epidemiology, School of Medicine and Surgery, University of Milano-Bicocca, Monza 20900, Italy; [5]Department of Advanced Biomedicine, Federico II University, Naples 80131, Italy

In vivo gene therapy with adeno-associated viral (AAV) vectors is safe and effective in humans. We recently demonstrated that AAV8-mediated liver gene transfer is effective in animal models of mucopolysaccharidosis type VI (MPS VI), a rare lysosomal storage disease that is caused by arylsulfatase B (ARSB) deficiency. In preparing for a first-in-human trial, we performed non-clinical studies to assess the safety of intravenous administrations of AAV2/8.TBG.*hARSB* produced under good manufacturing practice-like conditions. No toxicity was observed in AAV-treated mice, except for a transient increase in alanine aminotransferase in females and thyroid epithelial hypertrophy. AAV2/8.TBG.*hARSB* biodistribution and expression confirmed the liver as the main site of both infection and transduction. Shedding and breeding studies suggest that the risk of both horizontal and germline transmission is minimal. An AAV dose-response study in MPS VI mice was performed to define the range of doses to be used in the clinical study. Overall, these data support the non-clinical safety and efficacy of AAV2/8.TBG.*hARSB* and pave the way for a phase I/II clinical trial based on intravascular infusions of AAV8 in patients with MPS VI.

INTRODUCTION

Adeno-associated viral (AAV) vectors are widely used for in vivo gene therapy applications due to their excellent safety and efficacy profiles in both preclinical and clinical trials.[1–8]

We recently developed a gene therapy approach for mucopolysaccharidosis type VI (MPS VI).[9–15] MPS VI is a rare lysosomal storage disorder (LSD) that is caused by arylsulfatase B (ARSB) deficiency, which results in widespread accumulation and urinary excretion of toxic glycosaminoglycans (GAGs).[16] We demonstrated that a single systemic administration of a recombinant AAV vector serotype 8 (AAV2/8), which encodes ARSB under the transcriptional control of the liver-specific thyroxine-binding globulin (TBG) promoter (AAV2/8.TBG.*hARSB*), results in sustained liver transduction and phenotypic improvement in MPS VI animal models.[9,11–15] We also showed that this is at least as effective in MPS VI mice as weekly administrations of enzyme replacement therapy (ERT), which is the current standard of care for this condition.[17–19] These encouraging results support the development of a gene therapy clinical trial for MPS VI. The study was designed as a phase I/II open-label, dose-escalation study to test both the safety and efficacy of a single systemic administration of AAV2/8.TBG.*hARSB* at doses ranging between 2×10^{11} and 2×10^{12} genome copies (GC)/kg body weight.

In view of this, we performed investigational new drug (IND)-enabling non-clinical studies, which were predominantly good laboratory practice (GLP) compliant, to assess the safety and biodistribution of intravenous (i.v.) administrations of AAV2/8.TBG.*hARSB* in mice or wild-type (WT) rabbits. A dose-response study was also performed to establish the range of doses to be used in humans. These studies were conducted using a vector lot produced according to a process that is similar to the good manufacturing practice (GMP) process employed for the clinical lot. Briefly, production of this lot was performed in a GMP facility, using GMP-source-grade production plasmids derived from characterized plasmid cell banks.

Here, we demonstrate that systemic administration of GMP-like AAV2/8.TBG.*hARSB* is safe in animal models and shows similar efficacy to the research-grade vector used in previous proof-of-principle studies.[9,13]

RESULTS AND DISCUSSION

Production of AAV2/8.TBG.*hARSB* Vector by Polyethylenimine Transfection of HEK293T in Serum-free Media and AAV Purification from Medium

In preparation for a clinical trial testing the safety and efficacy of systemic administration of AAV2/8.TBG.*hARSB*, we performed

Received 4 May 2017; accepted 19 July 2017;
http://dx.doi.org/10.1016/j.omtm.2017.07.004.

Correspondence: Rita Ferla, Telethon Institute of Genetics and Medicine, Via Campi Flegrei 34, Pozzuoli (Naples) 80078, Italy.
E-mail: ferla@tigem.it
Correspondence: Alberto Auricchio, Telethon Institute of Genetics and Medicine, Via Campi Flegrei 34, Pozzuoli (Naples) 80078, Italy.
E-mail: auricchio@tigem.it

This is an open access article under the CC BY license (http://creativecommons.org/licenses/by/4.0/).

Table 1. Experimental Plan

	GLP				Non-GLP
Study	Toxicity	Biodistribution	Germline Transmission	Expression	Dose Response
Species	mouse	mouse	rabbit	mouse	mouse
Strain	C57/BL6-Tg*ARSBC91S*	C57/BL6-Tg*ARSBC91S*	New Zealand	C57/BL6-Tg*ARSBC91S*	MPS VI
AAV dose (GC/kg)	2×10^{13}	2×10^{12}	2×10^{12}	2×10^{12}	2×10^{11}, 6×10^{11}, 2×10^{12}
Time points	D15 and D180	D15 and D180	D150	D15 and D180	D150 and D180

AAV, AAV2/8.TBG.*hARSB*; D, study day; GC, genome copies; GLP, good laboratory practice; MPS VI, mucopolysaccharidosis type VI.

non-clinical studies required before first use in humans (Table 1). These were conducted using a vector lot produced according to a process that is similar to the GMP process employed for the clinical lot.

Taking advantage of the large amount of AAV2/8 vector found in the medium of producing cells and the higher purity of the medium than the cell-derived material as the source of AAV2/8, the AAV2/8.TBG.*hARSB* vector was purified from the supernatant of transfected cells.[20] Polyethylenimine (PEI) was used to transfect derivative HEK293T cells under serum-free conditions. Virus was concentrated by tangential flow filtration (TFF) from the harvested media. Vector genome-containing particles were purified from both empty capsids and process residuals by iodixanol gradient ultracentrifugation.

As mentioned above, this lot was used both in efficacy dose-response and non-clinical safety, biodistribution, and expression studies. Table 2 reports the quality control performed on this lot.

Systemic Infusion of AAV2/8.TBG.*hARSB* Does Not Result in Detectable Toxicity in Mice

A safety study was performed to determine potential toxic risks associated with ARSB overexpression derived from liver-directed AAV2/8.TBG.*hARSB* and with the quality, formulation, and administration of the vector preparation (Table 1).

To avoid confounding interspecies immune responses to ARSB (i.e., those that may occur when expressing human ARSB in a mouse), adult heterozygous C57/BL6-Tg*ARSBC91S* mice were generated that harbor the *hARSB* open reading frame (ORF) with the C91S point mutation in the gt(ROSA)26sor.[21,22] The C91S mutation inactivates the ARSB enzymatic activity, preserving the protein conformation and thus making this mouse immune tolerant to WT hARSB.

We selected study days 15 (D15) and 180 (D180) post-injection as time points to evaluate the safety of AAV2/8.TBG.*hARSB* administration, because serum ARSB peaks 15 days following AAV2/8.TBG.*hARSB* vector injection and remains stable at least up to 180 days.[13] Thus, we evaluated the impact of AAV2/8.TBG.*hARSB* administration on hematology, clinical chemistry, necropsy, and histopathology at both D15 and D180. Observations of general health, including a physical, functional, and neurobehavioral examination and assessments of body weight, body temperature, and food intake, were made throughout the study.

C57/BL6-Tg*ARSBC91S* mice received a single systemic administration of either 2×10^{13} GC/kg (10× the highest dose proposed for the clinical trial) of the test item (i.e. AAV2/8.TBG.hARSB diluted in 0.9% NaCl saline solution) or the excipient, which served as the control. Mice were injected after post-natal day 60 (p60), when vector dilution due to hepatocyte proliferation was minimal, based on previous observations by us and others.[10,13,23]

Mortality, clinical alterations, and changes in body temperature and food intake were not observed during the study (data not shown). There was no significant change in body weight gain of animals treated with the test item compared to controls (Figure S1). No major evidence of toxicity was observed in the hematology and blood chemistry profiles of mice treated with the test item compared to the control: small differences were seen in some parameters that, although statistically significant, were of no clinical/toxicological relevance (Figure 1; Tables S1 and S2). A statistically significant increase in alanine aminotransferase (ALT), a marker of liver function, was reported on D15 in females treated with the test item compared to the control but disappeared in the long-term observation on D180 (Figure 1A; Table S2). No significant changes in other parameters related to liver function were measured (Figure 1A; Table S2). As no signs of liver damage were seen at necropsy, the increase in ALT is likely not toxicologically relevant. Noticeably, the clinical trial protocol includes strict monitoring of ALT and aspartate aminotransferase (AST) in patients with MPS VI who are administered the vector. Indeed, subjects that receive an intravascular injection of either AAV2/2 or AAV2/8 are at risk of developing a subclinical, transient increase in AST and ALT due to a cell-mediated immune response to the transduced hepatocytes, which has not been reported in animal models.[2,4,24,25] This reaction, which was observed in patients that received 2×10^{12} GC/kg vector and occurred between 6 and 9 weeks post-AAV injection, can be blunted with a prompt oral treatment with prednisolone.[2,4] Therefore, in the proposed clinical trial, AST and ALT will be closely monitored after vector administration to promptly identify any signs of liver toxicity.

At euthanization, no variations in body and organ weight were considered to be toxicologically relevant or attributable to an effect of the test item in either male or female mice. These variations were either of low amplitude or did not correlate with the microscopic findings (data not shown). Similarly, the macroscopic abnormalities observed at necropsy either did not correlate with the

Table 2. Purity, Potency, and Residual Contaminants of the AAV2/8.TBG.*hARSB* Vector Lot

Test	Method	Results
Purity	SDS-PAGE/ densitometry	93% intensity for AAV VPI, VPII, and VPIII
Infectious titer	$TCID_{50}$/qPCR	8×10^7 $TCDI_{50}$/mL
Host cell protein	ELISA	232 ng/mL
Host cell DNA	qPCR	262 ng/mL
Adenovirus protein	western blot	<9.8 ng/mL
Empty particles	electron microscopy	<20% empty particles
Replication competent AAV	infectivity/qPCR	<1 rcAAV/10^7 rAAV
SV40 DNA	qPCR	284 copies/μL (360 bp amplicon)
Endotoxin	LAL assay	<0.5 EU/mL
Sterility	direct inoculation	no growth in test media

AAV, adeno-associated virus; LAL, limulus amebocyte lysate; rcAAV, replication competent AAV; $TCDI_{50,}$ tissue culture infective dose 50; VP, viral protein.

microscopic abnormalities or their incidence was not remarkable (Table S3).

The histopathology examination revealed no treatment-related changes in the organs analyzed, except for the thyroid. Indeed, hypertrophy of the thyroid follicular epithelium was more frequent and severe in the group administered the test item than in the control group (Table S4). These changes are commonly associated with long-term stimulation of the thyroid, possibly by a lowering of circulating thyroid hormones. Since no serum was left to confirm this by dosing thyroid hormones, we considered indirect signs (e.g., serum levels of cholesterol, triglycerides, glucose, mean platelet volume (MPV), and body weight, which are usually increased even in subclinical hypothyroidism) to further investigate potential hypothyroidism.[26–30] Among these, we noted that only MPV was slightly but significantly increased in both males and females on D180 (Figure S1; Tables S1 and S2).[26] In addition, we investigated the presence of AAV vector genomes in thyroids/parathyroids and pituitary glands from mice randomly selected on both D15 and D180 and found that AAV DNA was present in both thyroid/parathyroid and pituitary glands, although at levels 3 logs lower than those found in the liver, which is the AAV8 target organ (Table S5). Based on these results, we cannot rule out a correlation between AAV2/8.TBG.*hARSB* distribution in these glands and the thyroid morphological changes noted at the histopathological level. Thus, we included thyroid monitoring as part of the follow-up in the clinical trial.

In summary, these data strongly indicate that a single systemic administration of AAV2/8.TBG.*hARSB* was safe and well tolerated in C57/BL6-Tg*ARSBC91S* mice.

AAV2/8.TBG.*hARSB* Biodistribution and Shedding in Mice

A biodistribution study was conducted to assess vector biodistribution, including shedding analysis and germline transmission investigation, following peripheral vein administration of AAV2/8.TBG.*hARSB*. C57/BL6-Tg*ARSBC91S* mice were injected with either the test item at a dose of 2×10^{12} GC/kg (1× the highest clinical trial dose) or the excipient as the control. The analysis was performed at the same time points (D15 and D180) used for the non-clinical safety study (Table 1).

AAV2/8.TBG.*hARSB* biodistribution showed the typical liver tropism of AAV8.[31] Indeed, AAV was mainly sequestered in the liver, although lower levels of vector GC were found in other tissues/organs, especially on D15, as observed in previous biodistribution studies performed with AAV8 in mice, rabbits, and non-human primates (NHPs).[3,8,32–34] AAV2/8.TBG.*hARSB* GCs in the liver of both males and females were at least 3 logs higher than those found in other organs, except for the adrenal gland and gallbladder (about 1–2 logs higher) (Figure 2; Tables S6 and S7). This is in line with previous studies that report the gall bladder and adrenal gland as the most infected organs after the liver in NHPs administered systemically with AAV8.[3,7]

Vector GCs in the liver slightly but significantly decreased in both males and females (−2.2- and −2.7-fold, respectively) on D180, likely because of vector DNA degradation and/or hepatocyte residual proliferation. However, although there was a statistically significant decrease, vector GCs in the liver were quite stable, which supports long-term transduction as also confirmed by the expression data. A general decline from D15 to D180 was observed in most organs (Figure 2; Tables S6 and S7), as previously reported in AAV8-treated mice.[32]

AAV GCs in the liver were statistically different between male and female both on D15 and D180 ($p < 0.001$). This is in line with previous studies, which show that the AAV genome is stably retained in the male liver at levels higher than those observed in female mice.[35]

Similarly to what we observed in the safety study, AAV DNA was found in the thyroids/parathyroids and pituitary glands of both male and female mice. In this case, the amount of vector was 1 log lower than that found in mice from the safety study, as expected based on the different dose of vector injected. In addition, the vector GC declined from D15 to D180 but still persisted at the end of the study (Figure 2; Tables S6 and S7).

At low levels, AAV2/8.TBG.*hARSB* was also found in the nervous system on D15 as previously described in both mice and NHPs that received AAV8.[3,7,32] In conclusion, the biodistribution profile of AAV2/8.TBG.*hARSB* was in line with that observed in previous studies.

Determining AAV vector shedding is central to the safety assessment of early-phase clinical trials; it is especially crucial when using AAV vectors that are injected directly in situ and, unlike retroviral-mediated approaches, there is no possibility for an intermediate ex vivo step.[36] Vector shedding in body fluids such as the plasma, urine,

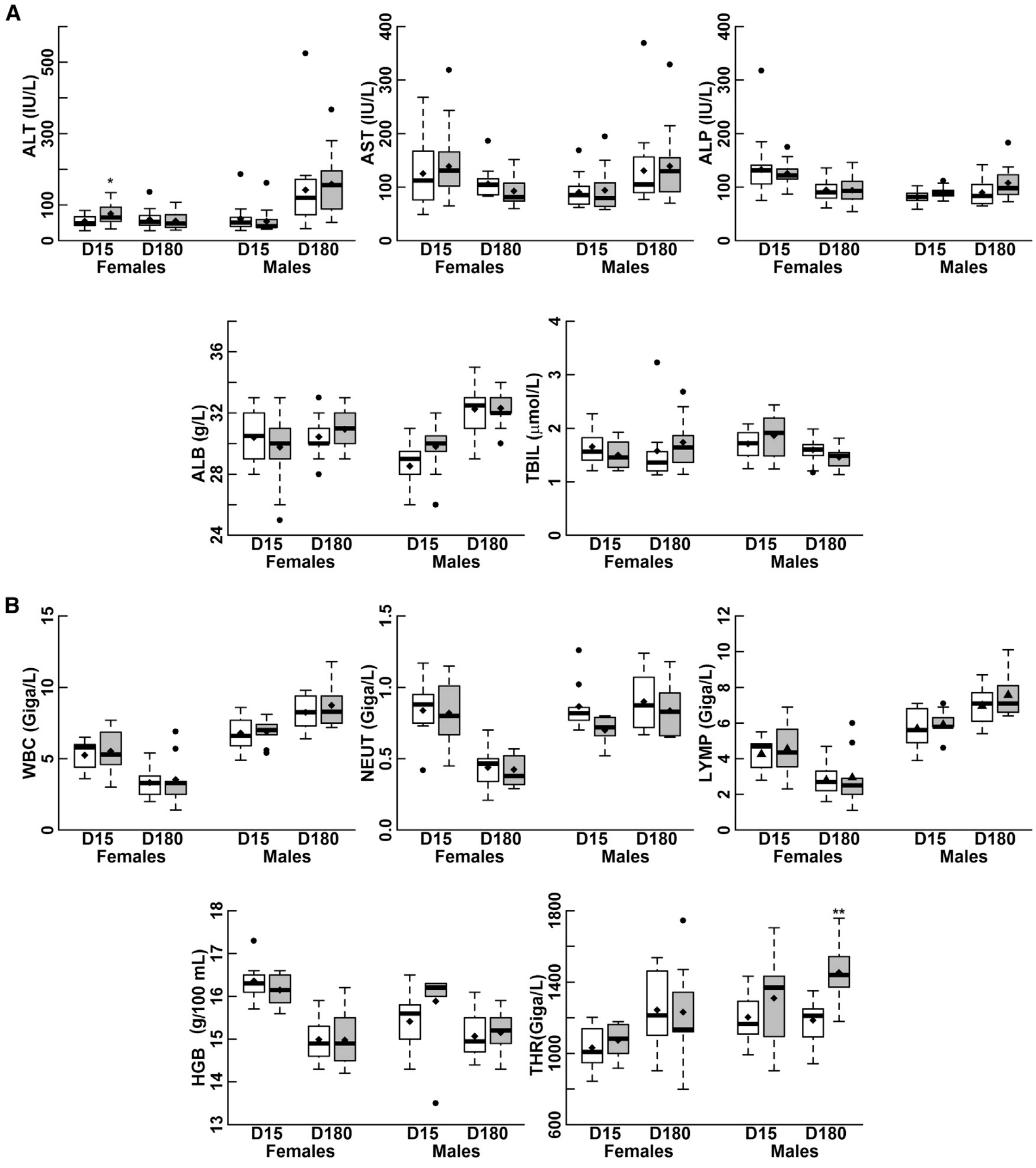

Figure 1. Systemic Delivery of AAV2/8.TBG.*hARSB* Does Not Result in Significant Changes in Blood Chemistry and Hematology

(A and B) Blood chemistry (A) and hematology (B) parameters were evaluated on study days 15 (D15) and 180 (D180) in C57/BL6-Tg*ARSBC91S* male and female mice, which received either the excipient (white bars) or the test item (gray bars). The numbers of animals analyzed for blood chemistry and hematology are reported in the Materials and Methods. Data are reported with box and-whisker plots; the box contains the data points that fall between the first and third quartiles, the horizontal line indicates the median, the diamond indicates the mean, and the brackets delineate 1.5 times the interquartile range (with data outside this range shown as individual points; i.e., black circles). Statistical analyses were performed using either the Wilcoxon-Mann-Whitney U test (ALT, AST, ALP, WBC, LYMP, and THR) or Student's t test (ALB, TBIL, HGB, and NEUT). *$p \leq 0.05$, **$p \leq 0.01$ (versus the excipient group). Excipient: formulation buffer diluted 1:1.5 in 0.9% NaCl saline solution. Test item: AAV2/8.TBG.*hARSB* diluted 1:1.5 in 0.9% NaCl saline solution (dose administered, 2×10^{13} GC/kg). ALB, albumin; ALT, alanine aminotransferase; ALP, alkaline phosphatase; AST, aspartate aminotransferase; HGB, hemoglobin; LYMP, lymphocyte; NEUT, neutrophil; TBIL, total bilirubin; THR, thrombocyte; WBC, white blood cell count.

and stool was analyzed as part of the biodistribution study. The AAV DNA was only transiently found in the stool up to D14 and in the urine and plasma up to D37 (Table S8). Specifically, AAV DNA was below the limit of quantification (LOQ) in most animals starting from D11, D4, and D23 in the stool, urine, and plasma, respectively (Table S8). In addition, vector shedding was also evaluated in male

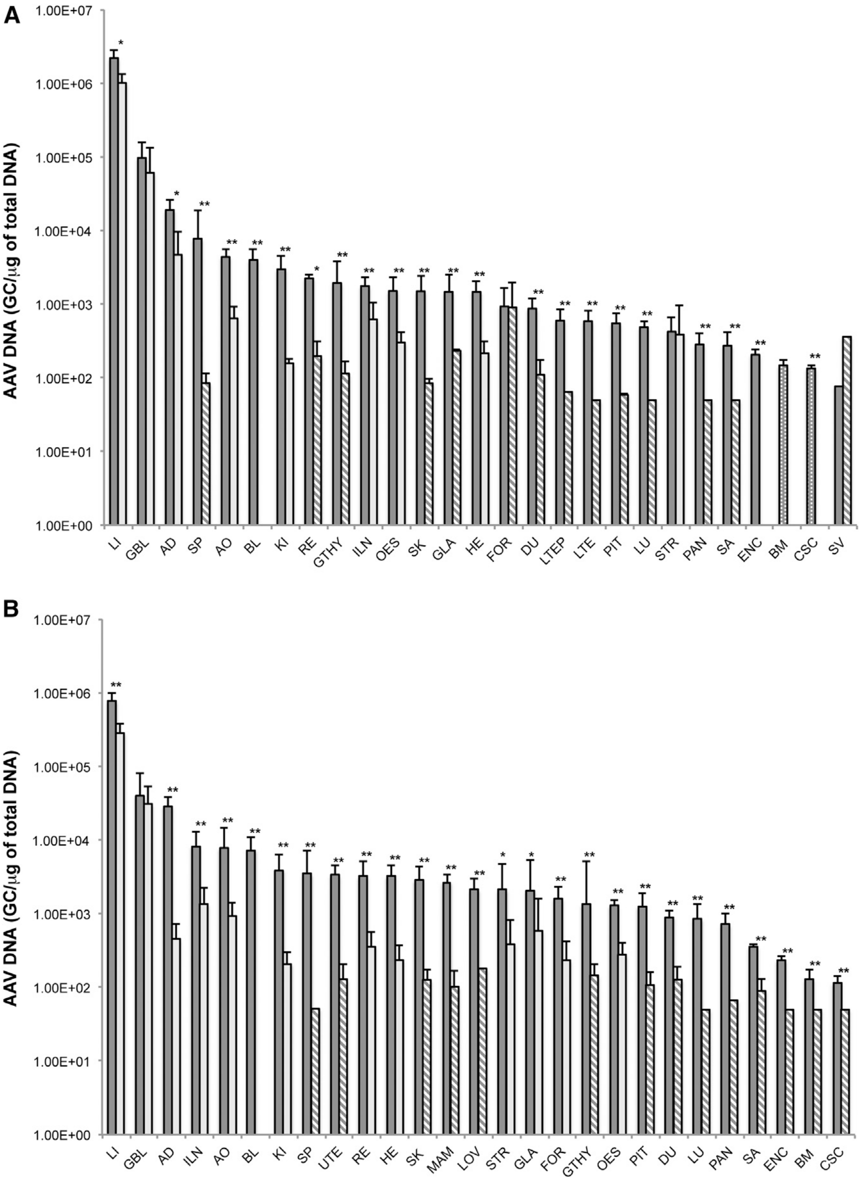

Figure 2. AAV2/8.TBG.*hARSB* Biodistribution in C57/BL6-*TgARSBC91S* Mice

(A and B) AAV2/8.TBG.*hARSB* biodistribution in males (A) and females (B) was analyzed by qPCR. Tissues/organs were collected on D15 (dark gray bars) and D180 (light gray bars). Solid bars represent tissues/organs in which all samples were above the limit of quantification (LOQ) of the assay. Non-solid bars represent tissues/organs in which one or more samples were below the LOQ. The LOQ was 50 genome copies (GC)/μg total DNA and the limit of detection (LOD) was 15 GC/μg total DNA. Results are reported as means ± SD and in decreasing order on D15. Five animals were analyzed per each time point. One male mouse whose organs were collected on D15 was excluded from statistical analysis because the data deviate importantly from other animals, likely because of improper test administration. Statistical analysis was performed using the one-sided Wilcoxon-Mann-Whitney test, assuming LOQ and LOD values for analysis. $^{*}p < 0.05$, $^{**}p < 0.01$ (versus D15). AD, adrenal gland; AO, aorta; BL, blood; BM, bone marrow; CSC, cervical spinal cord; DU, duodenum; ENC, brain; FOR, forestomach; GBL, gallbladder; GLA, glandular stomach; GTHY, thyroid with parathyroid; HE, heart; ILN, inguinal lymph node; KI, kidney; LI, liver; LOV, left ovary; LTE, left testis; LTEP, left tail epididymis; LU, lung; MAM, mammary gland; OES, esophagus; PAN, pancreas; PIT, pituitary gland; RE, rectum; SA, salivary gland; SK, skin; SP, spleen; STR, sternum; SV, seminal vesicles; UTE, uterus.

rabbits, where we observed that AAV2/8.TBG.*hARSB* is only transiently present in body fluids, with kinetics similar to that observed in mice (data not shown).

More importantly, shedding data are available from NHPs and humans systematically administered AAV2/8 vector doses similar to those we propose to use in our clinical trial. Indeed, Nathwani et al.[3,37] showed that vector genomes were cleared from NHP body fluids 10 days after the infusion. Peripheral vein administration of 2×10^{12} GC/kg resulted in a significant viremia in all macaques, which gradually declined to undetectable levels by day 10. Vector DNA was also detectable in the saliva, urine, and stool of all animals in this dose group, but at levels that were at least a log lower than those detected in the plasma.[3] In the hemophilia B clinical trial, which used i.v. administration of AAV2/8 at the same doses we propose to use in our trial, scAAV2/8-LP1-hFIXco vector DNA was detectable in the plasma, saliva, urine, and stool of patients within 72 hr of vector infusion and up to but not after day 20 in all participants; the magnitude and duration of AAV shedding into the plasma, saliva, stool, and urine appeared to be dependent on the dose administered.[2,4] Therefore, based on previous preclinical and clinical data from us and others, the risk of horizontal transmission to third parties and/or to the environment is restricted to a limited time window after administration.

Germline Transmission in Mice and Rabbits

The risk of germline transmission was initially investigated as part of the biodistribution study in mice by assessing the presence of AAV vector genomes in mouse gonads. Biodistribution analysis revealed that vector DNA was present in the gonads of both females and males but at lower levels (3 logs) than those observed in the liver. A robust reduction, but nonetheless persistent level, of vector DNA was observed from D15 to D180 (Figure 2; Tables S6 and S7). Our findings are in line with previous studies, which showed that AAV vectors spread to the gonads of mice and other species (e.g., rabbits and

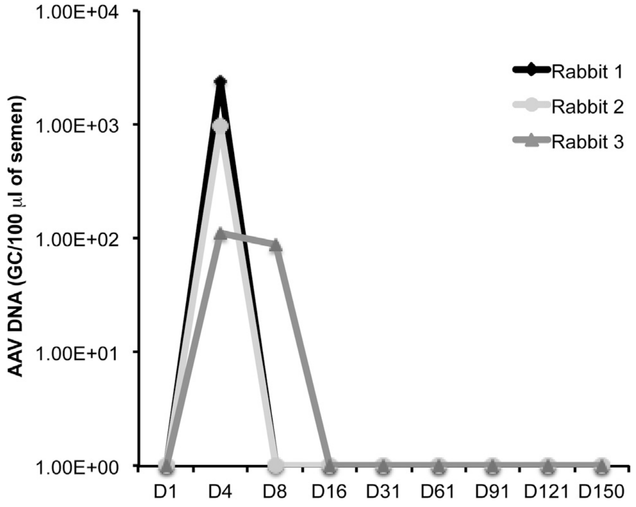

Figure 3. AAV2/8.TBG.*hARSB* Shedding in the Semen of Male Rabbits
AAV2/8.TBG.*hARSB* DNA was analyzed by qPCR in the semen of adult New Zealand males rabbits injected with either AAV2/8.TBG.*hARSB* vector at a dose of 2×10^{12} GC/kg (n = 3) or with the excipient as the control (n = 2). AAV DNA was undetectable in control rabbits receiving the excipient. Vector DNA is reported as genome copies (GC) per 100 μL of semen. The limit of quantification (LOQ) of qPCR was 30 GC/well; the limit of detection (LOD) was 9 GC/well. D, days post-injection.

NHPs) following i.v. administration.[3,7,32,33,37–40] In general, AAV vectors have been found to persist in gonads for long periods up to more than 1 year in both mice and rabbits.[32,33,39] In particular, Chen et al.[32] observed a gradual reduction, but nonetheless persistent levels, of vector DNA in both ovaries and testes from 3–14 days up to 180 days after AAV8 systemic administration, which is in line with our data.

The presence of AAV DNA in the gonads required a further investigation of germline transmission in both sexes. In male animals, the investigation of germline transmission can be accomplished by evaluating the presence of AAV in sperm at various time points based on the duration of spermatogenesis. Failure to detect the recurrence of AAV in the semen argues against the possibility of transduction of the early spermatogenesis precursor exposed to AAV8 during the blood dissemination to the gonads. Since external sperm collection was not possible in mice, we further investigated the risk of germline transmission in male rabbits, which are a widely used and well-suitable animal model (Table 1).[33,38] We injected New Zealand adult rabbits with either 2×10^{12} GC/kg test item (n = 3) or with the excipient as the control (n = 2). At euthanization (D150), AAV vector DNA was found in the liver ($1.8 \times 10^5 \pm 7.7 \times 10^4$), testes ($4.1 \times 10^3 \pm 1.4 \times 10^3$), and epididymis ($2.8 \times 10^2 \pm 1.1 \times 10^2$ and $1.4 \times 10^3 \pm 1.4 \times 10^3$ in the tail and head, respectively), which confirms vector spreading to the liver and gonads. Sperm was collected at different time points, taking the duration of spermatogenesis (which is 42–48 days in rabbits) into account. Vector shedding in rabbit semen was transient and no late recurrence of AAV vector DNA was observed over three cycles of spermatogenesis. Specifically, AAV vector DNA rapidly disappeared in the sperm of male rabbits after D8 (Figure 3), which minimizes the risk of germline cell alteration. These data are in line with previous studies, which show that vector shedding in rabbit semen is transient and no recurrence of AAV sequences is observed over up to 63 consecutive cycles of spermatogenesis.[33,38,41,42]

The kinetics of AAV2/8.TBG.*hARSB* clearance from rabbit semen was remarkably faster than that previously reported for AAV8 at similar doses.[33] However, several factors may influence the kinetics of AAV clearance, such as a higher frequency of semen collection or age-related anatomical variations.[33,42]

In addition, experiments performed with AAV serotype 2 (AAV2) showed that the offspring from mouse sperm exposed to a high concentration of AAV2 and produced by in vitro fertilization and embryo transfer did not carry detectable levels of AAV genomes.[43] Moreover, AAV2 was not detected in either sperm isolated from testes of newborn mice injected systemically or their offspring.[39] Finally, it has also been demonstrated that murine spermatogonia directly exposed to AAV fail to be transduced in vitro.[41] Therefore, our study and previous studies conducted in male animals do not support evidence of AAV germline transmission.[33,39–43]

Conversely, since non-invasive methods are not available for germline cell analysis in female animals, we implemented biodistribution data in gonads by performing in situ hybridization analysis of ovaries from mice euthanized on D15 and D180 using probes complementary to the AAV2/8.TBG.*hARSB* sequence. AAV signal was observed in oocyte nuclei from mice treated with the test item (three of three mice on D15 and two of three mice on D180) but not in those treated with the excipient or those in the assay negative controls at both time points. Therefore, to further investigate the risk of germline transmission in females, we conducted a breeding study. C57/BL6-Tg*ARSBC91S* or C57/BL6 females were injected with either 2×10^{12} GC/kg test item (n = 8) or with the excipient (n = 7) as the control. Injected females were bred 2 or 8 weeks later with naive males of the same strain. AAV2/8.TBG.*hARSB* DNA was analyzed by real-time qPCR in the liver of both injected females and their offspring. AAV DNA was found in the liver from females injected with AAV2/8.TBG.*hARSB* (1.5 ± 0.5 GC/molecule of diploid genome) but not in the liver of mice (n = 38) born from these females or in females injected with the excipient and their offspring (n = 30).

This is in line with the predominantly non-integrating nature of AAV vectors and with a previous study, which reported no evidence of AAV in pups born from female mice injected with AAV2/8 as part of a similar breeding study performed following observation of AAV DNA in mouse ovaries.[40,44,45] Based on this, the risk of inadvertent germline transmission can also be deemed minimal in females. Moreover, pregnancy is reported only in patients with mild MPS VI, who are unlikely to enroll in a phase I gene therapy clinical trial.[46–48]

Importantly, data from clinical trials for hemophilia B based on AAV systemic administration further confirm the low risk of germline transmission in humans. Specifically, delivery of AAV2/2 to the liver via the hepatic artery resulted only in transient vector dissemination in the semen, which eventually disappeared in all patients.[24] Similarly, vector shedding in semen was transiently observed up to 6 weeks in patients that received peripheral administration of AAV2/8.[2,4]

In conclusion, the findings from our study and previous preclinical and clinical studies indicate that there is no evidence to support that recombinant AAV8 can be transferred via germline transmission, although vector genomes are present persistently in the ovary or testis following gene delivery. Nevertheless, where possible, semen of male patients enrolled in the clinical trial will be tested for the presence of AAV over at least one cycle of spermatogenesis, which is approximately 64–74 days in humans, or until the sample turns negative.

AAV2/8.TBG.*hARSB* Expression in Mice

Hepatocyte-specific promoters are important tools in the context of liver gene transfer to avoid unwanted transduction of other cell types. The thyroxine-binding globulin promoter has been used successfully for liver gene therapy experiments due to its specificity for hepatocytes and high levels of transgene expression, especially in combination with AAV2/8 vectors.[49–51] We also previously demonstrated that AAV2/8.TBG.*eGFP*-mediated transduction was mainly restricted to hepatocytes in the liver of both WT and MPS VI rats.[10]

An AAV2/8.TBG.*hARSB* expression study was performed in C57/BL6-Tg*ARSBC91S* mice to assess transgene expression from the vector intended for use in the clinical trial (Table 1). hARSB RNA expression was analyzed by qRT-PCR in mice that received a single systemic administration of either the test item at the dose of 2×10^{12} GC/kg or the excipient as the control.

In both male and female mice, the highest hARSB mRNA levels were found in the liver and then the gall bladder, which are the tissues with the highest number of AAV2/8.TBG.*hARSB* GC. hARSB mRNA levels were higher in males than in females in both the liver and gallbladder at both time points but were significant only on D15, with a slight but significant reduction in hARSB expression in both organs on D180 ($p < 0.01$) (Figure 4; Tables S9 and S10). hARSB mRNA was also highly expressed in both the forestomach and glandular stomach on D15 and in the adrenal gland from males on both D15 and D180, which also matches the AAV GC number. Interestingly, expression was not found in the adrenal gland of females despite the high levels of AAV GC (Figure 4; Tables S9 and S10). Expression was not observed in the thyroid and parathyroid in both sexes, except for one male, and it was undetectable in most samples of the pituitary gland from both males and females (Figure 4; Tables S9 and S10). In summary, this study showed that hARSB mRNA expression from AAV2/8.TBG.*hARSB* was strong in the liver. Interestingly, ARSB expression was found in tissues such as the adrenal gland, stomach, spinal cord, and small intestine, where TBG and alfa-1-microglobulin (from which the TBG promoter is derived) are expressed (http://www.genecard.org). Although hARSB mRNA was found in several tissues on D15, expression stably persisted over time (i.e., up to D180), mainly in the liver. Except for the gall bladder and, more variably, for other few tissues, hARSB expression was not relevant in remaining tissues/organs on D180 (Figure 4; Tables S9 and S10). The low levels of extra-hepatic transgene expression driven by TBG should not be detrimental in the context of MPS VI gene therapy, as ARSB is physiologically ubiquitously expressed.

Non-GLP Dose-Response Study in MPS VI Transgenic Mice

A non-GLP dose-response study was performed to support the dose cohorts proposed for the clinical trial, which were defined based on proof-of-principle studies performed in MPS VI cats.[11,14] The study was performed in adult MPS VI ($ARSB^{-/-}$) transgenic mice treated with a single i.v. administration of the test item at one of the following doses: 2×10^{11}, 6×10^{11}, and 2×10^{12} GC/kg. Five or six mice were treated per each dose cohort. Normal mice (NR) and MPS VI mice (AF) were injected with the excipient or left untreated as the control (Table 1).

The therapeutic efficacy of each dose was evaluated by monthly measurements of serum ARSB and urinary GAGs up to euthanization (D180 post-injection). GAG levels and ARSB activity were also evaluated in tissues at euthanization.

At the latest time point of observation, urinary GAG levels were significantly reduced to 69%, 53%, and 35% of AF levels in the low-, intermediate-, and high-dose cohorts, respectively (Figure 5A). In particular, mice receiving 2×10^{12} GC/kg AAV2/8.TBG.*hARSB* showed normalization of urinary GAGs. This is in line with our previous study in MPS VI mice, which received the same dose of a research-grade vector produced at the Telethon Institute of Genetics and Medicine (TIGEM).[13] Similarly, Alcian blue staining quantification in heart valves and myocardium showed a marked reduction in the cohort receiving the intermediate dose and was even more consistent in the high-dose cohort, where GAG storage was comparable to NR controls (Figure 5B). Mice receiving the low dose of AAV2/8.TBG.*hARSB* showed only a slight reduction in GAG storage (Figure 5B).

Likewise, levels of circulating enzyme were dose dependent, although a statistically significant increase in serum ARSB compared to AF controls was observed only in mice in the highest-dose cohort (Table S11), whose levels were also not statistically different from those of NR controls.

Increased ARSB activity was observed in the liver of all treated mice; detectable activity was variably observed in both the spleen and kidney of treated mice, although at levels lower than those measured in the liver (Table S11), according to our previous study.[13] In addition, ARSB levels in tissues were not statistically different between dose cohorts and AF controls, although a vector dose-dependent response can be observed in the liver. ARSB activity in tissues was significantly

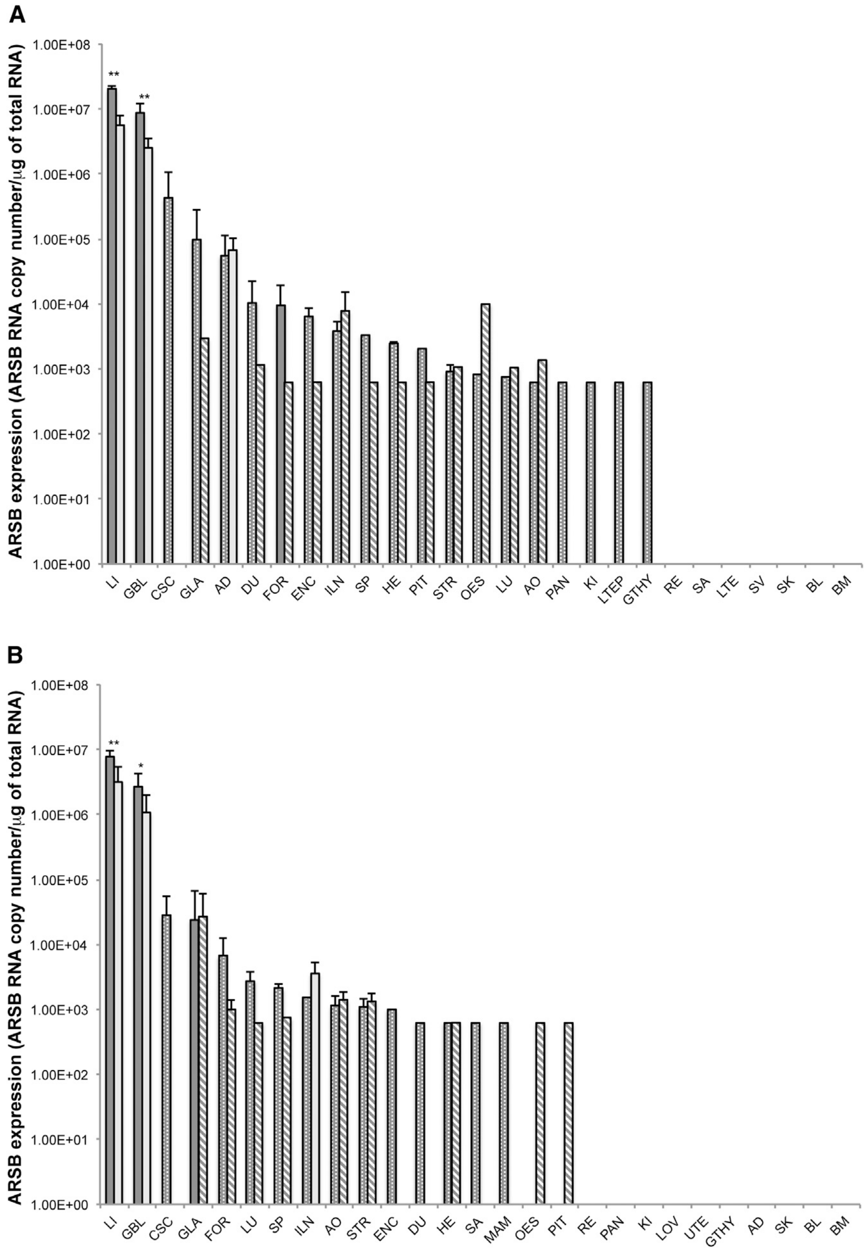

Figure 4. AAV2/8.TBG.*hARSB* Expression in C57/BL6-Tg*ARSBC91S* Mice

(A and B) AAV2/8.TBG.*hARSB* expression in males (A) and females (B) was analyzed by qRT-PCR. Tissues/organs were collected on D15 (dark gray bars) and D180 (light gray bars). Solid bars represent tissues/organs in which all samples were above the limit of quantification (LOQ) of the assay. Non-solid bars represent tissues/organs in which one or more samples were below the LOQ. The LOQ was 625 copies/μg total RNA and the limit of detection (LOD) was 75 copies/μg total RNA. Results are reported as means ± SD and in decreasing order on D15. Five animals were analyzed per each time point. Statistical analysis was performed using the one-sided Wilcoxon-Mann-Whitney test, assuming LOQ and LOD values for analysis. *$p < 0.05$, **$p < 0.01$ (versus D15). AD, adrenal gland; AO, aorta; BL, blood; BM, bone marrow; CSC, cervical spinal cord; DU, duodenum; ENC, brain; FOR, forestomach; GBL, gallbladder; GLA, glandular stomach; GTHY, thyroid with parathyroid; HE, heart; ILN, inguinal lymph node; KI, kidney; LI, liver; LOV; LTE, left testis; LTEP, left tail epididymis; LU, lung; MAM, mammary gland; OES, esophagus; PAN, pancreas; PIT, pituitary gland; RE, rectum; SA, salivary gland; SK, skin; SP, spleen; STR, sternum; SV, seminal vesicles; UTE, uterus.

different from NR controls, with the exception of mice that received the highest dose of vector.

Importantly, a statistically significant reduction in GAG storage was observed in the organs analyzed regardless of ARSB activity levels (Table S11), as previously shown in mice administered research-grade AAV2/8.TBG.*hARSB*.[13] Specifically, GAG levels in the liver and spleen of each dose cohort were similar to normal controls. A dose-dependent response was observed in the kidney, where GAG levels were not statistically different from those of normal controls only in the intermediate- and highest-dose cohorts. These findings support those previously found in our studies in MPS VI cats, rats, and mice, showing that low levels of tissue ARSB activity are sufficient to achieve lysosomal storage clearance.[11–13]

In conclusion, a dose-dependent response was observed in MPS VI transgenic mice. The maximal therapeutic efficacy was achieved at the dose of 2×10^{12} GC/kg with normalization of GAGs in the urine, visceral tissues, and myocardium and marked to complete clearance of GAG storage in the heart valves (Figure 5; Table S11). Similar but more variable results were obtained in mice that received the intermediate (6×10^{11} GC/kg) dose of the test item (Figure 5; Table S11). Our findings also suggest that the 2×10^{11} GC/kg dose can be considered as the minimal effective dose from a risk/benefit perspective. Indeed, although circulating ARSB was consistently detectable in only two of four mice of this dose cohort, urinary GAGs were reduced by 30% on average (Figure 5A) and GAG storage was significantly reduced in the liver, spleen, and kidney (although more variably) (Table S11). While GAG clearance in the myocardium was variable and related to serum ARSB, a minimal reduction was observed in heart valves (Figure 5B). These data confirm what we observed in MPS VI cats that received 2×10^{11} GC/kg vector.[11,14] Overall, our findings in MPS VI cats and mice treated with research-grade or GMP-like vectors, respectively, show that the therapeutic doses of AAV2/8.TBG.*hARSB* range between 2×10^{11} and 2×10^{12} GC/kg.

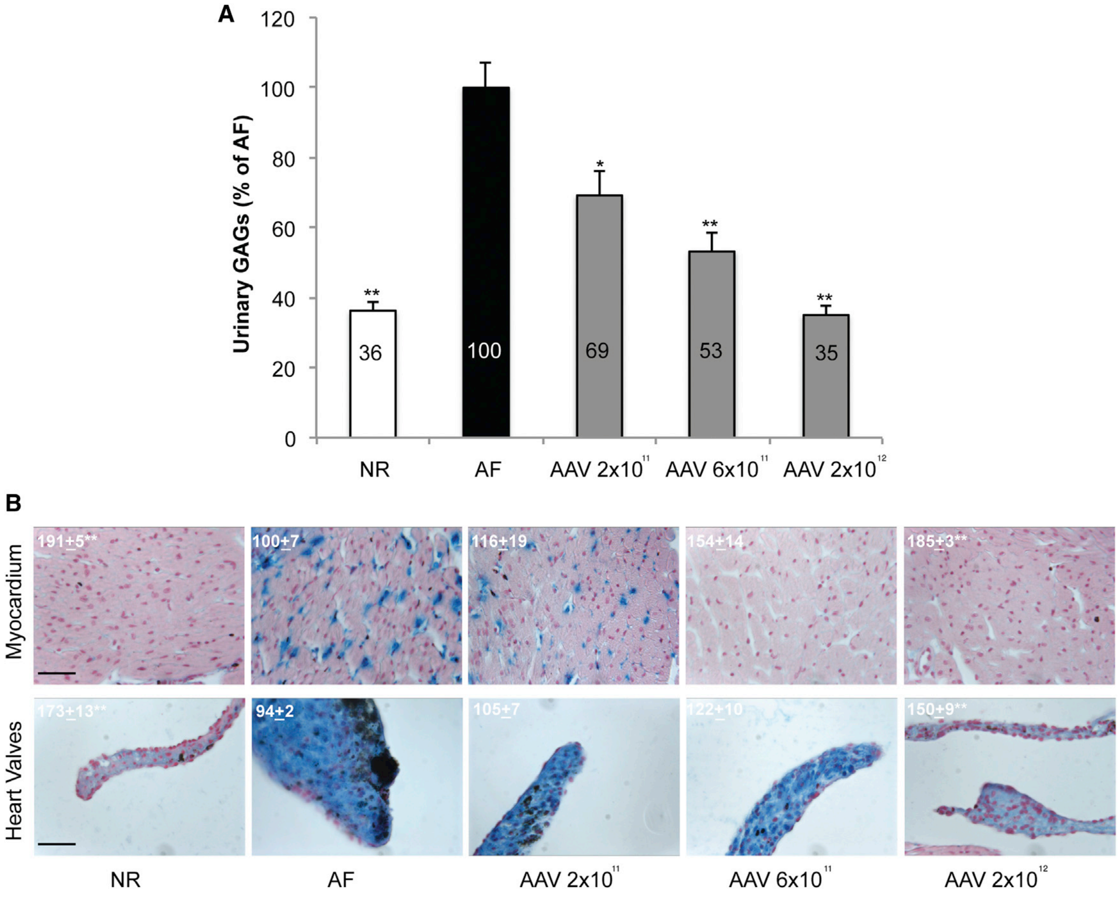

Figure 5. GAG Reduction in Urine, Heart Valves, and Myocardium of Mice Treated with AAV2/8.TBG.*hARSB*

(A) Urinary GAGs were measured 6 months post-injection in MPS VI mice (gray bars), which received 2×10^{11} (AAV 2×10^{11}), 6×10^{11} (AAV 6×10^{11}), or 2×10^{12} (AAV 2×10^{12}) GC/kg AAV2/8.TBG.*hARSB*, and in age-matched normal (NR, white bars) and affected (AF, black bars) controls. Urinary GAG levels measurements were averaged for all animals within the same group of treatment and the resulting value is reported as a percentage of age-matched AF controls, as indicated inside each bar. Results are represented as means ± SEM (NR, n = 14; AF, n = 8; AAV 2×10^{11}, n = 4; AAV 6×10^{11}, n = 4; and AAV 2×10^{12}, n = 5). (B) Reduction in GAG storage in the heart valves and myocardium was evaluated by Alcian blue staining of histological sections obtained from AAV-treated MPS VI mice and from normal (NR) and affected (AF) controls. All MPS VI treated mice were included in the histological analysis. Representative images are shown. The magnification is ×40. The scale bar represents 40 μm. Alcian blue was quantified using ImageJ software by measuring RGB intensity on images of histological sections. Three different areas were randomly selected for each valve. In addition, three areas corresponding to Alcian blue spots were randomly selected per each myocardial section. Where Alcian blue spots were not present (e.g., in NR and some treated mice), three equivalent areas were randomly selected. Results are reported inside each representative image as means ± SEM. Statistical comparisons were made using one-way ANOVA and the Tukey post hoc test. $^{*}p < 0.05$, $^{**}p < 0.01$ (versus AF).

Conclusions

Our data support the safety of a recombinant AAV8 (i.e., AAV2/8.TBG.*hARSB*) for treatment of MPS VI. The vector lot used in the studies presented here was produced according to a process that is similar to the GMP process employed for the clinical lot. We did not observe any mortality or signs of toxicity that could be related to the vector quality in our studies. In summary, our studies show that a single i.v. administration of AAV2/8.TBG.*hARSB* is safe and well tolerated in animals. In addition, the biodistribution and expression data presented here clearly show that efficient and prolonged gene transfer to the liver is achievable with AAV8 following systemic administration. Finally, results from the efficacy dose-response study confirm those previously obtained in both MPS VI cats and mice treated with research-grade AAV8 vectors and allow investigators to define the range of doses to be tested in humans.

Overall and in combination with our previous proof-of-principle studies, these data further support the use of AAV2/8.TBG.*hARSB* for treatment of MPS VI and pave the way for a phase I/II clinical trial to test its safety and efficacy in MPS VI subjects.

MATERIALS AND METHODS

Recombinant AAV Production

A single AAV2/8.TBG.*hARSB* vector lot was used in the GLP non-clinical safety studies and in the dose-response study. This lot was

produced with a process that is similar to the GMP process used for the clinical lot and was adapted from Lock et al.[20] Production was conducted under the supervision of REGENXBIO, who appointed the Weill Medical College of Cornell University Belfer Gene Therapy Core facility for manufacturing and quality control.

Briefly, the AAV2/8.TGB.*hARSB* vector was generated by triple transfection of monolayers of derivative HEK293T cells grown in cell stacks (Corning), using PEI (PolyPlus) under serum-free conditions.

On day 5 post-transfection, cells were treated with benzonase (EMD Millipore) followed by salt adjustment. The supernatant was harvested via depth filtration through a 0.5-μm filter (Pall) and concentrated by TFF. Crude virus concentrated by TFF was centrifuged and the supernatant was loaded onto iodixanol step gradient solutions (OptiPrep; Sigma-Aldrich) in PBS. The gradient steps were 4 mL 15%, 9 mL 25%, 9 mL 40%, and 5 mL 54% iodixanol formed in a 40-mL Quick-Seal centrifugation tube (Beckman Instruments). The tubes were ultra-centrifuged for 70 min at 350,000 $\times$ *g* in a 70Ti rotor (Beckman Instruments) at 18°C and the gradients were fractionated. Diluted fractions were then concentrated and exchanged into PBS-based final formulation buffer. The genome copy titer was determined by qPCR. The concentration was then adjusted to a target of 1.0 $\times$ 10^{13} GC/mL with formulation buffer. The vector preparation was aliquoted and stored at −80 C°. Quality control was performed on this lot to assess sterility, purity, potency, and potential process-related impurities. Major results are reported in Table 2.

This lot was shipped to the Centre de Recherches Biologiques (CERB) test facility and to TIGEM.

Test Facility, Test Sites, and Animals

Non-clinical safety and toxicology studies, except for RNA analysis, were performed in compliance with guidelines concerning GLP published on March 14, 2000 by the French Ministry of Social Affairs and National Solidarity, State Secretariat for Health. These guidelines are in accordance with Directive 2004/10/EC and are accepted by the US Food and Drug Administration (FDA) and Japanese regulatory authorities. All studies were carried out according to European Medicines Agency (EMA) guidelines.

Non-clinical safety, biodistribution (including shedding and germline transmission investigation), and expression studies were performed in adult (p85–95) heterozygous C57/BL6-Tg*ARSBC91S* mice, a mouse model immune tolerant to hARSB that was developed in collaboration with Taconis Artemis and expanded later by Taconis Europe. The model is a WT C57/BL6 mouse that harbors an *hARSB* ORF with the C91S point mutation in the (ROSA)26sor locus.[21,22] This mutation inactivates the ARSB enzymatic activity, preserving the protein conformation and thus making this mouse immune tolerant to WT hARSB.

An ad hoc germline transmission study was conducted in adult New Zealand male rabbits (aged 6 months or older) (HyPharm).

Animals were injected and housed at the CERB test facility. Analyses were conducted both at the test facility and at additional selected test sites. Specifically, general health and clinical sign evaluation, hematology, clinical chemistry, necropsy, and sample collection for other studies were performed at CERB. Histotechnology and histopathology were performed at Propath UK and JPF Consultancy, respectively. AAV DNA analysis for biodistribution, shedding, and germline transmission studies and RNA analysis for the expression study were performed at Genosafe. In situ hybridization was conducted at Histalim.

MPS VI transgenic adult (p45–p73) mice were used in a non-GLP dose-response study. MPS VI mice were maintained at the Cardarelli Hospital Animal House and collected samples were analyzed at TIGEM and the Federico II University Department of Translational Medicine Medical Genetics Laboratory. Animals were raised in accordance with institutional animal care and use committee (IACUC) guidelines for the care and use of animals in research. This mouse model carries a targeted disruption of the ARSB locus and is made immune tolerant to human ARSB by random transgenic insertion of the *C91S hARSB* mutant, resulting in the production of inactive hARSB.[21,52]

Vector Formulation and Administration

Based on the clinical trial protocol, the clinical lot of AAV2/8.TBG.*hARSB* will be diluted in 0.9% sterile NaCl saline solution (supplemented with 0.25% human serum albumin [HSA]) for infusion in patients and the final volume of infusion will be calculated based on the patient's weight as 3 mL/kg. To be as comparable as possible to the clinical lot, the AAV2/8.TBG.*hARSB* vector in its final formulation buffer (which was supplied at the titer of 1 $\times$ 10^{13} GC/mL) was diluted 1:1.5 or 1:15 in 0.9% NaCl saline solution in order to inject 3 mL/kg body weight and to achieve the final dose of 2 $\times$ 10^{13} GC/kg for the non-clinical safety study and 2 $\times$ 10^{12} GC/kg for the biodistribution, expression, and germline transmission studies. For the dose-response study, the vector lot was diluted 1:15, 1:50, and 1:150 in 0.9% NaCl saline solution for the 2 $\times$ 10^{12}, 6 $\times$ 10^{11}, and 2 $\times$ 10^{11} GC/kg dose cohorts, respectively. We did not include HSA in the formulation to avoid possible immune responses against the human protein.

The vector lot diluted in 0.9% NaCl saline solution is indicated here as the "test item." The excipient (i.e., the formulation buffer diluted 1:1.5 or 1:15 in 0.9% NaCl solution) was used as the control.

Both the test item and excipient were administered i.v. C57/BL6-Tg*ARSBC91S* received a slow bolus over about 30 s in the caudal vein, while rabbits received a slow bolus over about 1 min in the ear marginal vein. MPS VI mice were injected in the retro-orbital sinus.

Non-clinical Safety Study

We evaluated the impact of test item administration on hematology, clinical chemistry, necropsy, and histopathology on both D15 and D180.

A total of 160 C57/BL6-Tg*ARSBC91S* mice were used in this study: one group of female and male mice (forty plus forty, twenty plus twenty/time point) received a single i.v. administration of 2×10^{13} GC/kg test item, while a second group of female and male mice (40 plus 40, 20 plus 20/time point) received the excipient as the control. Additional animals were treated as a repository backup in case of unexpected deaths or improper vector administration.

Mice were checked for morbidity and mortality twice daily. Clinical observations were performed on the day of injection (D1) before test item administration and daily (e.g., general disposition, behavior, motor activity, and autonomic profile, including respiratory parameters such as bradypnea, polypnea, and dyspnea, were evaluated). A full clinical examination, which included evaluation of the neurological, behavioral, and autonomic profile, was performed once a week during the first 13 weeks and functional and neuro-behavioral tests were performed on D1 before test item administration, during the first week, monthly, and then before each necropsy (D15 and D180). Body weight was recorded on D1 (before test item administration), D3, D5, D7, D9, D14, and then weekly. Food consumption was measured before test item administration and weekly. Ophthalmological examinations were performed on D1 before test item administration and during the last week of the study before each necropsy day (D15 and D180).

Blood samples for hematology and blood chemistry analysis were collected from the retro-orbital sinus of mice under isoflurane anesthesia on both D15 and D180. The numbers of male animals analyzed for blood chemistry were as follows: 12 for ALT, AST, and total bilirubin (TBIL) and 13 for ALB and ALP in the excipient group on D15; 15 for ALT, 14 for AST, 13 for ALP and ALB, and 12 for TBIL in the test item group on D15; and 16 for all parameters, except for TBIL (n = 15), in both the excipient and test item groups on D180. The numbers of female animals analyzed were as follows: 20 for ALT, ALP, and ALB; 18 for AST; and 11 for TBIL in the excipient group on D15; 21 for all parameters, except for TBIL (n = 10), in the test item group on D15; and 16 for all parameters, except for TBIL (n = 8 and 10 in the excipient and test item groups, respectively), in both the excipient and test item groups on D180.

The numbers of male animals analyzed for hematology were as follows: 9 on D15 in both groups and 10 and 9 on D180 in the excipient and test item groups, respectively. The number of female mice analyzed included 7 and 8 mice on D15 and 10 and 9 on D180 in the excipient and test item groups, respectively.

Forty animals (five males plus five females per group per time point) were randomly selected for necropsy and histopathology from mice euthanized for hematology and blood chemistry assessment. Mice were euthanized by subtotal exsanguination following anesthesia by isoflurane inhalation and were then submitted to full necropsy procedures, including an examination of the external surface, all orifices, the cranial cavity, the external surface of the brain, and samples of the spinal cord, thoracic and abdominal cavities and organs, cervical tissues and organs, and the carcass. Organs were weighed after the dissection of fat and other contiguous tissues. Paired organs were weighed together.

Organs and tissues (41 for males and 43 for females) were fixed and preserved in 4% buffered formalin, except for (1) the testes and epididymides, which were fixed in alcoholic Bouin's fluid (about 5 days) and then transferred to ethanol 95%; and (2) the eyes and optic nerves, which were fixed and preserved in Davidson's fluid (about 3 days) and then transferred to 70% ethanol. After fixation, specimens of all selected organs and tissues and those showing macroscopic abnormalities following necropsy were dehydrated and then embedded in blocks of paraffin wax. Sections (thickness about 5 μm) were stained with H&E and analyzed for histopathology.

Additionally, tissues and organs (as reported in Tables S6 and S7) were collected from five males plus five females per treatment group per time point for possible biodistribution assessment. Livers were collected in all study animals for AAV GC analysis, as a control of either the test item or excipient injection.

Biodistribution and Shedding Study

We used a total of 40 mice in this study: 20 (five females plus five males per treatment group per time point) received a single i.v. administration of 2×10^{12} GC/kg test item, and 20 (five females plus give males per treatment group per time point) received the excipient as the control. Additional animals were injected as a repository back up in case of unexpected deaths or improper vector administration.

Organs and tissues reported in Tables S6 and S7 were collected and analyzed on both D15 and D180. With regard to the gonads, one ovary was collected and processed for biodistribution analysis and the other was used for in situ hybridization.

Blood, urine, and stool were collected for shedding analysis. Specifically, blood samples were collected in K3 EDTA tubes and then centrifuged at $1,500 \times g$ for 10 min at +4°C to obtain plasma before test item administration and on D2, D9, D15, D23, and D37. Stools were taken directly in the cages, while urine collection was performed in metabolism cages for a period of about 2 hr after administration of 20 mL/kg tap water. Stool and urine samples were collected and analyzed before test item administration and then at D2, D4, D11, D14, and D22. Further collection (on D60 for plasma and on D37 and D60 for urine and stool) was performed for those samples that were still positive at the last time point of observation, until AAV DNA became undetectable. All fluids and specimens were collected in sterile labeled tube-certified RNase/DNase, DNA, pyrogens, and PCR inhibitors, free and frozen at <−70°C.

Germline Transmission Study in Male Rabbits

New Zealand White adult rabbits (HyPharm) were used to investigate the risk of germline transmission by analyzing vector dissemination

in sperm. Rabbits received a single i.v. administration of either 2×10^{12} GC/kg test item (n = 3) or excipient as the control (n = 2).

Sperm samples were collected from adult male rabbits on D4, D8, D16, D31, D61, D91, D121, and D150 before test item administration, in order to cover three spermatogenesis cycles (with the duration of spermatogenesis being 42–48 days in the rabbit). Sperm was collected through an artificial vagina (Synthèse élevage). Rabbits were euthanized on day 150 post-injection (D151). The liver was also harvested at euthanization per control of injection procedure.

TaqMan qPCR-Based Biodistribution Analysis

Total DNA was isolated using the following kits: NucleoSpin Tissue or NucleoBond AX (Macherey-Nagel) for tissues, NucleoSpin Blood QuickPure (Macherey-Nagel) or the QIAamp DNA Blood Kit (QIAGEN) for blood, the QIAamp DNA Blood Kit (QIAGEN) for sperm (including preliminary treatment with dithiothreitol) and for urine, and the QIAamp Fast DNA Stool Mini Kit (QIAGEN) for stool. DNA was quantified by measuring the optical density (OD) at 260 nm using a spectrophotometer (PowerWave XS; BioTek). DNA samples were analyzed by qPCR, using the ABI PRISM 7900HT sequence detection system (Applied Biosystems). The following primers and TaqMan probe designed in the bovine growth hormone (BGH) region of the AAV2/8.TBG.*hARSB* vector were used: forward primer [Fw], GTTGCCAGCCATCTGTTGTTT (300 nM); reverse primer [Rev], GACAGTGGGAGTGGCACCTT (50 nM); and TaqMan MGB probe, CAGGGTCAAGGAAG (200 nM) (Applied Biosystems).

The LOQ of the assay was 50 GC/μg DNA as recommended by FDA guidelines [ICHQ2(R1)]. This limit corresponds to the lowest number of vector copies that was reliably measured in 1 μg host DNA with repeatability, intermediate precision, and measured bias less than 10% of variability.

The limit of detection (LOD) was 15 GC/μg DNA corresponding to an LOQ $\times$ 3/10, with LOQ = 10 $\times$ background noise and LOD = 3 $\times$ background noise (background noise <5 GC/μg DNA).

Since the range of quantification was from 30 to 10^8, the LOQ and LOD were 30 GC/well (corresponding to the lowest amount of vector GC in the standard curve) and 9 GC/well, respectively, in body fluids where no host DNA was quantified. The normalized target quantity is reported as the number of GC/100 μL fluid. When less than 600 ng host DNA or less than 90 μL for fluids was used, these limits were recalculated as follows: for tissues, 600/analyzed quantity $\times$ LOQ (50) or LOD (15). For fluids, LOQ was recalculated according to the following formula: (LOQ $\times$ 100 μL)/(volume of sample tested/6). LOD was recalculated according to the following formula: (LOD $\times$ 100 μL)/(volume of sample tested/6), where 6 is the ratio between the volume of eluted sample (90 μL) and the volume tested by qPCR (15 μL).

Acceptance criteria were as follows: (1) qPCR efficiency (E) $0.90 \leq E \leq 1.1$, (2) $r^2 \geq 0.99$, and (3) no significant amplification (copy number < LOD) in negative controls of extraction and qPCR.

Inhibition was assessed by monitoring the number of copies of an endogenous reference (i.e., mice albumin gene) compared to a set range (for tissues, total blood, and stools) or by adding a number of known copies of the reference plasmid before extraction (for urine, plasma, and sperm).

hARSB RNA Expression Analysis

hARSB RNA expression was analyzed by qRT-PCR in 10 mice (five males plus five females per each time point) that received a single systemic administration of 2×10^{12} GC/kg test item. Ten mice (five males plus five females per each time point) received the excipient alone as a control. Additional animals were injected as a repository back up in case of unexpected deaths or improper vector administration. Tissues and organs reported in Tables S9 and S10 were collected at each time point (D15 and D180). About 0.2 mL blood samples were split into two RNAprotect Animal Blood Tubes (QIAGEN) containing 100 μL each and were immediately stored frozen at −20°C. Organ samples were completely submerged in RNAlater reagent (Sigma-Aldrich) in sterile tubes certified as being free of RNase/DNase, DNA, pyrogen, and PCR inhibitor. The submerged samples were incubated overnight in a refrigerator at approximately 4 ± 2°C. The RNAlater reagent was then removed, and the samples were snap-frozen in the collection tube and stored at <−70°C.

For RNA extraction, organs were weighed, sliced thinly if necessary, and put in a matrix containing ceramic balls (MP Biomedicals). Afterward, samples were ground in the presence of Qiazol (QIAGEN). Blood samples were incubated at room temperature for 4 hr (2 hr of defrosting and 2 hr of RNA extraction). RNA from bone marrow was extracted with TRIzol LS (Life Technologies) and chloroform (Sigma-Aldrich). RNA integrity was checked using the Experion electrophoresis system (Bio-Rad).

Retro-transcription was performed using SuperScript VILO Master Mix (Thermo Fisher Scientific) on 400 ng RNA (in triplicate) spiked with 1×10^4 copies of RNA of the *Arabidopsis thaliana* exogenous gene (RBCL) to identify any potential inhibition of qRT-PCR reactions. Amplification was performed using the same primers and the TaqMan MGB probe as for the biodistribution study. A synthetic RNA corresponding to the target sequence was used to prepare a standard curve ranging from LOQ to 10^7 RNA vector copies. RNA from mice was used as the carrier for the standard curve. The LOD and LOQ were 187.5 RNA copies and 625 RNA copies/μg respectively. Acceptance criteria were as follows: (1) qPCR efficiency $0.80 \leq E \leq 1.05$, (2) $r^2 \geq 0.8$, and (3) no significant amplification (copy number < LOD) in negative controls of retro-transcription and qPCR.

Given the high number of samples collected, hARSB RNA expression was assessed according to the following plan. First, qRT-PCR analysis was done in all tissues collected on D15 from three males and three females that were randomly selected. Second, for each tissue, if the RNA copy number was greater than or equal to the LOQ of the assay in at least one animal (male or female), all animals (five males and five females) were investigated for that tissue on D15. If not, the

remaining animals were not investigated for that tissue. Additionally, when the amount of RNA tested was less than expected (i.e., <400 ng), all animals (five males and five females) were analyzed on D15 independently of the number of animals in which the hARSB RNA copy number was above the LOQ. Third, only tissues with a mean RNA copy number greater than or equal to the LOQ on D15 were investigated in samples collected on D180 from three males and three females that were randomly selected. Finally, for each tissue, if the RNA copy number was greater than or equal to the LOQ in at least one animal (male or female), all animals were investigated for that tissue on D180. If not, the remaining animals were not investigated for that tissue.

In Situ Hybridization in Ovaries

Right ovaries were collected from female mice (used for biodistribution) on D15 and D180 and were preserved in 10% buffered formalin and then in 70% ethanol. Whole ovaries were processed in Peloris automaton (Leica) and then embedded in paraffin.

For each animal, 10 sections (3- to 5-μm thick) were manufactured and deposited on two Superfrost+ slides. One slide was treated with the AAV probe, which was designed on the BGH poly(A) region. The second slide was processed according to the same protocol without the AAV probe, which was the negative control. Each assay included positive controls (i.e., a slide of ovary labeled with a control mouse probe designed on the sequence of elongation factor 1 [EF1] and a slide of injected mouse liver labeled with the AAV probe).

Slides were scanned at ×20 magnification in bright-field conditions with a Nanozoomer scanner (Hamamatsu). The analysis consisted of the detection of a positive signal in the slide labeled with the AAV probe by comparison with the negative control slide. The RNA in situ hybridization analysis was performed by Histalim.

Breeding Study

C57/BL6-Tg*ARSBC91S* or C57/BL6 female mice were injected with either 2×10^{12} GC/kg AAV2/8.TBG.*hARSB* (n = 8) or the excipient (n = 7) as the control. AAV-injected and control females were bred with naive males of the same strain 2 or 8 weeks after injection. Genomic DNA was extracted from the livers of both injected females and 90- to 180-day-old mice born from these females, using the DNeasy Blood and Tissue extraction kit (QIAGEN).

Real-time qPCR analysis was performed on 100 ng genomic DNA using a set of primers and a TaqMan probe designed on the BGH region of AAV2/8.TBG.*hARSB* and thus specific for the viral genome (Fw, 5′-TCTAGTTGCCAGCCATCTGTTGT-3′; Rev, 5′-TGGGAGTGG CACCTTCCA-3′; and probe, 5′-TCCCCCGTGCCTTCCTTGAC C-3′) and TaqMan universal PCR master mix (Applied Biosystems). Murine beta-actin was also amplified as a control of DNA quality, using specific set of primers and a TaqMan probe (Fw, 5′-CGTTCC GAAAGTTGCCTTTTA-3′; Rev, 5′-CGCCGCCGGGTTTTATA-3′; and probe, 5′-CTCGAGTGGCCGCTGT-3′). Amplification was run on a 7300 Real-Time PCR system (Applied Biosystems) with standard cycles.

Blood, Urine, and Tissue Collection in MPS VI Mice

Blood and urine were collected each month from treated and control mice. Serum samples were collected from the retro-orbital sinus and centrifuged at 10,000 × *g* in a microcentrifuge (Z 216 MK; HERMLE) for 10 min at 4°C to obtain the serum. For urine collections, mice were put in metabolic cages for 24 hr. The urine samples were briefly centrifuged to remove debris and stored at −20°C. Mice were euthanized 5 or 6 months following the start of treatment. A cardiac perfusion with PBS was performed, and the liver, kidney, spleen, and heart were collected. Tissue samples were fixed in a methacarn solution (30% chloroform, 60% methanol, and 10% acetic acid) for 24 hr or frozen in dry ice (for ARSB activity and GAG quantitative assays).

Assays for ARSB Enzymatic Activity Evaluation in Serum and Tissues

Serum ARSB activity was measured by an immune capture assay based on the use of a specific anti-hARSB polyclonal antibody (Covalab), as previously described.[13] Briefly, 96-well plates (Nunclon) were coated with 5 μg/mL in 0.1 M $NaHCO_3$ (100 μL/well) and incubated overnight (O/N) at 4°C. The following day, plates were blocked with 1% milk; after 2 hr of incubation, 50 μL standard and unknown samples (diluted 1:10) was added to each well. Plates were incubated at 4°C O/N. The following day, 100 μL 5 mM 4-methylumbelliferylsulfate potassium salt (4-MUS; Sigma-Aldrich) substrate was added to each well and then incubated at 37°C for 4 hr. The reaction was stopped by the addition of 100 μL stop solution/well (0.2 M glycine). Plates were shaken for 10 min at room temperature and the fluorescence was read (excitation of 365 nm/emission of 460 nm) on a multiplate fluorimeter (Infinite F200; TECAN). Serum ARSB was determined based on a rhARSB (Naglazyme; BioMarin Europe) standard curve and is expressed as picograms per milliliter.

Tissues (i.e., liver, kidney, and spleen) were homogenized in water and protein concentrations were determined with the bicinchoninic acid (BCA) protein assay kit (Pierce Protein Research Products; Thermo Fisher Scientific). The ARSB assay was performed as previously described.[53] Briefly, 30 μg protein was incubated with 40 μL fluorogenic 4-methylumbelliferyl sulfate substrate (12.5 mM; Sigma-Aldrich) for 3 hr at 37°C in the presence of 40 μL silver nitrate (0.75 mM; Carlo Erba), which is known to inhibit the activity of other sulfatases. The reaction was stopped by adding 200 μL carbonate glycine stop buffer and the fluorescence was read as described above.

Quantitative Analyses of GAG Accumulation in Tissues and Urine

Urine samples were diluted 1:50 in water to measure GAG content. 100 μL diluted urine or 250 μg protein extract from the liver, spleen, and kidney was used for the GAG assay, as previously described.[54] GAG concentrations were determined on the basis of a dermatan sulfate standard curve (Sigma-Aldrich). Tissue GAGs were expressed as micrograms of GAG per milligram of protein. Urinary

GAGs were normalized to creatinine content, which was measured with a creatinine assay kit (Quidel). Thus, the units of urinary GAGs are given in micrograms of GAG per micromole of creatinine. Urinary GAGs are reported as the percentage of AF control mice. At the latest time point of observation, the urinary GAG levels were averaged for each group.

Alcian Blue Staining of Tissues

After methacarn fixation, hearts were dehydrated by immersion in increasing concentrations of alcohol (70%, 80%, 90%, 100%) and then in xylene. Hearts were embedded in paraffin and sectioned into 7-μm-thick serial sections on a microtome. Tissue sections were de-paraffinized, rehydrated, then washed in water and stained with 1% Alcian blue (Sigma-Aldrich) in hydrochloric acid for 10 s. Alcian blue staining was quantified by measuring RGB intensity on histological sections using ImageJ software (NIH). RGB may assume integer values from 0 to 255. The more intense the Alcian blue staining, the lower the RGB value. Three different areas were randomly selected in each valve. With regard to the myocardium, three areas corresponding to Alcian blue spots were randomly selected per each section. Where Alcian blue spots were not present (as in NR and some treated mice), three equivalent areas were randomly selected. RGB was measured per each area and then averaged for each animal and each group of animals.

Statistical Analyses

Regarding blood chemistry and hematology, the comparison between treatment groups was performed using Student's t test or the Wilcoxon-Mann-Whitney test, as appropriate. Treatment growth curves was compared by means of an ANOVA for repeated measurements (i.e., test of the time $\times$ treatment interaction). Incidences of macroscopic findings observed at necropsy and microscopic findings in the thyroid were analyzed using Fisher's test. The comparison of biodistribution and expression data at D15 versus D180 was performed using a one-sided Student's t test. One-way ANOVA and the Tukey post hoc test were used to compare treatment groups in the non-GLP dose-response study in mice and in the assessment of AAV2/8.TBG.*hARSB* shedding. All results are reported as means ± SD or means ± SEM. The tests are two sided unless otherwise specified, and the level of significance is 0.05.

SUPPLEMENTAL INFORMATION

Supplemental Information includes one figure and eleven tables and can be found with this article online at http://dx.doi.org/10.1016/j.omtm.2017.07.004.

AUTHOR CONTRIBUTIONS

Conceptualization, R.F., A.A., S.G., M.G.V; Methodology, J.-B.M.; Formal Analysis, S.G. and M.G.V.; Investigation, R.F., M.A., J.-B.M., M.D., and E.N.; Writing – Original Draft, R.F. and M.A.; Writing – Review & Editing, R.F. and A.A.; Supervision, R.F. and A.A.; Project Administration, R.F. and S.P.; Funding Acquisition, V.Z. and A.A.

CONFLICTS OF INTEREST

R.F., M.A., M.D., E.N., S.G., M.G.V., and A.A. declare that no competing financial interests exist. J.-B.M., S.P., and V.Z. have no conflicts of interest beyond being employees of Genosafe.

ACKNOWLEDGMENTS

We gratefully acknowledge REGENXBIO for the production and characterization of the AAV2/8.TBG.*hARSB* vector lot used in this study. We also thank Enrico M. Surace and Bianca Fontanella (TIGEM) for help with study design and Annamaria Carissimo (TIGEM Bioinformatics Core) for analytical support. This work was supported by Fondazione Telethon TGM11MT6, the European Commission Seventh Framework Programme (FP7/2007–2013) (MeuSIX grant 304999) and the Isaac Foundation.

REFERENCES

1. Brunetti-Pierri, N., and Auricchio, A. (2010). Gene therapy of human inherited diseases. In The Metabolic and Molecular Bases of Inherited Diseases, R. Scriver, ed. (McGraw Hill), pp. 1–50.
2. Nathwani, A.C., Reiss, U.M., Tuddenham, E.G., Rosales, C., Chowdary, P., McIntosh, J., Della Peruta, M., Lheriteau, E., Patel, N., Raj, D., et al. (2014). Long-term safety and efficacy of factor IX gene therapy in hemophilia B. N. Engl. J. Med. *371*, 1994–2004.
3. Nathwani, A.C., Rosales, C., McIntosh, J., Rastegarlari, G., Nathwani, D., Raj, D., Nawathe, S., Waddington, S.N., Bronson, R., Jackson, S., et al. (2011). Long-term safety and efficacy following systemic administration of a self-complementary AAV vector encoding human FIX pseudotyped with serotype 5 and 8 capsid proteins. Mol. Ther. *19*, 876–885.
4. Nathwani, A.C., Tuddenham, E.G., Rangarajan, S., Rosales, C., McIntosh, J., Linch, D.C., Chowdary, P., Riddell, A., Pie, A.J., Harrington, C., et al. (2011). Adenovirus-associated virus vector-mediated gene transfer in hemophilia B. N. Engl. J. Med. *365*, 2357–2365.
5. Brantly, M.L., Chulay, J.D., Wang, L., Mueller, C., Humphries, M., Spencer, L.T., Rouhani, F., Conlon, T.J., Calcedo, R., Betts, M.R., et al. (2009). Sustained transgene expression despite T lymphocyte responses in a clinical trial of rAAV1-AAT gene therapy. Proc. Natl. Acad. Sci. USA *106*, 16363–16368.
6. Maguire, A.M., Simonelli, F., Pierce, E.A., Pugh, E.N., Jr., Mingozzi, F., Bennicelli, J., Banfi, S., Marshall, K.A., Testa, F., Surace, E.M., et al. (2008). Safety and efficacy of gene transfer for Leber's congenital amaurosis. N. Engl. J. Med. *358*, 2240–2248.
7. Greig, J.A., Limberis, M.P., Bell, P., Chen, S.J., Calcedo, R., Rader, D.J., and Wilson, J.M. (2017). Non-clinical study examining AAV8.TBG.hLDLR vector-associated toxicity in chow-fed wild-type and LDLR(+/-) rhesus macaques. Hum. Gene Ther. Clin. Dev. *28*, 39–50.
8. Greig, J.A., Limberis, M.P., Bell, P., Chen, S.J., Calcedo, R., Rader, D.J., and Wilson, J.M. (2017). Nonclinical pharmacology/toxicology study of AAV8.TBG.mLDLR and AAV8.TBG.hLDLR in a mouse model of homozygous familial hypercholesterolemia. Hum. Gene Ther. Clin. Dev. *28*, 28–38.
9. Alliegro, M., Ferla, R., Nusco, E., De Leonibus, C., Settembre, C., and Auricchio, A. (2016). Low-dose gene therapy reduces the frequency of enzyme replacement therapy in a mouse model of lysosomal storage disease. Mol. Ther. *24*, 2054–2063.
10. Cotugno, G., Annunziata, P., Barone, M.V., Karali, M., Banfi, S., and Auricchio, A. (2012). Impact of age at administration, lysosomal storage, and transgene regulatory elements on AAV2/8-mediated rat liver transduction. PLoS ONE *7*, e33286.
11. Cotugno, G., Annunziata, P., Tessitore, A., O'Malley, T., Capalbo, A., Faella, A., Bartolomeo, R., O'Donnell, P., Wang, P., Russo, F., et al. (2011). Long-term amelioration of feline mucopolysaccharidosis VI after AAV-mediated liver gene transfer. Mol. Ther. *19*, 461–469.
12. Cotugno, G., Tessitore, A., Capalbo, A., Annunziata, P., Strisciuglio, C., Faella, A., Aurilio, M., Di Tommaso, M., Russo, F., Mancini, A., et al. (2010). Different serum enzyme levels are required to rescue the various systemic features of the mucopolysaccharidoses. Hum. Gene Ther. *21*, 555–569.

13. Ferla, R., Claudiani, P., Cotugno, G., Saccone, P., De Leonibus, E., and Auricchio, A. (2014). Similar therapeutic efficacy between a single administration of gene therapy and multiple administrations of recombinant enzyme in a mouse model of lysosomal storage disease. Hum. Gene Ther. *25*, 609–618.

14. Ferla, R., O'Malley, T., Calcedo, R., O'Donnell, P., Wang, P., Cotugno, G., Claudiani, P., Wilson, J.M., Haskins, M., and Auricchio, A. (2013). Gene therapy for mucopolysaccharidosis type VI is effective in cats without pre-existing immunity to AAV8. Hum. Gene Ther. *24*, 163–169.

15. Tessitore, A., Faella, A., O'Malley, T., Cotugno, G., Doria, M., Kunieda, T., Matarese, G., Haskins, M., and Auricchio, A. (2008). Biochemical, pathological, and skeletal improvement of mucopolysaccharidosis VI after gene transfer to liver but not to muscle. Mol. Ther. *16*, 30–37.

16. Neufeld, E., and Muenzer, J. (2010). The mucopolysaccharidoses. In The Online Metabolic and Molecular Bases of Inherited Disease, R. Scriver, ed. (McGraw-Hill), pp. 1–73.

17. Desnick, R.J., and Schuchman, E.H. (2012). Enzyme replacement therapy for lysosomal diseases: lessons from 20 years of experience and remaining challenges. Annu. Rev. Genomics Hum. Genet. *13*, 307–335.

18. Giugliani, R., Lampe, C., Guffon, N., Ketteridge, D., Leao-Teles, E., Wraith, J.E., Jones, S.A., Piscia-Nichols, C., Lin, P., Quartel, A., and Harmatz, P. (2014). Natural history and galsulfase treatment in mucopolysaccharidosis VI (MPS VI, Maroteaux-Lamy syndrome)—10-year follow-up of patients who previously participated in an MPS VI survey study. Am. J. Med. Genet. A *164A*, 1953–1964.

19. Schlander, M., and Beck, M. (2009). Expensive drugs for rare disorders: to treat or not to treat? The case of enzyme replacement therapy for mucopolysaccharidosis VI. Curr. Med. Res. Opin. *25*, 1285–1293.

20. Lock, M., Alvira, M., Vandenberghe, L.H., Samanta, A., Toelen, J., Debyser, Z., and Wilson, J.M. (2010). Rapid, simple, and versatile manufacturing of recombinant adeno-associated viral vectors at scale. Hum. Gene Ther. *21*, 1259–1271.

21. Brooks, D.A., Robertson, D.A., Bindloss, C., Litjens, T., Anson, D.S., Peters, C., Morris, C.P., and Hopwood, J.J. (1995). Two site-directed mutations abrogate enzyme activity but have different effects on the conformation and cellular content of the N-acetylgalactosamine 4-sulphatase protein. Biochem. J. *307*, 457–463.

22. Zambrowicz, B.P., Imamoto, A., Fiering, S., Herzenberg, L.A., Kerr, W.G., and Soriano, P. (1997). Disruption of overlapping transcripts in the ROSA beta geo 26 gene trap strain leads to widespread expression of beta-galactosidase in mouse embryos and hematopoietic cells. Proc. Natl. Acad. Sci. USA *94*, 3789–3794.

23. Wang, L., Wang, H., Bell, P., McMenamin, D., and Wilson, J.M. (2012). Hepatic gene transfer in neonatal mice by adeno-associated virus serotype 8 vector. Hum. Gene Ther. *23*, 533–539.

24. Manno, C.S., Pierce, G.F., Arruda, V.R., Glader, B., Ragni, M., Rasko, J.J., Ozelo, M.C., Hoots, K., Blatt, P., Konkle, B., et al. (2006). Successful transduction of liver in hemophilia by AAV-Factor IX and limitations imposed by the host immune response. Nat. Med. *12*, 342–347.

25. Mingozzi, F., and High, K.A. (2013). Immune responses to AAV vectors: overcoming barriers to successful gene therapy. Blood *122*, 23–36.

26. Iwen, K.A., Schröder, E., and Brabant, G. (2013). Thyroid hormones and the metabolic syndrome. Eur. Thyroid J. *2*, 83–92.

27. Laway, B.A., War, F.A., Shah, S., Misgar, R.A., and Kumar Kotwal, S. (2014). Alteration of lipid parameters in patients with subclinical hypothyroidism. Int. J. Endocrinol. Metab. *12*, e17496.

28. Coban, E., Yazicioglu, G., and Ozdogan, M. (2007). Platelet activation in subjects with subclinical hypothyroidism. Med. Sci. Monit. *13*, CR211–CR214.

29. Erikci, A.A., Karagoz, B., Ozturk, A., Caglayan, S., Ozisik, G., Kaygusuz, I., and Ozata, M. (2009). The effect of subclinical hypothyroidism on platelet parameters. Hematology *14*, 115–117.

30. Yilmaz, H., Ertuğrul, O., Ertuğrul, B., and Ertuğrul, D. (2011). Mean platelet volume in patients with subclinical hypothyroidism. Platelets *22*, 143–147.

31. Thomas, C.E., Storm, T.A., Huang, Z., and Kay, M.A. (2004). Rapid uncoating of vector genomes is the key to efficient liver transduction with pseudotyped adeno-associated virus vectors. J. Virol. *78*, 3110–3122.

32. Chen, S.J., Sanmiguel, J., Lock, M., McMenamin, D., Draper, C., Limberis, M.P., Kassim, S.H., Somanathan, S., Bell, P., Johnston, J.C., et al. (2013). Biodistribution of AAV8 vectors expressing human low-density lipoprotein receptor in a mouse model of homozygous familial hypercholesterolemia. Hum. Gene Ther. Clin. Dev. *24*, 154–160.

33. Favaro, P., Downey, H.D., Zhou, J.S., Wright, J.F., Hauck, B., Mingozzi, F., High, K.A., and Arruda, V.R. (2009). Host and vector-dependent effects on the risk of germline transmission of AAV vectors. Mol. Ther. *17*, 1022–1030.

34. Pañeda, A., Lopez-Franco, E., Kaeppel, C., Unzu, C., Gil-Royo, A.G., D'Avola, D., Beattie, S.G., Olagüe, C., Ferrero, R., Sampedro, A., et al. (2013). Safety and liver transduction efficacy of rAAV5-cohPBGD in nonhuman primates: a potential therapy for acute intermittent porphyria. Hum. Gene Ther. *24*, 1007–1017.

35. Davidoff, A.M., Ng, C.Y., Zhou, J., Spence, Y., and Nathwani, A.C. (2003). Sex significantly influences transduction of murine liver by recombinant adeno-associated viral vectors through an androgen-dependent pathway. Blood *102*, 480–488.

36. Le Guiner, C., Moullier, P., and Arruda, V.R. (2011). Biodistribution and shedding of AAV vectors. Methods Mol. Biol. *807*, 339–359.

37. Nathwani, A.C., Gray, J.T., McIntosh, J., Ng, C.Y., Zhou, J., Spence, Y., Cochrane, M., Gray, E., Tuddenham, E.G., and Davidoff, A.M. (2007). Safe and efficient transduction of the liver after peripheral vein infusion of self-complementary AAV vector results in stable therapeutic expression of human FIX in nonhuman primates. Blood *109*, 1414–1421.

38. Favaro, P., Finn, J.D., Siner, J.I., Wright, J.F., High, K.A., and Arruda, V.R. (2011). Safety of liver gene transfer following peripheral intravascular delivery of adeno-associated virus (AAV)-5 and AAV-6 in a large animal model. Hum. Gene Ther. *22*, 843–852.

39. Jakob, M., Mühle, C., Park, J., Weiss, S., Waddington, S., and Schneider, H. (2005). No evidence for germ-line transmission following prenatal and early postnatal AAV-mediated gene delivery. J. Gene Med. *7*, 630–637.

40. Pañeda, A., Vanrell, L., Mauleon, I., Crettaz, J.S., Berraondo, P., Timmermans, E.J., Beattie, S.G., Twisk, J., van Deventer, S., Prieto, J., et al. (2009). Effect of adeno-associated virus serotype and genomic structure on liver transduction and biodistribution in mice of both genders. Hum. Gene Ther. *20*, 908–917.

41. Arruda, V.R., Fields, P.A., Milner, R., Wainwright, L., De Miguel, M.P., Donovan, P.J., Herzog, R.W., Nichols, T.C., Biegel, J.A., Razavi, M., and Dake, M. (2001). Lack of germline transmission of vector sequences following systemic administration of recombinant AAV-2 vector in males. Mol. Ther. *4*, 586–592.

42. Schuettrumpf, J., Liu, J.H., Couto, L.B., Addya, K., Leonard, D.G., Zhen, Z., Summer, J., and Arruda, V.R. (2006). Inadvertent germline transmission of AAV2 vector: findings in a rabbit model correlate with those in a human clinical trial. Mol. Ther. *13*, 1064–1073.

43. Couto, L., Parker, A., and Gordon, J.W. (2004). Direct exposure of mouse spermatozoa to very high concentrations of a serotype-2 adeno-associated virus gene therapy vector fails to lead to germ cell transduction. Hum. Gene Ther. *15*, 287–291.

44. Nakai, H., Iwaki, Y., Kay, M.A., and Couto, L.B. (1999). Isolation of recombinant adeno-associated virus vector-cellular DNA junctions from mouse liver. J. Virol. *73*, 5438–5447.

45. Nakai, H., Yant, S.R., Storm, T.A., Fuess, S., Meuse, L., and Kay, M.A. (2001). Extrachromosomal recombinant adeno-associated virus vector genomes are primarily responsible for stable liver transduction in vivo. J. Virol. *75*, 6969–6976.

46. Scarpa, M., Buffone, E., Marca, P.L., Campello, M., and Rampazzo, A. (2010). Difficulties in diagnosing slowly progressive mucopolysaccharidosis VI: a case series. J. Pediatr. Rehabil. Med. *3*, 71–75.

47. Dilger, H., Leissner, L., Bosanska, L., Lampe, C., and Plöckinger, U. (2013). Illness perception and clinical treatment experiences in patients with M. Maroteaux-Lamy (mucopolysaccharidosis type VI) and a Turkish migration background in Germany. PLoS ONE *8*, e66804.

48. Bacchus, H., and Peterson, D.I. (1980). Pregnancy complicated by myelopathy due to Maroteaux-Lamy syndrome. Am. J. Obstet. Gynecol. *136*, 259–260.

49. Bell, P., Wang, L., Gao, G., Haskins, M.E., Tarantal, A.F., McCarter, R.J., Zhu, Y., Yu, H., and Wilson, J.M. (2011). Inverse zonation of hepatocyte transduction with AAV vectors between mice and non-human primates. Mol. Genet. Metab. *104*, 395–403.

50. Sabatino, D.E., Lange, A.M., Altynova, E.S., Sarkar, R., Zhou, S., Merricks, E.P., Franck, H.G., Nichols, T.C., Arruda, V.R., and Kazazian, H.H., Jr. (2011). Efficacy and safety of long-term prophylaxis in severe hemophilia A dogs following liver gene therapy using AAV vectors. Mol. Ther. *19*, 442–449.

51. Wang, L., Wang, H., Bell, P., McCarter, R.J., He, J., Calcedo, R., Vandenberghe, L.H., Morizono, H., Batshaw, M.L., and Wilson, J.M. (2010). Systematic evaluation of AAV vectors for liver directed gene transfer in murine models. Mol. Ther. *18*, 118–125.

52. Evers, M., Saftig, P., Schmidt, P., Hafner, A., McLoghlin, D.B., Schmahl, W., Hess, B., von Figura, K., and Peters, C. (1996). Targeted disruption of the arylsulfatase B gene results in mice resembling the phenotype of mucopolysaccharidosis VI. Proc. Natl. Acad. Sci. USA *93*, 8214–8219.

53. Chang, P.L., Rosa, N.E., and Davidson, R.G. (1981). Differential assay of arylsulfatase A and B activities: a sensitive method for cultured human cells. Anal. Biochem. *117*, 382–389.

54. de Jong, J.G., Wevers, R.A., Laarakkers, C., and Poorthuis, B.J. (1989). Dimethylmethylene blue-based spectrophotometry of glycosaminoglycans in untreated urine: a rapid screening procedure for mucopolysaccharidoses. Clin. Chem. *35*, 1472–1477.

OMTM, Volume *6*

Supplemental Information

Non-clinical Safety and Efficacy of an AAV2/8 Vector Administered Intravenously for Treatment of Mucopolysaccharidosis Type VI

Rita Ferla, Marialuisa Alliegro, Jean-Brice Marteau, Margherita Dell'Anno, Edoardo Nusco, Severine Pouillot, Stefania Galimberti, Maria Grazia Valsecchi, Vincent Zuliani, and Alberto Auricchio

Figure S1. Body weight changes of C57/BL6-Tg*ARSBC91S* mice treated with AAV2/8.TBG.*hARSB*.

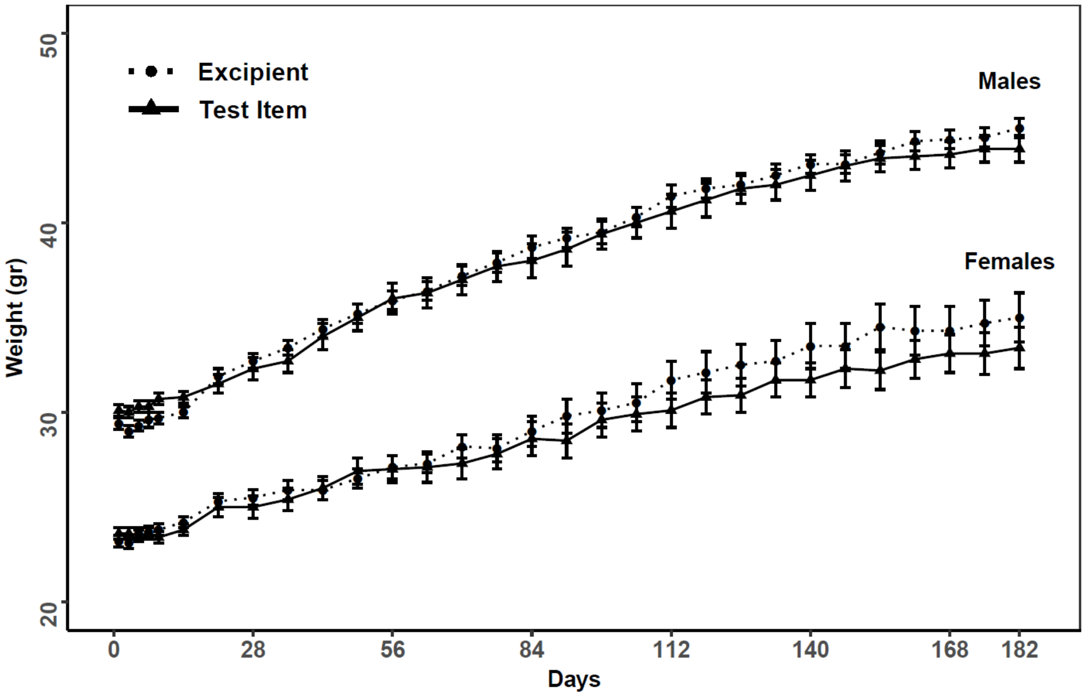

Body weight was recorded on D1 (pre-dose), D3, D5, D7, D9, D14 and then weekly both in male and female mice. Results are reported as mean ± SEM. The comparison of the treatment specific growth curves in males and females was performed by means of an ANOVA for repeated measurements. The interaction time*treatment was not found statistically significant (p-value>0.05). The number of animals is: *Males*, n=46 from D1 to D14, n=26 from D21 to D182 in both the excipient and the test item groups; *Females*: n=46 from D1 to D14, n=26 from D21 to D182 in both the excipient group; n=45 from D1 to D14 (except D3, n=44), n=25 from D21 to D182 in the test item group. *Abbreviations:* Excipient, formulation buffer diluted 1:1.5 in NaCl 0.9% saline solution; Test Item, AAV2/8.TBG.*hARSB* diluted 1:1.5 in NaCl 0.9% saline solution (dose administered $2x10^{13}$ GC/kg).

Table S1. Hematology profile in mice following systemic administration of either AAV2/8.TBG.*hARSB* or the excipient.

	D15		D180	
	Excipient	Test item	Excipient	Test item
**Males**	n=9	n=9	n=10	n=9
WBC	6.60 (5.90-7.70)	7.00 (6.70-7.40)	8.25 (7.30-9.40)	8.30 (7.50-9.40)
NEUT	0.87 ± 0.17	0.70 ± 0.09	0.90 ± 0.19	0.84 ± 0.19
EOSI	0.11 ± 0.04	0.14 ± 0.07	0.14 ± 0.08	0.10 ± 0.03
BASO	0.02 ± 0.01	0.02 ± 0.01	0.03 ± 0.01	0.04 ± 0.01
LYMP	5.60 (4.90-6.80)	5.80 (5.80-6.30)	7.10 (6.10-7.70)	7.10 (6.60-8.10)
MONO	0.06 ± 0.03	0.08 ± 0.03	0.15 ± 0.05	0.10 ± 0.05
LUC	0.06 ± 0.02	0.06 ± 0.02	0.10 ± 0.04	0.11 ± 0.05
RBC	10.78 ± 0.74	11.33 ± 0.54	10.53 ± 0.25	10.73 ± 0.25
HGB	15.41 ± 0.68	15.89 ± 0.90	15.07 ± 0.52	15.16 ± 0.52
HCT	48.67 ± 2.87	51.22 ± 1.72 *	47.10 ± 1.73	48.67 ± 1.58
MCV	45.22 ± 0.97	45.22 ± 0.97	45.00 ± 1.15	45.33 ± 1.00
MCH	14.36 ± 0.89	14.09 ± 1.20	14.34 ± 0.3	14.10 ± 0.23
MCHC	31.67 ± 1.73	31.00 ± 2.50	32.00 ± 0.47	31.22 ± 0.44 **
RET	298.22 ± 34.75	287.22 ± 23.83	292.80 ± 37.07	307.33 ± 21.97
RDW	12.61 ± 0.73	12.11 ± 0.28	13.32 ± 0.58	13.41 ± 0.32
THR	1165 (1109-1292)	1368 (1094-1433)	1211 (1093-1249)	1440 (1373-1543) **
MPV	5.48 ± 0.67	5.57 ± 0.57	5.51 ± 0.59	7.37 ± 0.26 **
**Females**	n=7	n=8	n=10	n=9
WBC	5.80 (3.80-6.10)	5.30 (4.60-6.85)	3.30 (2.50-3.80)	3.30 (2.50-3.40)
NEUT	0.84 ± 0.23	0.82 ± 0.24	0.44 ± 0.14	0.42 ± 0.11
EOSI	0.09 ± 0.08	0.07 ± 0.04	0.04 ± 0.02	0.06 ± 0.04
BASO	0.02 ± 0.01	0.03 ± 0.01	0.02 ± 0.01	0.01 ± 0.01
LYMP	4.70 (3.30-4.90)	4.35 (3.55-5.65)	2.70 (2.20-3.30)	2.5 0(2.00-2.90)
MONO	0.04 ± 0.02	0.05 ± 0.02	0.03 ± 0.02	0.06 ± 0.04
LUC	0.03 ± 0.01	0.03 ± 0.02	0.02 ± 0.01	0.03 ± 0.03
RBC	10.84 ± 0.39	10.83 ± 0.20	10.33 ± 0.27	10.23 ± 0.34
HGB	16.36 ± 0.50	16.15 ± 0.39	14.99 ± 0.53	14.98 ± 0.68
HCT	51.29 ± 2.14	50.88 ± 1.46	46.2 ± 1.55	46.44 ± 1.94
MCV	47.43 ± 0.79	47.00 ± 0.76	44.70 ± 1.06	45.44 ± 0.73
MCH	15.10 ± 0.28	14.93 ± 0.17	14.50 ± 0.29	14.64 ± 0.31
MCHC	32.29 ± 0.49	31.75 ± 0.46	32.50 ± 0.71	32.22 ± 0.67
RET	280.14 ± 37.75	294.75 ± 28.01	308.80 ± 54.07	352.56 ± 55.21
RDW	12.37 ± 0.41	12.18 ± 0.47	12.85 ± 0.57	12.88 ± 0.37
THR	1008 (938-1168)	1083.5 (999-1162)	1213 (1101-1462)	1133 (1122-1344)
MPV	6.06 ± 0.47	5.74 ± 0.65	6.03 ± 0.65	7.23 ± 0.36 **

Hematology was assessed in male and female mice on D15 and D180 by treatment group. Data are reported as mean ± SD and the comparison between treatment groups (at different time points in males and females) were performed by means of the t-test or reported as median (I-III quartile) and compared by means of the Wilcoxon-Mann-Whitney test (for WBC, LYMP and THR). The *p*-value: * ≤ 0.05, ** ≤ 0.01. *Abbreviations and units:* WBC (Giga/L) = white blood cell count; NEUT (Giga/L) = neutrophils; EOSI (Giga/L) = eosinophils; BASO (Giga/L) = basophils; LYMP (Giga/L) = lymphocytes; MONO (Giga/L) = monocytes; LUC (Giga/L) = large unstained cells; RBC (Tera/L) = red blood cell count; HGB (g/100 mL) = haemoglobin; HCT (%) = haematocrit; MCV (fl) = Mean corpuscular volume; MCH (pg) = mean corpuscular haemoglobin; MCHC (g/100 mL) = mean corpuscular haemoglobin concentration; RET (Giga/L) = reticulocytes; RDW (%) = red cell (erythrocyte) volume; THR (Giga/L) = thrombocytes; MPV (%) = mean platelet thrombocyte volume. Excipient, formulation buffer diluted 1:1.5 in NaCl 0.9% saline solution; Test Item, AAV2/8.TBG.*hARSB* diluted 1:1.5 in NaCl 0.9% saline solution (dose administered $2x10^{13}$GC/kg).

Table S2. Blood chemistry profile in mice following systemic administration of either AAV2/8.TBG.*hARSB* or the excipient.

	n	D15<br>Excipient	n	Test item	n	D180<br>Excipient	n	Test item
Males								
ALT	15	51 (39-72)	15	41 (36-61)	16	120 (73-172)	16	155.5 (89-195.50)
AST	15	85 (68-107)	14	79.5 (64-108)	16	105 (89.50-156.50)	16	130 (91.50-155.5)
ALP	15	82 (73-91)	15	89 (81-94)	16	83.50 (69.50-105)	16	98 (86.50-123)
CK	15	384 (261-640)	13	360 (215-519)	16	196 (114-411.50)	16	191 (125.50-301)
ALB	15	28.53 ± 1.46	15	29.8 ± 1.42	16	32.25 ± 1.61	16	32.31 ± 1.08
TBIL	12	1.71 ± 0.27	12	1.86 ± 0.42	15	1.60 ± 0.22	15	1.46 ± 0.20
CHOL	13	2.61 ± 0.27	13	2.45 ± 0.13	16	3.72 ± 0.66	16	3.84 ± 0.85
CREA	13	20 (13-22)	13	17 (15-21)	16	18.5 (15.5-21.5)	16	18.5 (16-22)
GLU	15	11.88 ± 2.12	15	13.44 ± 2.16	16	13.74 ± 2.7	16	13.40 ± 1.87
PROT	15	53.47 ± 3.36	13	58.54 ± 3.57 **	16	58.38 ± 2.03	16	59.38 ± 2.22
TRIG	13	0.92 ± 0.19	13	1.03 ± 0.23	16	0.86 ± 0.12	16	0.91 ± 0.15
UREA	13	7.54 ± 1.31	13	7.76 ± 1.16	16	6.47 ± 1.12	16	6.64 ± 1.55
P	13	2.09 ± 0.36	13	2.11 ± 0.44	16	1.86 ± 0.31	16	1.92 ± 0.26
Cl	13	111.46 ± 3.60	13	111.38 ± 4.75	16	112.69 ± 1.66	16	112.25 ± 4.16
K	13	4.65 ± 0.58	13	4.96 ± 0.71	16	3.86 ± 0.34	16	4.24 ± 0.46
Na	13	150.62 ± 4.31	13	149.62 ± 3.28	16	147 ± 2.90	16	147.75 ± 1.81
GLOB	15	24.93 ± 3.20	13	28.62 ± 3.12 **	16	26.13 ± 1.36	16	27.06 ± 2.14
A/G	15	1.17 ± 0.20	13	1.05 ± 0.10	16	1.24 ± 0.09	16	1.20 ± 0.11
**Females**								
ALT	20	49 (42.50-67)	21	65 (54-94) *	16	52.50 (43.50-70)	16	48.50 (37-72.50)
AST	18	112.50 (76-167)	21	131 (102-166)	16	104.50 (86-116)	16	81.5 (74-107)
ALP	20	131.50 (106-141)	21	122 (115-134)	16	90.5 (79.50-105)	16	93 (78-110.50)
CK	19	819 (350-1713)	18	778.50 (490-1247)	16	262.50 (159-361.50)	16	288.50 (183-317)
ALB	20	30.40 ± 1.70	21	29.76 ± 20	16	30.44 ± 1.26	16	30.94 ± 1.39
TBIL	11	1.66 ± 0.36	10	1.50 ± 0.26	8	1.58 ± 0.69	10	1.74 ± 0.48
CHOL	20	2.29 ± 0.24	21	2.10 ± 0.20 **	16	2.12 ± 0.37	16	2.18 ± 0.27
CREA	12	18.5 (16.50-20.50)	13	15 (15-20)	16	19 (16.50-20.50)	16	19 (16.50-20)
GLU	13	9.40 ± 2.05	13	9.22 ± 2.46	16	9.76 ± 1.51	16	10.63 ± 1.93
PROT	18	55.28 ± 2.44	19	56.21 ± 4.32	16	55.94 ± 2.32	16	56.25 ± 2.79
TRIG	13	0.77 ± 0.22	13	0.82 ± 0.22	16	0.85 ± 0.28	16	0.89 ± 0.37
UREA	13	7.06 ± 1.19	13	7.37 ± 0.76	16	5.73 ± 1.28	16	5.96 ± 1.52
P	13	2.07 ± 0.35	13	2.21 ± 0.31	16	1.94 ± 0.32	16	1.94 ± 0.29
Cl	13	105.85 ± 5.29	13	107.38 ± 5.27	16	109.31 ± 3.18	16	108.25 ± 4.93
K	13	4.77 ± 0.60	13	5.30 ± 0.97	16	3.80 ± 0.31	16	3.98 ± 0.40
Na	13	153.38 ± 5.01	13	147.46 ± 4.61 **	16	146.63 ± 1.82	16	145.38 ± 2.66
GLOB	18	24.78 ± 2.44	19	26.47 ± 3.17	16	25.50 ± 1.79	16	25.31 ± 2.21
A/G	18	1.25 ± 0.16	19	1.13 ± 0.13	16	1.20 ± 0.09	16	1.23 ± 0.11

Blood chemistry was assessed in male and female mice on D15 and D180 by treatment group. Data are reported as mean ± SD and the comparison between treatment groups (at different time points in males and females) were performed by means of the t-test or reported as median (I-III quartile) and compared by means of the Wilcoxon-Mann-Whitney test (for ALT, AST, ALP, CK and CREA) . The *p*-value: $^{*} \leq 0.05$, $^{**} \leq 0.01$. *Abbreviations and units*: ALT (IU/L) = alanine aminotransferase; AST (IU/L) = aspartate aminotransferase; ALP (IU/L) = alkaline phosphatase. CK (IU/L) = creatine kinase; Metabolites and proteins: ALB (g/L) = albumin; TBIL (μmol/L) = total bilirubin; CHOL (mmol/L) = total cholesterol; CREA (μmol/L) = creatinine; GLU (mmol/L) = glucose; PROT (g/L) = total proteins; TRIG (mmol/L) = triglycerides; UREA (mmol/L) = urea; GLOB (g/L) = globulina; A/G = ration albumin/globulina; Electrolytes: P (mmol/L) = phosphorus; Cl (mmol/L) = chloride; K (mmol/L) = potassium; Na (mmol/L) = sodium; Limit of quantification of TBIL: 1.13 μmol/L; Limit of quantification of CREA: 8.7 μmol/L. Excipient, formulation buffer diluted 1:1.5 in saline solution NaCl 0.9%; Test Item, AAV2/8.TBG.*hARSB* diluted 1:1.5 in saline solution NaCl 0.9% (dose administered $2x10^{13}$ GC/kg).

Table S3. Macroscopic findings observed at necropsy

	Organ	Observation	Excipient	Test Item	Notes from Histopathology report
**Males D15**	*Spleen*	Black area (2 mm diameter)	1/5	1/5	Excipient group: no observable microscopic abnormalities.<br>Test item group: mild spleen congestion.
**Males D180**	*Spleen*	Black area (2 mm diameter)	1/5	0/5	Mild spleen congestion
	Liver	Pale	2/5	3/5	Excipient group: moderate (n=1) or marked (n=1) periacinar hepatocytic lipidosis.<br>Test item group: moderate (n=2) or marked (n=1) periacinar hepatocytic lipidosis (n=1).
	Sub-maxillary lymph nodes	Red Dark	1/5	0/5	No observable microscopic abnormalities
**Females D15**	*Spleen*	Black area (2 mm diameter)	1/5	1/5	Excipient group: moderate spleen congestion.<br>Test item group: no observable microscopic abnormalities.
	Gall bladder	Description not available	0/5	1/5	Not available because of an error during harvesting
**Females D180**	*Spleen*	Three black spots	0/5	1/5	Mild spleen congestion
		Black area (2-3 mm diameter)	0/5	2/5	No observable microscopic abnormalities.
		Black area (half surface of the organ)	0/5	1/5	No observable microscopic abnormalities.
	Uterus with uterine cervix	Red Mass (left, 3 mm diameter)	0/5	1/5	Mass diagnosis was adenoma (Benign neoplasm).
		Decreased	1/5	0/5	No observable microscopic abnormalities.
		Enlarged with abnormal content	0/5	1/5	Dilatation
	Vagina	Enlarged with abnormal content	0/5	1/5	Dilatation
	Ovaries	One enlarged	1/5	0/5	No observable microscopic abnormalities.

Abbreviations: Excipient, formulation buffer diluted 1:1.5 in NaCl 0.9% saline solution; Test Item, AAV2/8.TBG.*hARSB* diluted 1:1.5 in NaCl 0.9% saline solution (dose administered $2x10^{13}$ GC/kg). The comparison of macroscopic finding incidence in the two treatment groups at each time point in males and females was performed using the Fisher test (*p*-values >0.05).

Table S4. Incidences and severity of microscopic findings in thyroid of treated mice on D15 and D180

Observation	Sex	Mean severity scores (incidence)			
		D15		D180	
		**Excipient**	**Test item**	**Excipient**	**Test item**
Pallid follicular epithelium hypertrophy	M	*(0/5)*	1.4 *(4/5)**	0.8 *(3/5)*	1.4 *(4/5)*
	F	*(0/5)*	0.4 *(3/5)*	0.4 *(1/5)*	2.468 *(4/5)*
Multifocal follicular epithelial hyperplasia	M	*(0/5)*	*(0/5)*	0.6 *(1/5)*	0.8 *(1/5)*
	F	*(0/5)*	*(0/5)*	0.268 *(1/5)*	2.2 *(3/5)*
Lipid replacement od follicular epithelium	M	*(0/5)*	*(0/5)*	*(0/5)*	0.8 *(3/5)*
	F	*(0/5)*	*(0/5)*	*(0/5)*	*(0/5)*

The mean severity score is calculated as the mean of severity scores of each animal showing the reported observation per each group of treatment. The comparison of microscopic findings incidence in thyroids of treatment groups at each time points in males and females was performed using the Fisher test. The *p*-value vs excipient group: * ≤ 0.05. *Abbreviations:* Excipient, formulation buffer diluted 1:1.5 in NaCl 0.9% saline solution; Test Item, AAV2/8.TBG.*hARSB* diluted 1:1.5 in NaCl 0.9% saline solution (dose administered $2x10^{13}$ GC/kg); M, males; F, females.

Table S5. Vector genome copies in liver, thyroid/parathyroid and pituitary gland from mice of the toxicity study.

Organ	D15		D180	
	Males	Females	Males	Females
Liver	$1.9x10^7±4.9x10^6$	$9.3x10^6±3.3x10^6$	$1.1\ x10^7±3.6x10^6$	$5.6x10^6±1.5x10^6$
Thyroid/parathyroid	$1.7x10^4±8.2x10^3$	$1.1x10^4±3.6\ x10^3$	$3.9x10^3±1.8x10^3$	$7.0x10^3±6.1x10^3$
Pituitary Gland	$6.4x10^3±1.8x10^3$	$1.4x10^4±1.3x10^4$	$1.2x10^3±5.4x10^2$	$4.6x10^3±2.2x10^3$

AAV2/8.TBG.*hARSB* (AAV) DNA is expressed as genome copies (GC) per μg of total DNA (GC/μg of total DNA). Results are reported as mean ± SD. *Abbreviations*: D, study day.

Table S6. AAV2/8.TBG.*hARSB* biodistribution in male mice.

Organ	D15<br>AAV DNA	>LOQ (n)	<LOQ and >LOD (n)	<LOD (n)	D180<br>AAV DNA	> LOQ (n)	<LOQ and >LOD (n)	<LOD (n)
**LI**	$2.22 \times 10^{6} \pm 6.46 \times 10^{5}$	4	0	0	$1.02 \times 10^{6} \pm 3.04 \times 10^{5*}$	5	0	0
**GBL**	$9.79 \times 10^{4} \pm 6.42 \times 10^{4}$	4	0	0	$6.15 \times 10^{4} \pm 7.29 \times 10^{4}$	5	0	0
**AD**	$1.91 \times 10^{4} \pm 7.50 \times 10^{3}$	4	0	0	$4.73 \times 10^{3} \pm 4.91 \times 10^{3*}$	5	0	0
**SP**	$7.79 \times 10^{3} \pm 1.14 \times 10^{4}$	4	0	0	$8.49 \times 10^{1} \pm 2.86 \times 10^{1**}$	2	3	0
**AO**	$4.42 \times 10^{3} \pm 1.23 \times 10^{3}$	4	0	0	$6.47 \times 10^{2} \pm 2.93 \times 10^{2**}$	5	0	0
**BL**	$4.01 \times 10^{3} \pm 1.66 \times 10^{3}$	4	0	0	<LOD**	0	0	5
**KI**	$3.00 \times 10^{3} \pm 1.59 \times 10^{3}$	4	0	0	$1.58 \times 10^{2} \pm 2.66 \times 10^{1**}$	5	0	0
**RE**	$2.26 \times 10^{3} \pm 2.41 \times 10^{2}$	4	0	0	$1.98 \times 10^{2} \pm 1.16 \times 10^{2*}$	4	1	0
**GTHY**	$1.95 \times 10^{3} \pm 1.84 \times 10^{3}$	4	0	0	$1.15 \times 10^{2} \pm 4.99 \times 10^{1**}$	3	2	0
**ILN**	$1.78 \times 10^{3} \pm 5.76 \times 10^{2}$	4	0	0	$6.26 \times 10^{2} \pm 4.13 \times 10^{2**}$	5	0	0
**OES**	$1.52 \times 10^{3} \pm 7.73 \times 10^{2}$	4	0	0	$3.03 \times 10^{2} \pm 1.24 \times 10^{2**}$	5	0	0
**SK**	$1.51 \times 10^{3} \pm 9.13 \times 10^{2}$	4	0	0	$8.47 \times 10^{1} \pm 1.49 \times 10^{1**}$	3	1	1
**GLA**	$1.48 \times 10^{3} \pm 1.03 \times 10^{3}$	4	0	0	$2.34 \times 10^{2} \pm 1.38 \times 10^{1**}$	3	2	0
**HE**	$1.48 \times 10^{3} \pm 5.58 \times 10^{2}$	4	0	0	$2.18 \times 10^{2} \pm 9.69 \times 10^{1**}$	5	0	0
**TRA**	$1.19 \times 10^{3} \pm 7.04 \times 10^{2}$	4	0	0	$9.82 \times 10^{1} \pm 3.74 \times 10^{1**}$	5	0	0
**FOR**	$9.37 \times 10^{2} \pm 7.67 \times 10^{2}$	4	0	0	$9.12 \times 10^{2} \pm 1.11 \times 10^{3}$	4	1	0
**DU**	$8.77 \times 10^{2} \pm 2.97 \times 10^{2}$	4	0	0	$1.11 \times 10^{2} \pm 6.61 \times 10^{1**}$	4	1	0
**SM**	$7.26 \times 10^{2} \pm 3.55 \times 10^{2}$	4	0	0	$6.23 \times 10^{1} \pm 2.16 \times 10^{0**}$	3	2	0
**JE**	$6.69 \times 10^{2} \pm 3.01 \times 10^{2}$	4	0	0	$7.93 \times 10^{1} \pm 1.76 \times 10^{1**}$	4	1	0
**TH**	$6.30 \times 10^{2} \pm 7.25 \times 10^{2}$	4	0	0	4.66×10^{2}	1	3	1
**LTEP**	$6.03 \times 10^{2} \pm 2.71 \times 10^{2}$	4	0	0	$6.48 \times 10^{1**}$	1	4	0
**LTE**	$5.90 \times 10^{2} \pm 2.44 \times 10^{2}$	4	0	0	<LOQ**	0	5	0
**LHEP**	$5.76 \times 10^{2} \pm 1.78 \times 10^{2}$	4	0	0	$6.87 \times 10^{1**}$	1	2	2
**PIT**	$5.53 \times 10^{2} \pm 1.97 \times 10^{2}$	4	0	0	$5.90 \times 10^{1} \pm 2.87 \times 10^{0**}$	2	3	0
**URB**	$5.02 \times 10^{2} \pm 4.17 \times 10^{2}$	4	0	0	$5.42 \times 10^{1**}$	1	0	4
**LU**	$4.90 \times 10^{2} \pm 1.08 \times 10^{2}$	4	0	0	<LOQ**	0	4	1
**IL**	$4.75 \times 10^{2} \pm 2.03 \times 10^{2}$	4	0	0	5.67×10^{1}	1	4	0
**STR**	$4.26 \times 10^{2} \pm 2.35 \times 10^{2}$	4	0	0	$3.89 \times 10^{2} \pm 5.96 \times 10^{2}$	5	0	0
**MLN**	$3.32 \times 10^{2} \pm 2.84 \times 10^{2}$	4	0	0	$1.54 \times 10^{2} \pm 1.30 \times 10^{2*}$	2	3	0
**PRO**	$3.13 \times 10^{2} \pm 1.31 \times 10^{2}$	4	0	0	$9.25 \times 10^{1**}$	1	3	1
**PAN**	$2.85 \times 10^{2} \pm 1.21 \times 10^{2}$	4	0	0	<LOQ**	0	4	1
**SA**	$2.75 \times 10^{2} \pm 1.51 \times 10^{2}$	4	0	0	<LOQ**	0	2	3
**CC**	$2.68 \times 10^{2} \pm 7.51 \times 10^{1}$	4	0	0	$6.43 \times 10^{1**}$	1	4	0
**CO**	$2.44 \times 10^{2} \pm 8.12 \times 10^{1}$	4	0	0	<LOQ**	0	3	2
**PNE**	2.20×10^{2}	*1*	*3*	0	5.74×10^{2}	1	0	4
**STE**	$2.08 \times 10^{2} \pm 7.79 \times 10^{1}$	4	0	0	<LOQ**	0	1	4
**ENC**	$2.07 \times 10^{2} \pm 3.66 \times 10^{1}$	4	0	0	<LOD**	0	0	5
**BM**	$1.48 \times 10^{2} \pm 2.34 \times 10^{1}$	3	1	0	<LOD	0	0	5
**CSC**	$1.35 \times 10^{2} \pm 1.34 \times 10^{1}$	3	1	0	<LOD**	0	0	5
**LSC**	$1.22 \times 10^{2} \pm 4.28 \times 10^{1}$	4	0	0	<LOD**	0	0	5
**LAC**	$1.05 \times 10^{2} \pm 2.26 \times 10^{1}$	4	0	0	<LOQ**	0	1	4
**SV**	$7.69 \times 10^{1} \pm 2.02 \times 10^{1}$	4	0	0	3.63×10^{2}	1	0	4
**EY**	$6.32 \times 10^{1} \pm 9.51 \times 10^{0}$	3	1	0	<LOQ*	0	2	3
**CER**	<LOQ	0	4	0	<LOD**	0	0	5

AAV2/8.TBG.*hARSB* (AAV) DNA is expressed as genome copies (GC) per μg of total DNA (GC/μg of total DNA). Results are reported as mean ± SD of > LOQ values and in a decreasing order on D15. The number (n) of animals analyzed per each time point is 5. One mice sacrificed on D15 was excluded from statistical analysis because data deviate importantly from other animals likely because test item administration was not optimal. The comparison between AAV DNA on D15 vs D180 was performed using the one sided Wilcoxon-Mann-Whitney test, assuming LOQ and LOD values for analysis. The *p*-value vs. D15 is: * <0.05 and ** < 0.01. Samples below the limit of quantification (LOQ) of the assay are reported as <LOQ. The LOQ is 50 GC/μg of DNA except for: *PNE* (LOQ is 167 in 2 out 5 and 249 GC/μg of total DNA in 1 out 5 animals on D15; *CSL* (LOQ is 100 GC/μg of total DNA in 1 out 5 animals on D15). Samples below the limit of detection (LOD) of the assay are reported as <LOQ. The LOD is 15 GC/μg of total DNA except for: *CSC* (LOD is 29.4 in 1 out 5. 49 in 1 out 5 and 30.6 in 1 out 5 animals; *PNE* (LOD is 15.4 in 1 out 5 animals). LOQ and LOD were recalculated based on the amount of DNA analyzed. i.e. when lower than 600 ng. *Abbreviations*: AAV, Adeno-associated viral vector; D, study day; LI, Liver; GBL, Gall bladder; SA, Salivary gland (sublingual); OES, Oesophagus; FOR, Forestomach; GLA,

Glandular stomach; DU, Duodenum; JE, Jejunum; IL, Ileum incl. Peyer's patches; CC, Caecum; CO, Colon; RE, Rectum; PAN, Pancreas; TH, Thymus; SP, Spleen; MLN, Mesenteric lymph node; ILN, Inguinal lymph node; KI, Kidney; URB, Urinary bladder; TE, Testes; HEP, Head of epididymis; TEP, Tail of epididymis; SV, Seminal vesicles (with coagulating gland); PRO, Prostate; GTHY, Thyroids with parathyroids; AD, Adrenal; PIT, Pituitary; HE, Heart; AO, Aorta; TRA, Trachea; LU, Lungs (incl. bronchi and bronchioles); ENC, brain (encephalus); CER, brain (cerebellum); STE, brain stem; CSC, cervical spinal cord; LSC, lumbar spinal cord; PNE, peripheral nerve (sciatic); EY, Eye; LAC, lachrymal gland (harderian gland); SM, skeletal muscle; STR, sternum; SK, skin (incl. subcutaneous tissue); BL, Blood; BM, Bone marrow (from femur).

Table S7. AAV2/8.TBG.*hARSB* biodistribution in female mice.

Organ	D15<br>AAV DNA	>LOQ (n)	<LOQ and >LOD (n)	<LOD (n)	D180<br>AAV DNA	> LOQ (n)	<LOQ and >LOD (n)	<LOD (n)
**LI**	$7.77\times10^{5}\pm2.06\times10^{5}$	5	0	0	$2.89\times10^{5}\pm9.07\times10^{4**}$	5	0	0
**GBL**	$4.08\times10^{4}\pm4.13\times10^{4}$	5	0	0	$3.13\times10^{4}\pm2.34\times10^{4}$	5	0	0
**AD**	$2.92\times10^{4}\pm1.00\times10^{4}$	5	0	0	$4.59\times10^{2}\pm2.64\times10^{2**}$	5	0	0
**ILN**	$8.26\times10^{3}\pm4.85\times10^{3}$	5	0	0	$1.36\times10^{3}\pm8.73\times10^{2**}$	5	0	0
**AO**	$7.84\times10^{3}\pm6.64\times10^{3}$	5	0	0	$9.48\times10^{2}\pm4.88\times10^{2**}$	5	0	0
**BL**	$7.37\times10^{3}\pm3.80\times10^{3}$	5	0	0	<LOD**	0	0	5
**KI**	$3.92\times10^{3}\pm2.42\times10^{3}$	5	0	0	$2.10\times10^{2}\pm8.62\times10^{1**}$	5	0	0
**SP**	$3.48\times10^{3}\pm3.61\times10^{3}$	5	0	0	$5.12\times10^{1**}$	1	4	0
**UTE**	$3.36\times10^{3}\pm1.28\times10^{3}$	5	0	0	$1.29\times10^{2}\pm7.69\times10^{1**}$	3	2	0
**RE**	$3.22\times10^{3}\pm1.96\times10^{3}$	5	0	0	$3.60\times10^{2}\pm2.09\times10^{2**}$	5	0	0
**HE**	$3.21\times10^{3}\pm1.30\times10^{3}$	5	0	0	$2.38\times10^{2}\pm1.42\times10^{2**}$	5	0	0
**VAG**	$3.12\times10^{3}\pm1.64\times10^{3}$	5	0	0	$2.49\times10^{2}\pm1.32\times10^{2**}$	4	0	0
**SM**	$2.97\times10^{3}\pm1.79\times10^{3}$	5	0	0	$1.14\times10^{2}\pm5.98\times10^{1**}$	5	0	0
**SK**	$2.87\times10^{3}\pm1.56\times10^{3}$	5	0	0	$1.26\times10^{2}\pm5.29\times10^{1**}$	3	2	0
**MAM**	$2.68\times10^{3}\pm7.06\times10^{2}$	5	0	0	$1.02\times10^{2}\pm6.44\times10^{1**}$	3	2	0
**LOV**	$2.11\times10^{3}\pm8.73\times10^{2}$	5	0	0	$1.80\times10^{2**}$	1	3	1
**STR**	$2.11\times10^{3}\pm2.68\times10^{3}$	5	0	0	$3.87\times10^{2}\pm4.33\times10^{2*}$	5	0	0
**GLA**	$2.09\times10^{3}\pm3.29\times10^{3}$	5	0	0	$5.90\times10^{2}\pm1.00\times10^{3*}$	5	0	0
**FOR**	$1.61\times10^{3}\pm6.93\times10^{2}$	5	0	0	$2.34\times10^{2}\pm1.86\times10^{2**}$	5	0	0
**MLN**	$1.36\times10^{3}\pm1.55\times10^{3}$	5	0	0	$1.10\times10^{2}\pm4.40\times10^{1**}$	5	0	0
**GTHY**	$1.34\times10^{3}\pm3.94\times10^{3}$	5	0	0	$1.46\times10^{2}\pm6.09\times10^{1**}$	4	1	0
**TRA**	$1.32\times10^{3}\pm4.18\times10^{2}$	5	0	0	$2.49\times10^{2}\pm8.87\times10^{1**}$	5	0	0
**OES**	$1.29\times10^{3}\pm2.49\times10^{2}$	5	0	0	$2.78\times10^{2}\pm1.26\times10^{2**}$	5	0	0
**PIT**	$1.26\times10^{3}\pm6.11\times10^{2}$	5	0	0	$1.08\times10^{2}\pm5.50\times10^{1**}$	4	1	0
**DU**	$9.04\times10^{2}\pm2.16\times10^{2}$	5	0	0	$1.27\times10^{2}\pm6.26\times10^{1**}$	4	1	0
**LU**	$8.49\times10^{2}\pm5.15\times10^{2}$	5	0	0	<LOQ**	0	5	0
**JE**	$8.21\times10^{2}\pm5.11\times10^{1}$	5	0	0	$9.89\times10^{1}\pm3.67\times10^{1**}$	5	0	0
**IL**	$7.37\times10^{2}\pm1.42\times10^{2}$	5	0	0	$7.39\times10^{1}\pm8.79\times10^{0**}$	3	2	0
**PAN**	$7.11\times10^{2}\pm3.09\times10^{2}$	5	0	0	$6.70\times10^{1**}$	1	2	2
**URB**	$5.31\times10^{2}\pm9.86\times10^{1}$	5	0	0	6.71×10^{3}	1	4	0
**PNE**	$4.89\times10^{2}\pm1.34\times10^{2}$	5	0	0	$6.96\times10^{1**}$	1	2	2
**CC**	$4.57\times10^{2}\pm9.80\times10^{1}$	5	0	0	$6.36\times10^{1}\pm4.96\times10^{0**}$	2	3	0
**SA**	$3.59\times10^{2}\pm3.61\times10^{1}$	5	0	0	$8.94\times10^{1}\pm4.12\times10^{1**}$	2	2	1
**CO**	$3.56\times10^{2}\pm6.95\times10^{1}$	5	0	0	$7.73\times10^{1}\pm1.36\times10^{1**}$	3	2	0
**ENC**	$2.34\times10^{2}\pm3.40\times10^{1}$	5	0	0	<LOQ**	0	2	3
**LAC**	$2.17\times10^{2}\pm7.50\times10^{1}$	5	0	0	<LOQ**	0	3	2
**STE**	$2.15\times10^{2}\pm9.07\times10^{1}$	5	0	0	<LOQ**	0	2	3
**BM**	$1.30\times10^{2}\pm4.25\times10^{1}$	5	0	0	<LOQ**	0	3	2
**LSC**	$1.26\times10^{2}\pm3.88\times10^{1}$	4	1	0	<LOD**	0	0	5
**EY**	$1.20\times10^{2}\pm4.56\times10^{1}$	5	0	0	<LOD**	0	0	5
**CSC**	$1.15\times10^{2}\pm2.66\times10^{1}$	5	0	0	<LOQ**	0	1	3
**TH**	$1.01\times10^{2}\pm3.45\times10^{1}$	3	2	0	<LOQ*	0	3	2
**CER**	$6.33\times10^{1}\pm4.26\times10^{0}$	2	3	0	<LOD**	0	0	5

AAV2/8.TBG.*hARSB* (AAV) DNA is expressed as genome copies (GC) per μg of total DNA (GC/μg of total DNA). Results are reported as mean ± SD of > LOQ values and in a decreasing order on D15. The number (n) of animals analyzed per each time point is 5, except for *CSC* and *VAG* in females on D180 (n=4). The comparison between AAV DNA on D15 vs D180 was performed using the one sided Wilcoxon-Mann-Whitney test, assuming LOQ and LOD values for analysis. The *p*-value vs. D15 is: * <0.05 and ** < 0.01. Samples below the limit of quantification (LOQ) of the assay are reported as <LOQ. The LOQ is 50 GC/μg of total DNA except for: *CSL* (LOQ is 100 GC/μg of total DNA in 1 out 5 animals on D15); *PNE* (LOQ is 53 GC/μg of total DNA in 1 out 5 animals on D15). Samples below the limit of detection (LOD) of the assay are reported as <LOD. The LOD is 15 GC/μg of total DNA except for: *CSC* (LOD is 48 GC/μg of total DNA in out of 5. 75 GC/μg of total DNA in 1 out 5 and 29 GC/μg of total DNA in 1 out 5 animals on D180); *PNE* (LOD is 21 GC/μg of total DNA in out of 5). LOQ and LOD were recalculated based on the amount of DNA analyzed. i.e. when lower than 600 ng. *Abbreviations*: AAV, Adeno-associated viral vector; D, study day; LI, Liver; GBL, Gall bladder; SA, Salivary gland (sublingual); OES, Oesophagus; FOR, Forestomach; GLA, Glandular stomach; DU, Duodenum; JE, Jejunum; IL, Ileum incl. Peyer's patches; CC, Caecum; CO, Colon; RE, Rectum; PAN, Pancreas; TH, Thymus; SP, Spleen; MLN, Mesenteric lymph node; ILN, Inguinal lymph node; KI, Kidney; URB, Urinary bladder; OV, Ovaries (with

Fallopian tubes); VAG, Vagina; UTE, Uterus (with uterine cervix); GTHY, Thyroids with parathyroids; AD, Adrenal; PIT, Pituitary; HE, Heart; AO, Aorta; TRA, Trachea; LU, Lungs (incl. bronchi and bronchioles); ENC, brain (encephalus); CER, brain (cerebellum); STE, brain stem; CSC, cervical spinal cord; LSC, lumbar spinal cord; PNE, peripheral nerve (sciatic); EY, Eye; LAC, lachrymal gland (harderian gland); SM, skeletal muscle; STR, sternum; SK, skin (incl. subcutaneous tissue); MAM, mammary gland; BL, Blood; BM, Bone marrow (from femur).

Table S8. AAV2/8.TBG.*hARSB* shedding in mice

	Males					Females				
**Time points**	**n**	**AAV DNA**	**>LOQ (n)**	**<LOQ and >LOD (n)**	**<LOD (n)**	**n**	**AAV DNA**	**> LOQ (n)**	**<LOQ and >LOD (n)**	**<LOD (n)**
**STOOL**										
**D2**	11	$1.25x10^{5}\pm1.43x10^{5}$	11	0	0	16	$6.70x10^{4}\pm9.13x10^{4}$	16	0	0
**D4**	11	$8.77x10^{3}\pm3.66x10^{3\,**}$	11	0	0	16	$1.26x10^{4}\pm7.77x10^{3**}$	16	0	0
**D11**	11	$6.10x10^{1}\pm5.66x10^{0\,**}$	2	3	6	16	$7.90x10^{1**}$	1	1	14
**D14**	10	$4.70x10^{2\,**}$	1	1	8	13	<LOD**	0	0	13
**D22**	7	<LOD**	0	0	7	11	<LOD**	0	0	11
**URINE**										
**D2**	5	$2.26x10^{2}\pm1.75x10^{1}$	2	1	2	6	$3.70x10^{2}\pm1.25x10^{2}$	3	2	1
**D4**	6	<LOD	0	0	6	6	< LOQ	0	1	5
**D11**	6	<LOD	0	0	6	6	$2.90x10^{2}$	1	0	5
**D14**	6	<LOD	0	0	6	6	<LOD	0	0	6
**D22**	4	<LOD	0	0	4	4	<LOQ	0	1	3
**D37**	-	-	-	-	-	2	<LOQ	0	1	1
**D60**	-	-	-	-	-	1	<LOD	0	0	1
**PLASMA**										
**D2**	7	$1.28x10^{7}\pm1.22x10^{6}$	7	0	0	8	$1.90x10^{7}\pm5.14x10^{6}$	8	0	0
**D9**	8	$1.02x10^{3}\pm6.38x10^{2**}$	7	1	0	8	$8.47x10^{2}\pm7.39x10^{2**}$	3	5	0
**D15**	4	$3.38x10^{2}\pm2.06x10^{2**}$	4	0	0	5	$4.05x10^{2}\pm1.81x10^{2**}$	4	1	0
**D23**	6	<LOQ**	0	5	1	6	<LOQ**	0	6	0
**D37**	6	<LOQ	0	1	5	6	<LOD**	0	0	6
**D60**	1	<LOD	0	0	1	-	-	-	-	-

AAV2/8.TBG.*hARSB* (AAV) DNA is expressed as genome copies per µg of total DNA (GC/µg of total DNA) for stool and GC per 100 µl of fluid (GC/100 µl) for urine and plasma. Results are reported as mean ± SD of > LOQ values. The limit of quantification (LOQ) and detection (LOD) of the assay are 50 GC/µg of total DNA and 15 GC/µg of total DNA for stool and 30 GC/well and 9 GC/well for urine and plasma. LOQ and LOD were recalculated based on the amount of DNA or the volume of sample analyzed. The comparison of AAV-DNA concentrations over time was performed, in males and females, by means of an ANOVA for repeated measurements. As the time effect was found statistically significant (p-value<0.01), the post-hoc paired comparison vs D2 were done with the Dunnett test. The *p*-value is: * <0.05 and ** < 0.01.*Abbreviations*: AAV, Adeno-associated viral vector; D, study day; - , not available.

Table S9. AAV2/8.TBG.*hARSB* expression in male mice

Organ	D15<br>AAV RNA	>LOQ (n)	<LOQ and >LOD (n)	<LOD (n)	D180<br>AAV RNA	> LOQ (n)	<LOQ and >LOD (n)	<LOD (n)
**LI**	$2.05x10^{7}\pm2.42x10^{6}$	5	0	0	$5.64x10^{6}\pm2.14\ x10^{6**}$	5	0	0
**GBL**	$8.76x10^{6}\pm3.73x10^{6}$	5	0	0	$2.53x10^{6}\pm9.56\ x10^{5**}$	5	0	0
**CSC**	$4.27x10^{5}\pm6.47x10^{5}$	3	0	2	<LOD	0	0	3
**GLA**	$9.94x10^{4}\pm1.85x10^{5}$	4	1	0	$2.95x10^{3}$	1	2	2
**AD**	$5.50x10^{4}\pm5.73x10^{4}$	2	0	3	$6.72x10^{4}\pm3.79x10^{4}$	5	0	0
**TRA**	$2.07x10^{4}\pm2.82x10^{4}$	3	2	0	$3.21x10^{3}\pm3.26\ x10^{3}$	2	1	2
**PNE**	$1.47x10^{4}$	1	0	4	<LOD	0	0	5
**EY**	$1.12x10^{4}$	1	0	4	<LOQ	0	1	2
**DU**	$1.06x10^{4}\pm1.16x10^{4}$	2	0	3	$1.15x10^{3}$	1	1	3
**FOR**	$9.54x10^{3}\pm9.78x10^{3}$	5	0	0	<LOQ*	0	3	2
**STE**	$9.34x10^{3}\pm5.98x10^{2}$	2	1	2	<LOD	0	0	3
**TH**	$7.77x10^{3}\pm6.87x10^{3}$	2	2	1	$5.17x10^{3}\pm5.80\ x10^{3}$	4	0	1
**ENC**	$6.57x10^{3}\pm2.04x10^{3}$	2	0	3	<LOQ	0	1	2
**ILN**	$3.86x10^{3}\pm1.59x10^{3}$	4	0	1	$7.83x10^{3}\pm7.34\ x10^{3}$	3	2	0
**SP**	$3.36x10^{3}$	1	2	2	<LOQ	0	1	4
**HE**	$2.52x10^{3}\pm3.12x10^{1}$	2	0	3	<LOQ	0	1	2
**PIT**	$2.06x10^{3}$	1	0	4	<LOQ	0	1	4
**CC**	$1.09x10^{3}$	1	0	4	<LOD	0	0	3
**STR**	$9.02x10^{2}\pm2.65x10^{2}$	2	2	1	$1.07x10^{3}$	1	2	2
**URB**	$8.90x10^{2}$	1	1	3	<LOD	0	0	3
**OES**	$8.06x10^{2}$	1	1	3	$1.00x10^{4}$	1	2	2
**LU**	$7.37x10^{2}$	1	1	3	$1.04x10^{3}$	1	1	3
**AO**	<LOQ	0	4	1	$1.36x10^{3}$	1	4	0
**PAN**	<LOQ	0	3	0	-	-	-	-
**CO**	<LOQ	0	2	1	-	-	-	-
**MLN**	<LOQ	0	2	1	-	-	-	-
**KI**	<LOQ	0	1	2	-	-	-	-
**LHEP**	<LOQ	0	1	2	-	-	-	-
**LTEP**	<LOQ	0	1	2	-	-	-	-
**GTHY**	<LOQ	0	1	4	-	-	-	-
**SM**	<LOQ	0	1	2	-	-	-	-
**LAC**	<LOD	0	0	5	<LOQ	0	1	2
**RE**	<LOD	0	0	3	-	-	-	-
**SA**	<LOD	0	0	3	-	-	-	-
**JE**	<LOD	0	0	5	<LOD	0	0	3
**IL**	<LOD	0	0	3	-	-	-	-
**LTE**	<LOD	0	0	3	-	-	-	-
**RTE**	<LOD	0	0	3	-	-	-	-
**PRO**	<LOD	0	0	3	-	-	-	-
**SV**	<LOD	0	0	3	-	-	-	-
**RHEP**	<LOD	0	0	3	-	-	-	-
**RTEP**	<LOD	0	0	3	-	-	-	-
**CER**	<LOD	0	0	3	-	-	-	-
**LCS**	<LOD	0	0	5	-	-	-	-
**SK**	<LOD	0	0	5	-	-	-	-
**BL**	<LOD	0	0	5	-	-	-	-
**BM**	<LOD	0	0	5	-	-	-	-

Expression of *hARSB* from AAV2/8.TBG.*hARSB* (AAV RNA) is reported as RNA copy number per µg of total RNA (RNA copy number/µg of total RNA). Results are reported as mean ± SD of > LOQ values and in a decreasing order based on results obtained on D15. The number (n) of animals analyzed per each time point was defined according to the scheme reported in the Material and Methods section: n= 5 for LI, GBL, OES, FOR, GLA, DU, JE, CC, TH, SP, ILN, URB, GTHY, AD, PIT, HE, AO, TRA, LU, ENC, STE, PNE, LAC, CSC, LSC, EY, SK, STR, BL, BM and n=3 in remaining organs on D15; n= 5 in LI, GBL, OES, FOR, GLA, DU, TH, SP, ILN, AO, TRA, LU, PNE and n=3 in JE, CC, URB, HE, ENC, STE, LSC, CSC, EYE, while the remaining organs were not analyzed on D180; The comparison between AAV RNA on D15 vs D180 was performed using the one-sided Wilcoxon-Mann-Whitney test, assuming LOQ and LOD values for analysis. The *p*-value vs. D15 is: ** < 0.01. Samples below the limit of quantification (LOQ) of the assay are reported as <LOQ. The LOQ is 625 RNA copy number /µg of total RNA. Samples below the limit of detection (LOD) of the assay are reported as <LOD. The LOD is 187.5 RNA copy number/µg of

total RNA. LOQ and LOD were recalculated based on the amount of RNA analyzed. i.e. when lower than 600 ng. *Abbreviations*: AAV, Adeno-associated viral vector; D, study day; LI, Liver; GBL, Gall bladder; SA, Salivary gland (sublingual); OES, Oesophagus; FOR, Forestomach; GLA, Glandular stomach; DU, Duodenum; JE, Jejunum; IL, Ileum incl. Peyer's patches; CC, Caecum; CO, Colon; RE, Rectum;, PAN, Pancreas; TH, Thymus; SP, Spleen; MLN, Mesenteric lymph node; ILN, Inguinal lymph node; KI, Kidney; URB, Urinary bladder;; TE, Testes; HEP, Head of epididymis; TEP, Tail of epididymis; SV, Seminal vesicles (with coagulating gland); PRO, Prostate; GTHY, Thyroids with parathyroids; AD, Adrenal; PIT, Pituitary; HE, Heart; AO, Aorta; TRA, Trachea; LU, Lungs (incl. bronchi and bronchioles); ENC, brain (encephalus); CER, brain (cerebellum); STE, brain stem; CSC, cervical spinal cord; LSC, lumbar spinal cord; PNE, peripheral nerve (sciatic); EY, Eye; LAC, lachrymal gland (harderian gland); SM, skeletal muscle; STR, sternum; SK, skin (incl. subcutaneous tissue); BL, Blood; BM, Bone marrow (from femur); - not analyzed on D180.

Table S10. AAV2/8.TBG.*hARSB* expression in female mice

Organ	D15 AAV RNA	D15 >LOQ (n)	D15 <LOQ and >LOD (n)	D15 <LOD (n)	D180 AAV RNA	D180 >LOQ (n)	D180 <LOQ and >LOD (n)	D180 <LOD
**LI**	$7.73x10^6±1.81x10^6$	5	0	0	$3.15x10^6±2.18x10^{6**}$	5	0	0
**GBL**	$2.68x10^6±1.59x10^6$	5	0	0	$1.07x10^6±8.92x10^5$	5	0	0
**CSC**	$2.78x10^4±2.87x10^4$	3	0	2	<LOD	0	0	3
**GLA**	$2.39x10^4±4.29x10^4$	5	0	0	$2.75x10^4±3.35x10^4$	3	0	2
**FOR**	$6.63x10^3±5.91x10^3$	4	1	0	$9.84x10^2±3.83x10^2$	3	0	2
**LU**	$2.78x10^3±9.97x10^2$	3	1	1	<LOQ	0	2	3
**SP**	$2.10x10^3±3.44x10^2$	3	2	0	$7.36x10^2$	1	0	4
**TRA**	$2.02x10^3±1.10x10^3$	3	0	2	$8.30x10^{2*}$	1	4	0
**STE**	$1.61x10^3$	1	1	3	<LOD	0	0	3
**ILN**	$1.51x10^3$	1	1	3	$3.57x10^3±1.80x10^2$	5	0	0
**AO**	$1.13x10^3±4.93x10^2$	3	1	1	$1.41x10^3±4.78x10^2$	3	1	1
**STR**	$1.09x10^3±3.47x10^2$	2	2	1	$1.31x10^3±4.72x10^2$	2	0	3
**LAC**	$1.06x10^3$	1	1	3	<LOD	0	0	3
**JE**	$1.02x10^3$	1	1	3	<LOQ	0	1	2
**ENC**	$9.99x10^2$	1	1	3	<LOD	0	0	3
**DU**	<LOQ	0	2	3	<LOD	0	0	5
**CO**	<LOQ	0	2	1	-	-	-	-
**MLN**	<LOQ	0	2	1	-	-	-	-
**HE**	<LOQ	0	2	3	<LOQ	0	1	2
**SA**	<LOQ	0	1	2	-	-	-	-
**EY**	<LOQ	0	1	4	<LOD	0	0	3
**MAM**	<LOQ	0	1	4	-	-	-	-
**TH**	<LOD	0	0	5	$1.55x10^3±4.10x10^2$	2	1	2
**OES**	<LOD	0	0	5	<LOQ	0	2	3
**PIT**	<LOD	0	0	5	<LOQ	0	1	2
**RE**	<LOD	0	0	3	-	-	-	-
**IL**	<LOD	0	0	3	-	-	-	-
**CC**	<LOD	0	0	5	<LOD	0	0	3
**PAN**	<LOD	0	0	3	-	-	-	-
**URB**	<LOD	0	0	5	<LOD	0	0	3
**KI**	<LOD	0	0	3	-	-	-	-
**LOV**	<LOD	0	0	3	-	-	-	-
**ROV**	<LOD	0	0	3	-	-	-	-
**VAG**	<LOD	0	0	3	-	-	-	-
**UTE**	<LOD	0	0	3	-	-	-	-
**GTHY**	<LOD	0	0	4	-	-	-	-
**AD**	<LOD	0	0	3	-	-	-	-
**CER**	<LOD	0	0	3	-	-	-	-
**LCS**	<LOD	0	0	5	<LOD	0	0	3
**PNE**	<LOD	0	0	3	-	-	-	-
**SK**	<LOD	0	0	3	-	-	-	-
**SM**	<LOD	0	0	3	-	-	-	-
**BL**	<LOD	0	0	5	-	-	-	-
**BM**	<LOD	0	0	5	-	-	-	-

Expression of *hARSB* from AAV2/8.TBG.*hARSB* (AAV RNA) is reported as RNA copy number per µg of total RNA (RNA copy number/µg of total RNA). Results are reported as mean ± SD of > LOQ values and in a decreasing order based on results obtained on D15. The number (n) of animals analyzed per each time point was defined according to the scheme reported in the Material and Methods section: n=5 for LI, GBL, OES, FOR, GLA, DU, JE, CC, TH, SP, ILN, URB, AD, PIT, HE, AO, TRA, LU, ENC, STE, PNE, LAC, CSC, EY, MAM, STR, BL, BM, n=4 for GTHY (one animal is missing because sample was not collected) and n=3 in the remaining organs on D15; n=5 for LI, GBL, OES, FOR, GLA, DU, TH, SP, ILN, AO, TRA, LU and n=3 in JE, CC, URB, PIT, HE, ENC, STE, PNE, LAC, CSC, EYE, while the remaining organs were not analyzed_on D180. The comparison between AAV RNA on D15 vs D180 was performed using the one-sided Wilcoxon-Mann-Whitney test, assuming LOQ and LOD values for analysis. The *p*-value vs. D15 is: ** < 0.01. Samples below the limit of quantification (LOQ) of the assay are reported as <LOQ. The LOQ is 625 RNA copy number /µg of total RNA. Samples below the limit of detection (LOD) of the assay are reported as <LOD. The LOD is 187.5 RNA copy number/µg of total RNA. LOQ and LOD were recalculated based on the amount of RNA analyzed. i.e. when lower than 600 ng. *Abbreviations*: AAV, Adeno-associated viral vector; D, study day; LI, Liver; GBL, Gall bladder; SA, Salivary gland (sublingual); OES, Oesophagus; FOR, Forestomach; GLA, Glandular stomach; DU, Duodenum; JE, Jejunum; IL, Ileum

incl. Peyer's patches; CC, Caecum; CO, Colon; RE, Rectum;, PAN, Pancreas; TH, Thymus; SP, Spleen; MLN, Mesenteric lymph node; ILN, Inguinal lymph node; KI, Kidney; URB, Urinary bladder; OV, Ovaries (with Fallopian tubes); VAG, Vagina; UTE, Uterus (with uterine cervix); GTHY, Thyroids with parathyroids; AD, Adrenal; PIT, Pituitary; HE, Heart; AO, Aorta; TRA, Trachea; LU, Lungs (incl. bronchi and bronchioles); ENC, brain (encephalus); CER, brain (cerebellum); STE, brain stem; CSC, cervical spinal cord; LSC, lumbar spinal cord; PNE, peripheral nerve (sciatic); EY, Eye; LAC, lachrymal gland (harderian gland); SM, skeletal muscle; STR, sternum; SK, skin (incl. subcutaneous tissue); BL, Blood; BM, Bone marrow (from femur); - not analyzed on D180.

Table S11. Phenotype improvement and liver genome copies in transgenic MPS VI mice treated with AAV2/8.TBG.*hARSB*

Groups	Serum ARSB	Liver AAV GC	ARSB	GAGs	Kidney ARSB	GAGs	Spleen ARSB	GAGs
**NR**	13335±1216**	-	106.6±15.6	4.2±0.4**	110.7±18.8**	8.3±1.0**	74.3±5.7**	3.1±0.5**
**AF**	0±0	-	0.0±0.0	46.4±5.8	0.0±0.0	55.3±7.2	0.0±0.0	35.2±2.1
**AAV 2x10^{11}**	119±93	0.018±0.004	8.2±2.6	2.9±1.0**	1.9±0.4	27.4±5.2**	6.7±0.1	5.6±1.7**
**AAV 6x10^{11}**	1082±388	0.231±0.111	39.4±20.5	3.7±1.5**	1.2±0.1	14.6±4.5**	6.1±0.5	2.3±0.5**
**AAV 2x10^{12}**	8714±2745*	2.509±1.182	62.9±12.2	2.8±1.0**	1.5±0.1	8.5±1.7**	7.4±0.8	2.5±0.5**

Serum ARSB is expressed as pg/ml. The average of the mean serum ARSB measured over time in each animal is reported per each group. The number (*n*) of animals is: NR, n=15; AF, n=4; AAV 2x10^{11}, n=4; AAV 6x10^{11}, n= 5; AAV 2x10^{12}, n= 5. AAV GC are expressed as genome copies/molecules of diploid genome. ARSB activity in tissues is expressed as nmol/mg protein/hour. Tissue GAG levels are expressed as μg/mg of protein. The number (*n*) of animals is: NR, n=14; AF, n=8; AAV 2x10^{11}, n=4; AAV 6x10^{11}, n= 4; AAV 2x10^{12}, n=5. All results are represented as mean ± SEM. The comparison between the 5 groups was performed using a one-way ANOVA. In case of a significant global ANOVA test, Tukey-*post hoc* test was used for paired comparisons. The *p*-value vs AF is: *<0.05 and ** ≤0.01. *Abbreviations:* AAV: Adeno-associated viral vector; GC: genome copies; NR: normal mice; AF: MPS VI affected mice; AAV 2x10^{11}: MPS VI affected mice which received 2x10^{11} GC/kg AAV2/8.TBG.*hARSB*; AAV 6x10^{11}: MPS VI affected mice which received 6x10^{11} GC/kg AAV2/8.TBG.*hARSB*; AAV 2x10^{12}: MPS VI affected mice which received 2x10^{12} GC/kg AAV2/8.TBG.*hARSB*; -: below limit of quantification.
